# RNA sequencing identifies specific PIWI-interacting small non-coding RNA expression patterns in breast cancer

**DOI:** 10.18632/oncotarget.2476

**Published:** 2014-09-16

**Authors:** Adnan Hashim, Francesca Rizzo, Giovanna Marchese, Maria Ravo, Roberta Tarallo, Giovanni Nassa, Giorgio Giurato, Gianluca Santamaria, Angela Cordella, Concita Cantarella, Alessandro Weisz

**Affiliations:** ^1^ Laboratory of Molecular Medicine and Genomics, Faculty of Medicine and Surgery, University of Salerno, Baronissi, SA, Italy; ^2^ Fondazione IRCCS SDN, Napoli, Italy; ^3^ Consiglio per la Ricerca e la Sperimentazione in Agricoltura, Centro di Ricerca per l'Orticoltura, Pontecagnano, SA, Italy; ^4^ Division of Molecular Pathology and Medical Genomics, ‘SS. Giovanni di Dio e Ruggi d'Aragona’ Hospital, University of Salerno, Salerno, Italy

**Keywords:** Breast cancer, PIWI, piRNAs, RNA-sequencing, Small non-coding RNA, Estrogen receptor

## Abstract

PIWI-interacting small non-coding RNAs (piRNAs) are genetic and epigenetic regulatory factors in germline cells, where they maintain genome stability, are involved in RNA silencing and regulate gene expression. We found that the piRNA biogenesis and effector pathway are present in human breast cancer (BC) cells and, analyzing smallRNA-Seq data generated from BC cell lines and tumor biopsies, we identified >100 BC piRNAs, including some very abundant and/or differentially expressed in mammary epithelial compared to BC cells, where this was influenced by estrogen or estrogen receptor β, and in cancer respect to normal breast tissues. A search for mRNAs targeted by the BC piRNome revealed that eight piRNAs showing a specific expression pattern in breast tumors target key cancer cell pathways. Evidence of an active piRNA pathway in BC suggests that these small non-coding RNAs do exert transcriptional and post-transcriptional gene regulatory actions also in cancer cells.

## INTRODUCTION

piRNAs are ~24-32nt long non-coding RNAs whose name derive by the fact that they only associate with the PIWI subfamily of Argonaute proteins, first identified in a genetic screen for mutants affecting asymmetric division of stem cells in the *Drosophila* germline [1]. piRNAs do not share common sequence features with each other, except a uridine often present at the 5′ end and a 2′-O-methyl modification on the 3′ nucleotide. They have been initially detected in germline cells, although they have been found expressed also in stem and other somatic cell types [2, 3]. Mature piRNAs are known to derive from post-transcriptional processing of precursor RNAs by two distinct mechanisms. A primary maturation pathway involves cleavage by the PIWI proteins of long single-stranded RNAs transcribed from genomic ‘piRNA clusters’ present in both intra- and inter-genic regions. A secondary pathway consists, instead, of an auto-amplification loop, termed “ping-pong” cycle, in which an antisense piRNA bound to Aubergine and PIWI proteins triggers production of a sense piRNA bound to Argonaute 3 that, in turn, produce a second antisense piRNA by cleavage of highly abundant precursors, such as those produced by expression of transposons and other repetitive genomic sequences [4]. These RNAs are involved in maintaining genome stability by suppressing transposon activity, assembly of the telomere protection complex, RNA silencing and epigenetic control of gene expression by establishment of a repressive chromatin state [5-7]. Given their activity, it is not surprising that piRNAs are being found also in somatic cells [8, 9], although their role(s) here is not yet fully understood. By direct small RNA sequencing we analyzed piRNA expression patterns in human breast cancer (BC) cells and analyzed the possibility that these are modified by neoplastic transformation and, in hormone-responsive BC cells, by estrogen deprivation or expression of the oncosuppressor and sncRNA regulator ERβ [10].

## RESULTS

### The PIWI/piRNA pathway in BC cells

**Figure 1:**
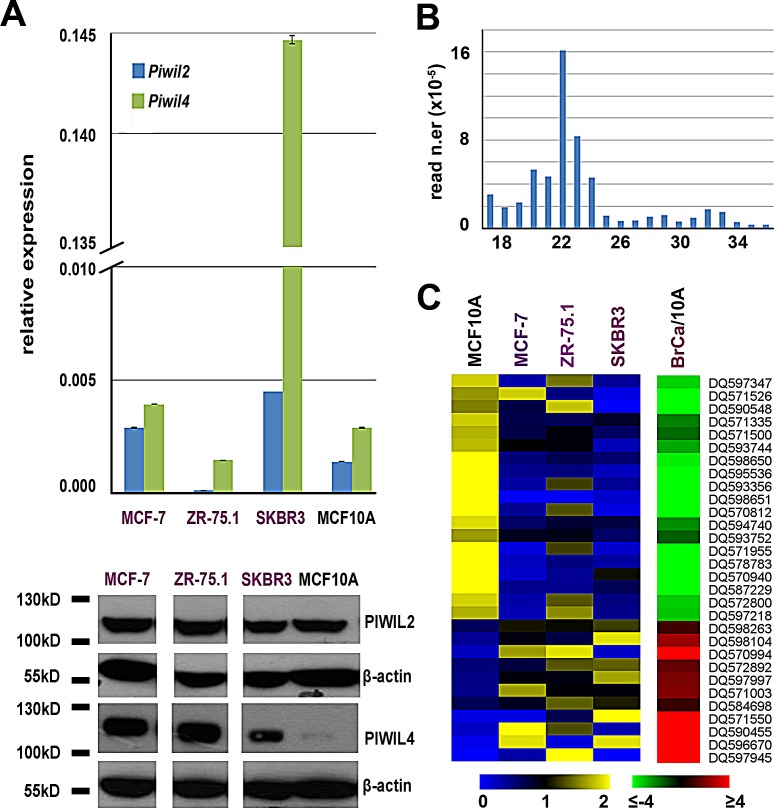
Expression of PIWIL proteins and PIWI-interacting RNAs (piRNAs) in BC cell lines A. Relative abundance of *Piwil2* and *Piwil4* mRNAs, respect to α-tubulin mRNA, by real-time quantitative *rt*PCR (*top*) and of PIWIL2 and PIWIL 4 proteins by Western blot (*bottom*) in three breast cancer cell lines and in mammary epithelial MCF10A cells. Oligonucleotide sequences and uncropped images can be found in Supplementary Table S1A and Fig. S1. B. Length distribution of unique reads in a representative small RNA sequencing library. C. Visualization of mean-centered and normalized data relative to piRNAs differentially expressed in three breast cancer cell lines respect to mammary epithelial MCF10A cells, with average fold-changes (p-value ≤0.05, Fisher's exact test) shown in the green-red heatmap to the *right*.

The human PIWI subfamily of Argonaute proteins comprise 4 members, PIWIL1/HIWI, PIWIL2/HILI, PIWIL3 and PIWIL4/HIWI2 [11], all found in testis, although recently a number of reports have identified elevated expression of HIWI and HILI in a variety of human cancers [12]. We measured expression of the genes encoding these proteins by real-time quantitative *rt*PCR and western blotting in MCF-7, ZR-75.1 and SKBR3 BC cells and in mammary epithelial MCF10A cells (Fig. 1A). Results showed that Piwil2 and Piwil4 gene mRNAs are expressed in all cell lines analyzed, with the latter being present at a very high level in SKBR3 cells (upper panel of Fig. 1A), while transcripts from Piwil1 and Piwil3 genes were undetectable. Analysis of the corresponding proteins by WB confirmed this result and revealed that PIWIL4 is present in a very low amount in MCF10A cells (lower panel of Fig. 1A). Presence of these key components of the piRNA pathway suggests that this may be active in mammary epithelial cells, and for this reason small RNA sequencing libraries were prepared from each line and sequenced, obtaining up to 24 million high-quality reads/sample (Supplementary Table S2). Read length distribution showed good uniformity among the datasets, with a major class of sequences within the 18-24nt peak, the canonical length of miRNAs, and additional reads falling in the 24–32nt interval, consistent with the piRNA size (Fig. 1B). Matching the sequence data obtained with that of known germline piRNAs present in piRNABank [13], we identified 78, 75, 117 and 138 such RNAs in MCF-7, SKBR3, ZR-75.1 and MCF10A, respectively (Supplementary Tables S2 and S3A-D). These breast piRNAs derive mainly from unique genomic loci (>80% of piRNA reads), ~72% mapping within intragenic regions (represented by either mRNA- or snoRNA-coding loci, Supplementary Table S3E), a significantly higher fraction compared to that of germline piRNAs annotated in piRNABank (32.81%, Supplementary Table S3F). The piRNAs identified show a strong preference for uridine at the 5^th^ position and lack any adenine bias at nucleotide 10, suggesting that their synthesis is likely to occur through the primary piRNA biosynthetic pathway, as previously shown in other somatic tissues [14]. Comparative analysis of piRNA expression between the three BC cell lines and the non-tumorigenic MCF10A cells showed significant differences as cluster analysis revealed a higher similarity between the three BC cells lines respect to MCF10A and, within the former, of the two hormone-responsive, ERα+ cell lines respect to ERα- SKBR3 (data not shown). Indeed, 30 piRNAs resulted differentially expressed (p-value ≤0.05 and |fold change (FC)| ≥1.5) in BC compared to MCF10A cells, 19 overexpressed and 11 underexpressed, among which DQ597945 and DQ570994 with very high and DQ598651 very low levels specifically in MCF-7 and ZR-75.1 cells (Fig. 1C, Supplementary Table S3G). Noteworthy, among those discriminating BC from MCF10A cells, most piRNAs mapping to unique genomic locations (14/17) are intragenic, including 6 in mRNA-coding, 3 in snoRNA and 5 in snoRNA embedded in mRNA-coding genes (Supplementary Table S3H).

### Effects of growth inhibition by estrogen deprivation and ERβ on piRNA expression in hormone-responsive BC cells

**Figure 2:**
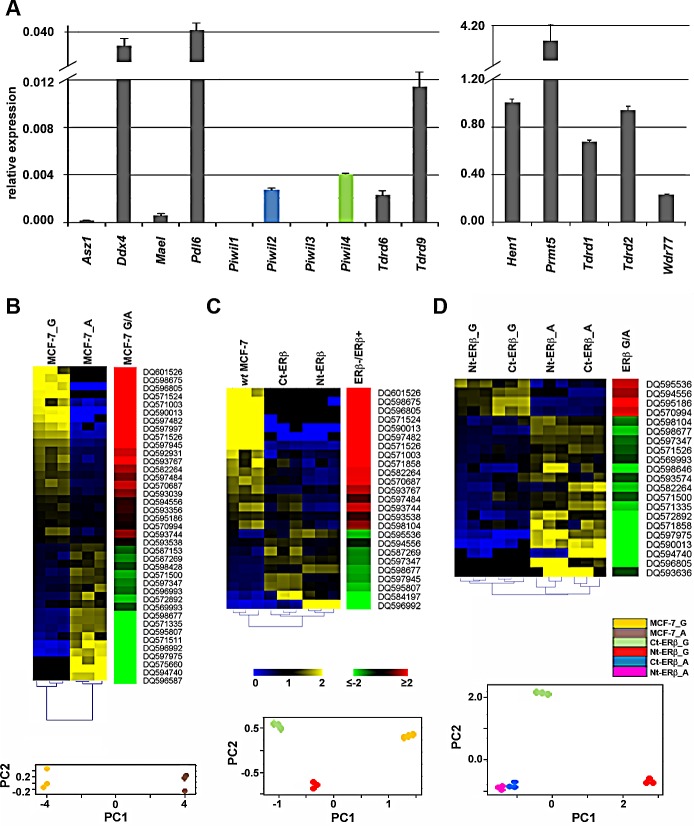
Expression of mRNAs encoding key components of the Piwi/piRNA pathway in MCF-7 cells and modulation of piRNA expression by cell growth and presence of Estrogen Receptor β A. Relative abundance, respect to α-tubulin mRNA, of transcripts encoding known components of the piRNA biogenesis pathway in *wt*-MCF7 cells by real-time quantitative *rt*PCR. Heatmaps showing mean-centered and normalized data relative to piRNAs differentially expressed in exponentially growing (G) *vs* growth arrested (A) *wt* MCF7 cells (panel B) in Ct- and Nt-ERβ+ *vs wt* (ERβ-) MCF-7 cells (panel C) or in exponentially growing *vs* growth arrested ERβ+ cells (panel D). In all cases, differentially expressed piRNAs identified with a non-parametric Wilcoxon Mann-Whitney test (α <0.05) are shown to the *left* with hierarchical clustering of replicates by Euclidean distance, together with average fold-changes (Pval ≤0.05, Fisher's exact test) in the green-red heatmaps to the *right* and the relative principal component analysis (PCA) plots at the *bottom*.

Real-time quantitative *rt*PCR profiling of mRNAs encoding proteins that play a role in piRNA biogenesis showed that many are expressed in MCF7 cells, including Ddx4, Mael, Pdl6, Piwil2, Piwil4, Hen1, all main players of the pathway, together with Asz1/Gasz, Prmt5 and its cofactor Wdr77, Tdrd1, Tdrd2/Tdrkh, Tdrd6 and Tdrd9, all encoding TUDOR domain-containing proteins involved in the pathway (Fig. 2A). This result, combined with that relative to PIWI proteins (Fig. 1A), indicates that all key components of the piRNA biogenesis and effector pathways identified in germline cells are present also in BC cells, a conclusion confirmed also for BT-549, HS578T, MDA-MB-231 and T47D BC cells, considering expression data from the NCI-60 [15] dataset (Supplementary Table S1B).

To investigate whether activity in BC cells of the piRNA pathway can be affected by changes of cell functional status, such as estrogen signaling and/or cell cycle progression, we compared the expression pattern of these RNAs in hormone-responsive MCF-7 cells before and after estrogen deprivation, a condition well known to induce growth inhibition by early G1 cell cycle arrest [16]. Small RNA libraries from exponentially growing (MCF-7_G) or estrogen-starved (MCF-7_A) cells were sequenced and reads corresponding to piRNAs were identified and quantified (Supplementary Tables S2 and S4A-C). Non-parametric Wilcoxon Mann-Whitney test was then used to evaluate differences between the two experimental groups, leading to the identification of 39 piRNAs whose expression changes in exponentially growing (G) compared to quiescent (A) cells (Fig. 2B), most (n=24) deriving from intragenic genomic regions (Supplementary Table S4F). Principal Component Analysis (PCA) confirmed the existence of clearly distinct piRNA expression patterns in the two experimental conditions (Fig. 2B, lower panel). Among the piRNAs differentially expressed, 28 showed |FC| ≥1.5 between the two culture conditions tested, including 15 up-regulated and 13 down-regulated in growing cells (Fig. 2B; Supplementary Table S4G). Notably, strong up-regulation of DQ590013 (47x), DQ596805 (58x), DQ597482 (141x), DQ598675 (359x) and DQ571524 (421x) and down-regulation of DQ596992 (−9.08x) was observed in growing cells (Supplementary Table S4G). These results, in agreement with recent results showing changes in piRNA expression during regenerative proliferation of the liver [9], demonstrate that the BC cell piRNome identified here can be modulated by mitogenic stimuli such as estrogen hormones, suggesting that it may respond to regulatory signals and/or functional cell changes, a known characteristic of miRNA and other regulatory sncRNAs.

In order to investigate this possibility, we focused on estrogen receptor β (ERβ), the oncosuppressive ER subtype that in BC interferes with the cellular effects of the oncogenic ERα also on miRNAs [10, 17, 18]. To this end, two MCF-7 cell clones stably expressing full length human ERβ (Ct- and Nt-ERβ cells), that represent a useful model to recapitulate *in vitro* the biological effects of this receptor subtype in hormone-responsive BC [10, 17-19], were used to perform smallRNA-Seq and compare piRNA expression with that of ERβ- *wt* MCF-7 cells under the same experimental conditions. Cluster analysis showed that the presence of ERβ indeed affects the BC cell piRNome, as in the two ERβ+ clones this was found clearly different from that of *wt* MCF-7 cells (Fig. 2C and Supplementary Tables S4B, S4D and S4E). Indeed, PCA showed that, according to this parameter, the two ERβ-expressing clones are clearly different from ERβ- cells (lower panel of Fig. 2C). Statistical analysis identified 25 piRNAs showing significant differences in expression (|FC| ≥1.5) between ERβ- and ERβ+ cells, 16 induced and 9 repressed by the presence of this ER subtype (Supplementary Table S4H). Interestingly, several piRNAs displayed similar changes (up- or down-regulation) following growth inhibition of MCF-7 cells by either estrogen deprivation or ERβ expression including, for example, DQ590013, DQ596805, DQ597482, DQ598675 and DQ571524 (compare Fig.s 2B and 2C and Supplementary Tables S4G and S4H). Considering the growth-inhibitory actions of ERβ in MCF-7 cells [10, 17], these results are in agreement with the recent observation that piRNA expression can be strongly influenced by cell cycle progression in somatic cells [9]. On the other hand, other piRNAs responded specifically to ERβ as, for example, DQ597945 shows an opposite behavior in the two experimental conditions and DQ571858, DQ598104, DQ595536 and DQ584197 were influenced only by ERβ. Specific effects of ERβ were confirmed when cells expressing this receptor subtype were analyzed in the presence and absence of estrogen as, among the 21 piRNAs that responded in this case, DQ595186, DQ594556, DQ570994, DQ598646 and DQ571858 showed differential expression exclusively upon growth arrest of ERβ+ cells (Fig. 2D and Supplementary Table S4I-K), while were unaffected under the same conditions in *wt* MCF-7 cells (Supplementary Table S4G).

Taken together, these results indicate that the BC cell piRNome responds to cell transformation as well as to growth inhibition and/or cell cycle phasing, as well as to hormonal signals and transcription factors.

### Identification of piRNAs differentially expressed in tumors compared to normal breast tissues and their target mRNAs

**Figure 3:**
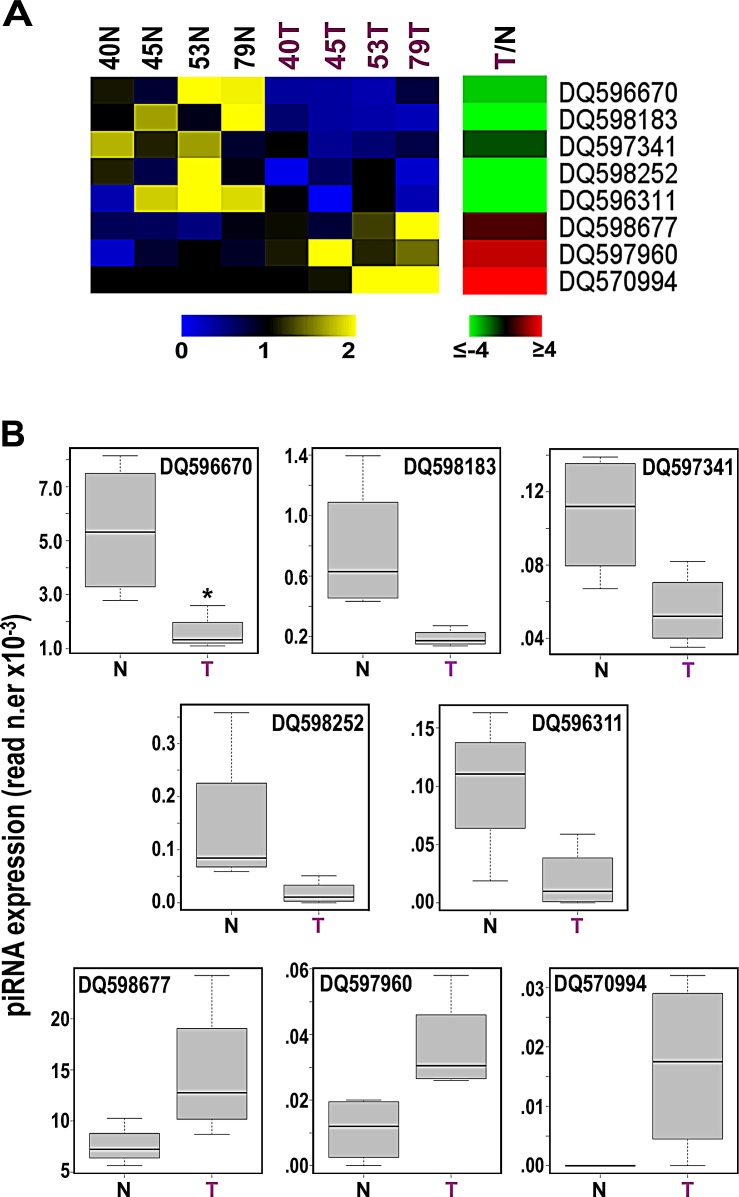
Identification of 8 piRNAs differently expressed in cancer vs normal breast tissues A, Heatmaps showing mean-centered and normalized data relative to piRNAs differentially expressed in cancerous (T) *vs* non-tumoral (N) breast tissue samples from the same patients, identified with a non-parametric Wilcoxon Mann-Whitney test (α <0.05), with average fold-changes (p-value ≤0.05, Fisher's exact test) shown in the green-red heatmap to the *right*. B, Boxplots summarizing differences in expression of the same piRNAs between tumor and matched non-tumor samples. Samples 40, 45, 53 and 79 correspond to patients TAX577740, TAX577745, TAX577453, and TAX577579, respectively.

Searching for evidence that the expression of germline piRNAs observed in cell lines *in vitro* occurs also in BC *in vivo*, we analyzed a well characterized sncRNA-Seq dataset from 4 paired samples of normal and cancer (invasive ductal carcinoma) breast tissues from the same patients [20; GEO Acc. N.er GSE39162]. Interestingly, we could identify ~150 expressed piRNAs/sample (Supplementary Table S5A) in BC tissues, including the vast majority of those found expressed in common with cell lines displayed above (see Supplementary Tables S3A-D). Hierarchical clustering and PCA analysis based on miRNAs and piRNAs led to different results for the two classes of sncRNAs, with the global expression pattern of miRNAs clearly distinguishing tumors from normal tissue samples (Supplementary Fig. S2A), while that of piRNAs being unable to do so (Supplementary Fig. S2B). This result, that could be related to the different complexities of the two datasets including 831 miRNAs but only 146 piRNAs, led us to consider the possibility that only a limited number of piRNAs are differentially expressed in the two tissues. Indeed, while at least 68 miRNAs are differentially expressed in tumor samples [20 and data not shown], stringent statistical analysis (Wilcoxon Mann-Whitney test) identified a tumor-specific pattern comprising 8 piRNAs (α <0.05; Fig. 3A and Supplementary Table S5B). Considering the mean read counts within each group of samples and |FC| ≥1.5 with p-value ≤0.05, DQ596670, DQ598183, DQ597341, DQ598252 and DQ596311 were found underexpressed and DQ598677, DQ597960 and DQ570994 overexpressed in BC tissues compared to their normal counterparts (Fig. 3B and Supplementary Table S5B). Interestingly, comparing the results obtained in cell lines and biopsies none of the 8 piRNAs showing differential expression in breast tumor tissues was similarly influenced by growth stimulation *in vitro*, suggesting that the differences detected here are not directly related to the higher proliferation rate of cancer cells respect to their normal counterparts. On the other hand, DQ570994 was expressed at higher levels both in BC cells and tissues (see Fig.s 1C and 3).

**Figure 4:**
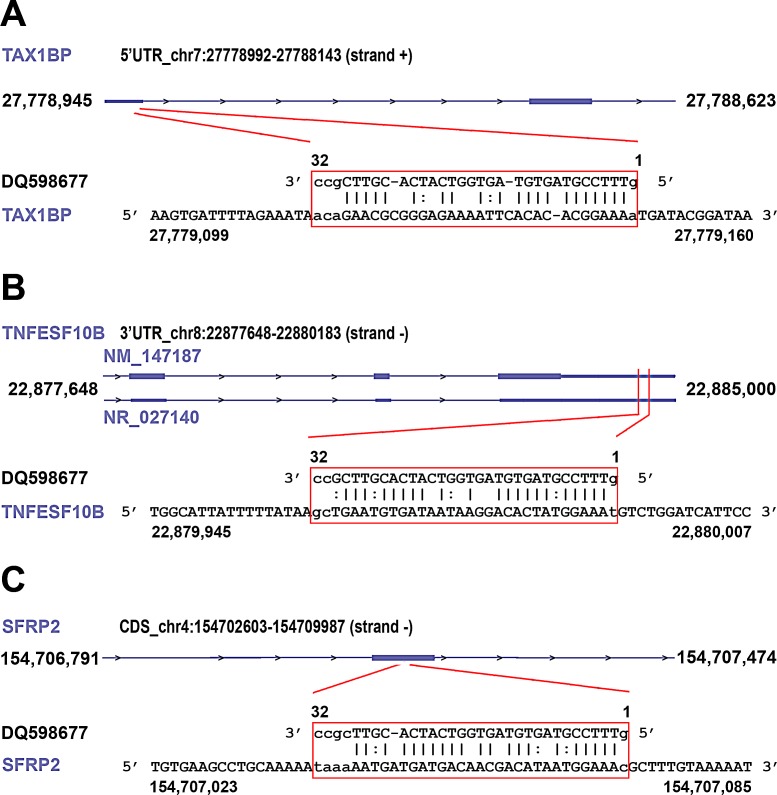
Examples of piRNA-RNA sequence complementarity exploited to identify transcripts representing putative targets of the piRNAs found differently expressed in cancer vs normal breast tissue miRanda was used to identify breast cancer RNAs showing significant sequence complementarity with the eight piRNAs showing significant differences in expression between cancer and matched normal breast tissues. Analysis was performed applying both Watson-Crick ( | ) or GU wobble (: ) base pairing, stringent alignment scores (≥170) and a high binding energy threshold (≤-20.0 kcal/mol). As an example, matches (in red box) for piRNA DQ598677 in the 5′ UTR of TAX1BP mRNA (A), in the 3′ UTR of TNFESF10B mRNA (NM_147187) and in the long non-coding RNA NR_027140 encoded by the same locus (B) and in the coding region of SFRP2 mRNA (C), respectively, are shown. For details, see Supplementary Table S6G.

**Figure 5:**
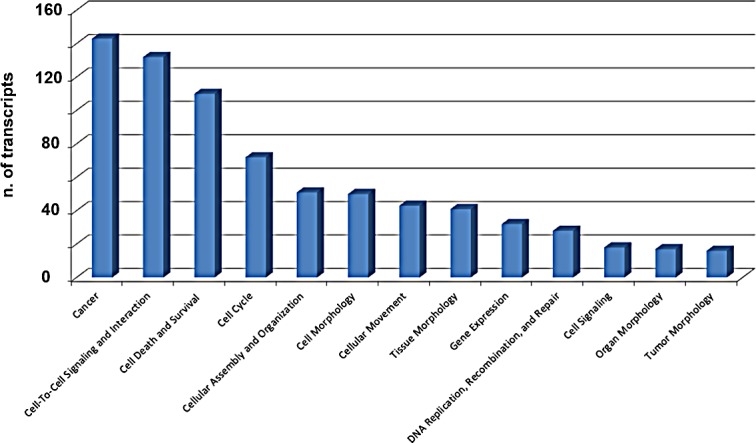
Functional annotation analysis of biological processes involving mRNAs targeted by 8 piRNAs differentially expressed in cancer vs matched normal breast tissue biopsies Ingenuity Pathway Analysis (IPA) was used to identify biological processes significantly associated (p-value ≤0.05) with mRNAs targeted by the eight piRNAs found differentially expressed in neoplastic *vs* normal breast tissue samples from the same patients.

Recent evidences indicate that piRNAs can form specific RNA silencing complexes (pi-RISC) capable of promoting RNA repression *via* imperfect base-pairing between the two RNAs, by a mechanism that closely resembles that of miRNAs [21, 22]. We thus searched within the BC transcriptome all RNAs that, according to this mechanism, may represent potential targets of the 8 piRNAs differentially expressed in cancer tissues. As described in Methods, applying stringent thermodynamic parameters and binding energy thresholds to predict biologically relevant RNA-RNA interactions each piRNA analyzed was found complementary to a number of RNAs ranging from 23 to 383 (Supplementary Tables S6A-I) and including not only mRNAs but also pseudogene transcripts and long ncRNAs (Supplementary Tables S6B-I). Representative examples of piRNA:RNA complementarities identified by this approach are shown in Fig. 4. Interestingly, many of the proteins encoded by these putative piRNA-target mRNAs are involved in key cellular processes in BC, including cell-to-cell signaling and interaction, cell death and survival, cell cycle, DNA replication and repair (Fig. 5 and Supplementary Table S7), suggesting that, in analogy with miRNAs, piRNAs represent a new class of master regulators in cancer cells.

## DISCUSSION

piRNAs are abundant and extensively studied in germline and gonads, but little is known about their role in somatic cells. In complex with PIWI proteins, they are known to constitute a conserved RNA silencing pathway that is related, although distinct in its mechanism and functions, to other sncRNA-mediated silencing pathways [4]. Recent results suggest that PIWI proteins and piRNAs can be found also in cancer [review in 2, 8, 23], but the relationships between these and tumor cell biology remain poorly understood. We report here that Piwil2 and Piwil4 and other genes encoding known components of the piRNA biogenesis and effector pathways are expressed in human BC cells and that a specific set of piRNAs is expressed, some at a very high level, in normal and transformed mammary cell lines *in vitro* and tissues *in vivo*, demonstrating the presence of an active piRNA pathway in these cancers. Interestingly, ~40% of the BC piRNAs derive from intragenic regions of the genome within protein coding and/or snoRNA genes, suggesting that the corresponding transcripts might represent precursors of piRNAs that, for this reason, would in turn be subject to the same regulatory signals that control transcription of their carrier genes. Indeed, a significant fraction of these piRNAs respond to estrogen, as their intracellular level changes upon hormone deprivation, as well as to ERβ, a transcription factor controlling proliferation and other key functions in BC cells [10, 17]. It is possible to assume that, in analogy with what has been demonstrated for miRNAs [10, 18], ERs and other regulatory factors directly modulate the expression of these small RNAs in specific tumor types, and/or in response to intra- or extra-cellular stimuli in a given cancer. Indeed, expression of at least three piRNAs (DQ597945, DQ570994 and DQ598651) was found significantly different in ER+ MCF-7 and ZR-75.1 cells, compared to ER- SKBR3. Based on these evidences, piRNAs represent a new tool to investigate the molecular mechanism(s) of estrogen action in hormone-responsive BC, including those determining the less aggressive clinical phenotype of ERβ+ tumors [10]. This is further supported by the result demonstrating that mRNAs representing putative targets for post-transcriptional regulation by the 8 piRNAs found deregulated in BC biopsies encode also proteins involved in key cancer cell functions (Fig. 5 and Table S7).

In contrast to the high number detectable in germline cells, the piRNA population identified in BC is relatively small, representing <1% of the sncRNA molecules detected. It is possible, however, that BC expresses a specific set of these molecules that is not yet known, and for this reason escapes the current methods for RNA-Seq data analysis based on sequence alignment. The results described here provide a sound rationale to justify a systematic search of such ‘somatic’ cancer piRNAs and their intracellular targets, applying the biochemical and genetic methods the led to the initial discovery of these sncRNAs in germline cells.

## METHODS

### Cell culture, stable transfections and immunoblotting

MCF-7 (ATCC HTB-22), SKBR3 (ATCC HTB-30) and ZR-75.1 (ATCC CRL-1500) cell lines were maintained in Dulbecco's modified Eagle medium (DMEM; Sigma-Aldrich, Milan, Italy) supplemented with 10% fetal bovine serum (FBS) (HyClone, Cramlington, UK) and antibiotics: 100U/ml penicillin, 100mg/ml streptomycin, 250ng/ml Amfotericin-B (exponential growing condition). MCF10A (ATCC CRL-10317) mammary epithelial cells were maintained in MEGM Bullet Kit (Lonza, Milan, Italy) supplemented with 100 ng/ml cholera toxin (Sigma-Aldrich, Milan, Italy). Steroid deprivation (starvation) was performed by culturing cells in phenol red-free DMEM and 5% Dextran-Coated Charcoal stripped serum (DCC-FBS) for 5 days, as described earlier [24]. PIWIL proteins expression was analyzed by sodium dodecyl sulphate (SDS) acrylamide gel electrophoresis and immunoblotting of total protein extracts, using rabbit anti-PIWIL1 (ab85125, Abcam, Cambrige, UK), rabbit anti-PIWIL2 (ab26408, Abcam), mouse anti-PIWIL3 (ab77088, Abcam), rabbit anti-PIWIL4 (ab111714, Abcam) and mouse anti-β-actin (A1978, Sigma Aldrich).

### RNA Purification

Total RNA was extracted from cell lines using the standard RNA extraction method with TRIzol (Invitrogen, Carlsbad, CA, USA), quantitated with NanoDrop-1000 spectrophotometer (Thermo Fisher Scientific, Cinisello Balsamo, Italy) before integrity assessment with an Agilent 2100 Bioanalyzer (Agilent Technologies, Santa Clara, CA, USA).

### Real-time quantitative rtPCR

2μg of total RNA was retro transcribed with AffinityScript cDNA Synthesis Kit (Agilent Technologies, Milano, Italy) following the manufacturer's instructions. Specific primer sets (Supplementary Table S1A) were designed with Primer3 (http://www.broad.mit.edu/cgi-bin/primer/primer3) to amplify 100–200bp products. cDNAs were diluted to a final concentration of 20 ng per reaction. Real-time quantitative *rt*PCR was performed in triplicate using Brilliant II SYBR® Master Mixes (Agilent Technologies) on Mx3005P™ Real-Time PCR System (Agilent Technologies) and expression values were normalized against α-tubulin mRNA.

### Small RNA sequencing

For RNA-seq, 1μg of total RNA/cell lines was used for library preparation with Illumina TruSeq small RNA sample preparation Kit. Three technical replicates of each library (10pM) were sequenced on HiSeq1500 (Illumina) for 50 cycles. Small RNA sequencing data were analyzed using iMir [25]. Raw small RNA sequencing data are available in NCBI Gene Expression Omnibus (GEO) database (http://www.ncbi.nlm.nih.gov/gds/) with Accession Number GSE56134.

### Bioinformatic analyses

NCI-60 Analysis Tool from CellMiner [26] was used to identify expression level of transcripts encoding known components of the piRNA biogenesis pathway in BC cell lines included in the NCI-60 dataset.iMir [25] was used to identify the sncRNA families studied, i.e. miRNAs ( miRBase v19), tRNAs or rRNAs (Human genome assembly, GRCh37/hg19) and germline piRNAs [piRNABank; 13] with Minimum Read Count of 3, Minimum Read Length of 17, minReadlengthTrans of 32 and maxReadLength of 32. Quantile normalization and Fisher's exact test were used to analyze the data from BC cell lines compared to the mammary epithelial MCF10A cells [27], while data from human biopsies were normalized according to miRNA sequencing data analysis methods (www.arraystar.com). The expression of piRNAs was calculated for the different groups of samples by the Wilcoxon Mann-Whitney test at α <0.05. Biclustering and Principal Component Analysis (PCA) of sncRNA expression profiles and piRNA differential expression analyses were performed with DESeq (*v* 1.14.0) bioconductor package [28]. piRNAs were considered differentially expressed when showing absolute fold-change (*treatment/control)* ≥1.5 with p-value ≤0.05, as determined by Fisher's exact test. IntersectBed (Bedtools) [29] was used to identify genomic piRNA loci within repeat regions (RepeatMasker, Human genome assembly, GRCh37/hg19), snoRNAs (Human genome assembly, GRCh37/hg19) and inter-/intra-genic regions (Human genome assembly, GRCh37/hg19) of the human genome.

piRNA target RNAs were identified by sequence complementarity between each piRNA and the 5′-UTRs, CDSs or 3′-UTRs of all known human RNAs (RefSeq gene annotations, Human genome assembly, GRCh37/hg19), or RNAs expresses in breast invasive carcinomas (The Cancer Genome Atlas, http://cancergenome.nih.gov/), with miRanda [v3.3a; 30], an algorithm for detection of potential small RNA binding sites in RNAs, applying stringent alignment score (sc; ≥170) and energy threshold (en; ≤-20.0 kcal/mol). Functional analyses were performed with the Ingenuity Pathway Analysis suit (Ingenuity Systems, USA) to identify biological processes significantly associated (p-value ≤0.05) to piRNA-targeted mRNAs.

## SUPPLEMENTARY FIGURES AND TABLES




