# Supplementary material for: RNA sequencing identifies specific PIWI-interacting small non-coding RNA expression patterns in breast cancer
Source: Oncotarget. 2014 Sep 16;5(20):9901–10. doi: 10.18632/oncotarget.2476 (PMC4259446; doi:10.18632/oncotarget.2476)
Supplement: Supplementary file 1 [file oncotarget-05-9901-s001.pdf]

RNA sequencing identifies specific PIWI-interacting small non-coding RNA expression patterns in breast cancer

Supplementary Material

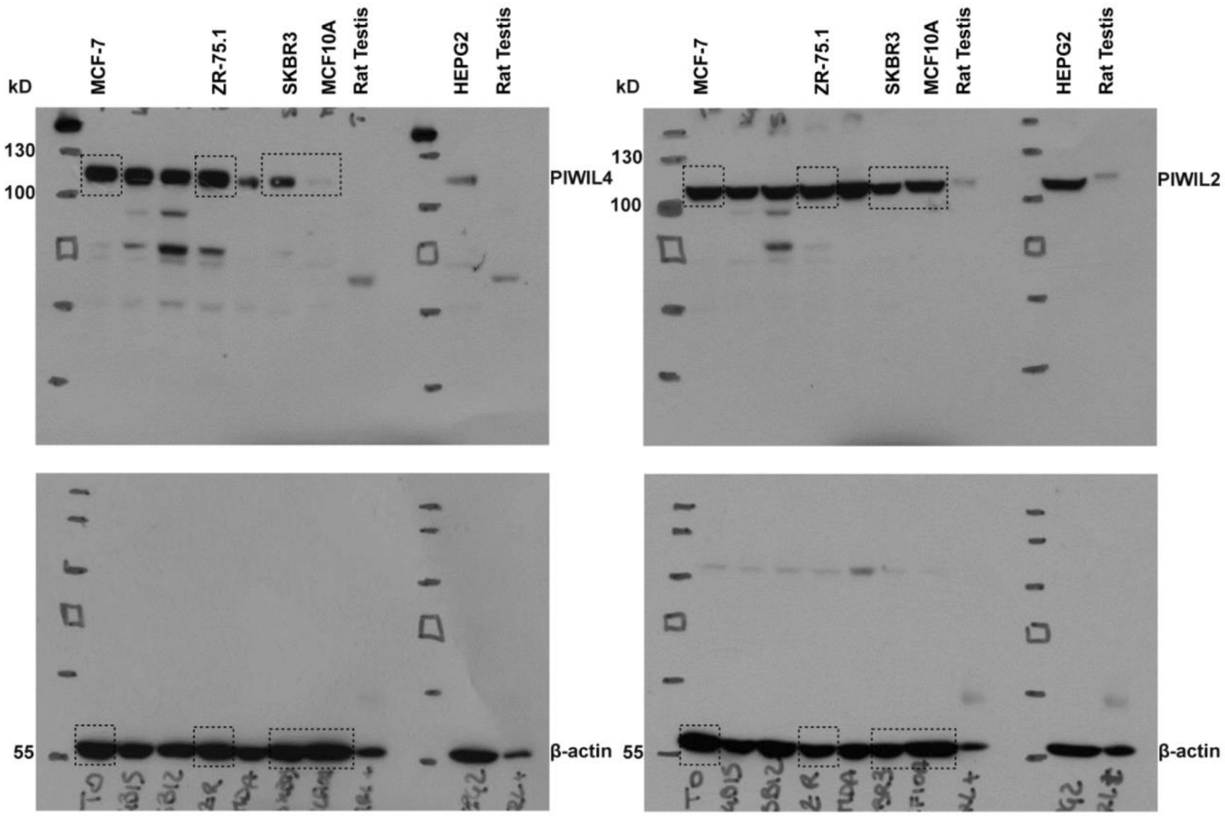

Supplementary Figure S1: Full length uncropped images of Western blot images from Fig 1A. Dotted boxes mark the border of the final cropped image for each individual protein. As a positive control for primary antibody, HEPG2 (anti-PIWIL2 and anti-PIWIL4) and rat testis lysates (anti-PIWIL2) were used.

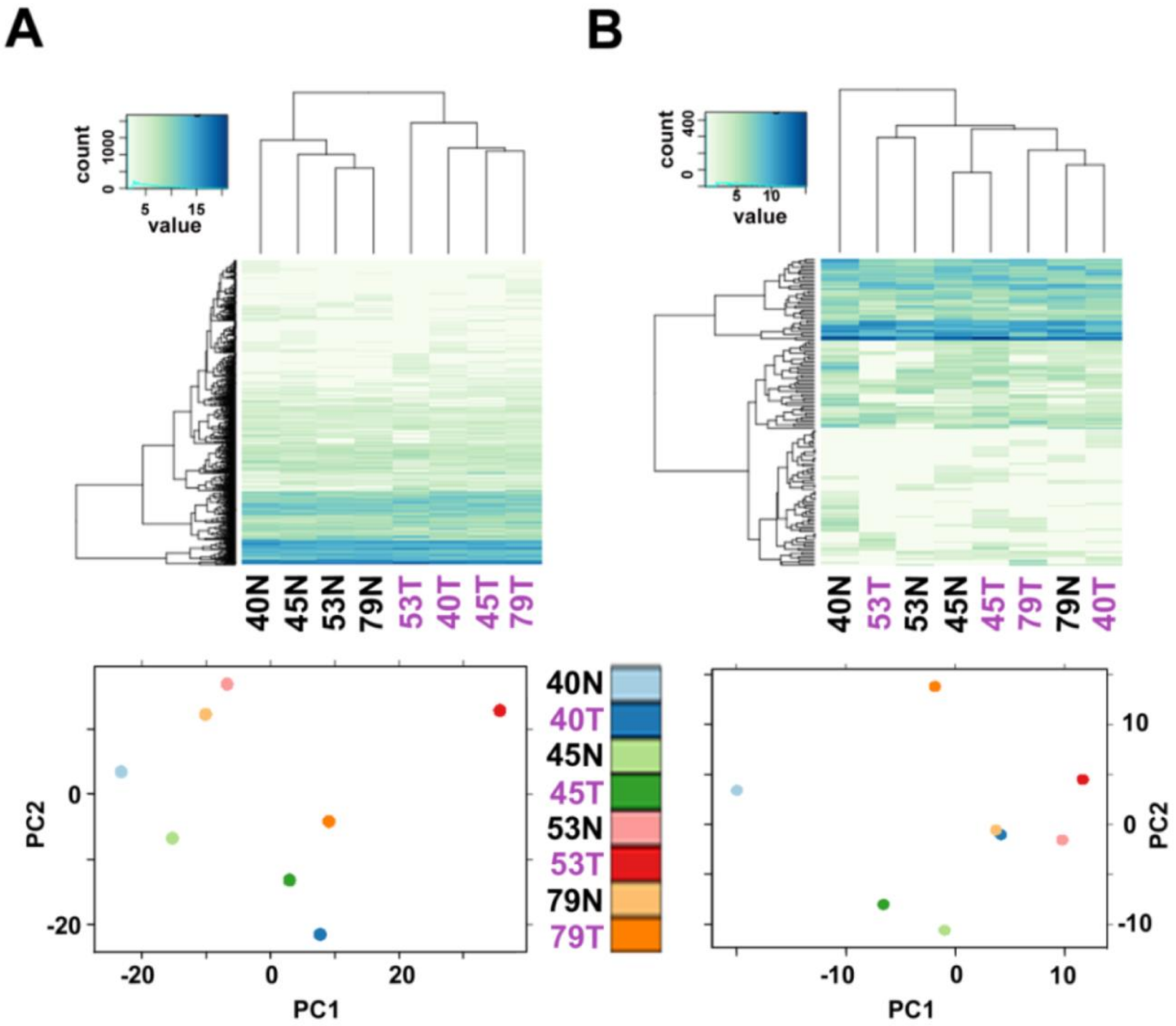

Supplementary Figure S2: miRNA (A) and piRNA (B) expression profiles in paired normal and tumor breast tissue samples. *Top*: samples (cancer: T; normal: N) and sncRNAs (miRNA and piRNA) were clustered calculating the respective Euclidean distances from VST (variance-stabilizing transformation)-normalized read counts. *Bottom*: Principal Component Analysis showing distances between samples plotted according to two principal components (PC1 and 2). Samples 40, 45, 53 and 79 correspond to patients TAX577740, TAX577745, TAX577453, and TAX577579, respectively.
