# Supplementary material for: RNA sequencing identifies specific PIWI-interacting small non-coding RNA expression patterns in breast cancer
Source: Oncotarget. 2014 Sep 16;5(20):9901–10. doi: 10.18632/oncotarget.2476 (PMC4259446; doi:10.18632/oncotarget.2476)
Supplement: Supplementary file 2 [file oncotarget-05-9901-s002.pdf]

RNA sequencing identifies specific PIWI-interacting small non-coding RNA expression patterns in breast cancer

A. Hashim *et al.*

Supplementary Tables S1-S7

Supplementary Table S1A: Oligonucleotides used for quantitative real-time rtPCR analysis of piRNA pathway gene expression. For each oligonucleotide primer, sequence and accession number are reported.

**Gene**	**Oligo Name**	**Sequence**	**Acc. N.er**
Asz1	ASZ1_QF	CTGCTCGAGATGGTCACACC	NM_130768.2
	ASZ1_QR	TGCCCACGTTAAAGCAGTGT	
Ddx4	DDX4_QF	GTTTTCCAAGAGAGGCGGCTA	NM_024415.2
	DDX4_QR	TAGACCAAATCCTCCACGGC	
Hen1	HEN1_QF	AAGGTTGCAGACCTGGGATG	NM_144584.2
	HEN1_QR	ACGAATCCCCTCTCCATCGT	
Mael	MAEL1_QF	CATTGTCAGGCTGCAAGTGA	NM_032858.1
	MAEL1_QR	CAGTTCCCTGGGTTGGGATG	
Pdl6	PDL6_QF	CTGCGCAAGGCAGGGATCCAG	NM_178836.3
	PDL6_QR	GCGAGCCAGTGATGAGCACCC	
Piwil1	PIWIL1_QF	ACGAAGTGCCACAGTTTTTGG	NM_004764.4
	PIWIL1_QR	AGTCTTCCTCCAGACTGAGC	
Piwil2	PIWIL2_QF	GCCTGGGTTGAACTAAAGGA	NM_018068.3
	PIWIL2_QR	CCATGATGATGCAAACAACC	
Piwil3	PIWIL3_QF	TCAGATGGCAGCAAAATCAC	NM_001255975.1
	PIWIL3_QR	ACGTTGTGTACCCGTTAGGC	
Piwil4	PIWIL4_QF	ATGGCACCGAGATCACCTAT	NM_152431.2
	PIWIL4_QR	GCTGAGCCTCACTGTTGTCA	
Prmt5	PRMT5_QF	GGTCTTCCAGCTTTCCTGCT	NM_006109.3
	PRMT5_QR	TACCCGCATCCAGAACATGG	
Tdrd1	TDRD1_QF	AAATCCGAGACGCCTCATGT	NM_198795.1
	TDRD1_QR	ACCCAAGGGAACACTGGTTT	
Tdrd2	TDRD2_QF	CGGACTTCTTGGACAAGCCT	NM_001083965
	TDRD2_QR	AATGTCAGCCGCTCTTCTCT	
Tdrd6	TDRD6_QF	GAGTCCACCTGAGAAAAGGGG	NM_001010870.2
	TDRD6_QR	TTGGTCTGCGTAAGATGCCA	
Tdrd9	TDRD9_QF	TGCGTTCGAAAGGTCACTCA	NM_153046.2
	TDRD9_QR	AAAGATGGCCTTGGACCTGG	
Tub1a	TUB_QF	GACCGCCTAAGAGTCGCGCTG	NM_006009.3
	TUB_QR	GCACTCACGCATGGTTGCTGCT	
Wdr77	WDR77_QF	CGGCTCCCTCTGGCTTTTTA	NM_024102.2
	WDR77_QR	AACAGCACCTGAATCGGAGG	

Supplementary Table S1B: Data relative to expression of transcripts encoding known components of the piRNA biogenesis pathway for additional breast cancer cell lines derived from NCI-60 dataset. For each given transcript, the total numbers of probes of the microarray that scored positive upon hybridization and of those that passed quality control are reported, together with $\log_2$ values of minimum, maximum and average fluorescence intensity detected, and corresponding average Z score.

			Transcript probe log2 intensities		Average transcript probe log2 intensities					Average transcript intensity Z scores				
**Transcript**	**Total probes found**	**Probes that passed quality control**	**Minimum**	**Maximum**	**MCF7**	**MDA_MB_231**	**HS578T**	**BT_549**	**T47D**	**MCF7**	**MDA_MB_231**	**HS578T**	**BT_549**	**T47D**
Asz1	20	3	2.41	4.19	2.82	2.76	3.32	2.65	3.01	-0.55	-0.84	0.73	-1.09	-0.16
Ddx1	35	6	2.09	3.54	2.63	2.48	2.51	2.48	2.73	0.11	-0.38	-0.36	-0.48	0.46
Mael	40	8	2.46	5.95	3.13	5.95	3.16	3.05	3.06	-0.28	4.84	-0.28	-0.43	-0.33
Piwil1	163	52	3.29	8.74	4.04	3.91	3.98	3.96	3.91	-0.15	-0.28	-0.17	-0.23	-0.28
Piwil2	51	8	3.38	5.72	3.91	4.22	4.24	4.17	3.98	-0.81	-0.12	-0.11	-0.18	-0.53
Piwil3	44	6	3.01	4.53	3.95	3.71	3.49	3.76	3.83	0.83	0.16	-0.63	0.2	0.48
Piwil4	70	29	2.96	8.79	3.55	3.95	3.5	3.69	3.42	-0.5	-0.13	-0.54	-0.36	-0.63
Prmt5	37	27	8.41	10.67	9.83	9.82	8.94	10	9.27	0.31	0.27	-1.47	0.65	-0.76
Tdrd1	61	43	2.66	8.21	6.96	3.12	3.16	3.26	3.38	3.94	-0.29	-0.25	-0.12	0.01
Tdrd6	42	7	2.14	3.85	2.75	2.89	2.75	2.61	2.78	-0.16	0.21	-0.23	-0.63	-0.15
Tdrd9	66	46	2.87	7.45	3.44	3.39	3.51	3.38	3.44	-0.22	-0.34	-0.17	-0.37	-0.27
Wdr77	51	18	8.8	10.5	9.53	10.23	9.05	9.95	9.14	-0.19	1.55	-1.35	0.88	-1.14

Supplementary Table S2: Overview of small RNA sequencing libraries generated for this study. For each library, number of reads before and after adapter cleavage and reads mapped in each category of sncRNA are shown.

Cell line	Raw read count	Filtered read count	Annotation categories							
			miRNAs		piRNAs		tRNAs		rRNAs	
			N.er	Read count	N.er	Read count	N.er	Read count	N.er	Read count
MCF10A	25,018,240	23,988,675	738	12,318,726	138	185,475	127	63,334	4	761,097
MCF-7	13,019,082	11,784,358	593	7,026,882	78	61,591	75	12,140	4	277,845
ZR-75.1	22,893,597	21,540,490	725	17,471,939	117	139,784	102	74,408	4	333,211
SKBR3	9,056,994	7,201,109	492	3,134,331	75	30,572	47	8,733	4	242,998
MCF-7_G_1	4,327,501	3,506,994	472	2,310,200	60	19,277	37	3,789	4	87,828
MCF-7_G_2	4,337,535	3,574,145	469	2,314,040	58	20,213	44	3,773	4	89,831
MCF-7_G_3	4,354,046	3,618,196	476	2,374,218	55	21,094	40	3,930	4	93,430
MCF-7_A_1	6,071,484	5,385,738	460	4,626,169	57	21,679	32	9,535	4	70,304
MCF-7_A_2	6,075,950	5,447,209	467	4,621,007	57	22,259	33	8,960	4	73,592
MCF-7_A_3	6,085,784	5,476,334	473	4,725,976	58	22,742	32	8,992	4	76,606
Ct-ERβ_G_1	2,849,255	2,476,261	420	2,037,155	48	13,087	33	3,974	4	59,213
Ct-ERβ_G_2	2,851,956	2,511,218	421	2,036,288	49	13,647	37	3,839	4	61,261
Ct-ERβ_G_3	2,860,905	2,534,085	427	2,087,819	52	14,527	35	4,034	4	63,161
Ct-ERβ_A_1	4,197,268	3,569,321	427	2,781,283	62	19,398	32	10,586	4	121,570
Ct-ERβ_A_2	4,203,850	3,624,841	436	2,786,073	62	20,036	37	10,038	4	125,795
Ct-ERβ_A_3	4,232,281	3,667,198	444	2,863,947	64	20,691	34	10,267	4	131,566
Nt-ERβ_G_1	4,364,662	3,783,073	454	3,126,539	59	19,594	43	6,671	4	117,795
Nt-ERβ_G_2	4,383,706	3,843,922	458	3,134,747	63	20,165	44	6,674	4	121,431
Nt-ERβ_G_3	4,391,850	3,870,049	457	3,204,734	61	21,304	43	6,859	4	126,843
Nt-ERβ_A_1	6,193,051	5,490,264	459	4,732,554	60	19,414	40	10,032	4	89,061
Nt-ERβ_A_2	6,197,557	5,554,537	454	4,727,273	58	19,670	46	9,697	4	91,860
Nt-ERβ_A_3	6,208,568	5,584,789	469	4,833,239	58	20,838	40	10,036	4	95,392

Supplementary Table S3A: Number of raw (not normalized) reads that mapped to each identified piRNA in the library from *wt*-MCF7 cells. piRNAs are named in accordance to NCBI accession number.

**NCBI Accession**	**Raw read count**
DQ587153	11370
DQ595186	8291
DQ598677	6688
DQ597484	3616
DQ590013	3551
DQ596805	3251
DQ594556	2377
DQ593039	2339
DQ593538	2209
DQ571526	2162
DQ597945	2151
DQ597347	1553
DQ571524	1360
DQ598675	1170
DQ570687	1057
DQ597482	1017
DQ590548	840
DQ595807	805
DQ582264	668
DQ569993	610
DQ570994	589
DQ592931	482
DQ571335	422
DQ593767	353
DQ571500	274
DQ598263	243
DQ596993	229
DQ593744	214
DQ598104	205
DQ598428	119
DQ593574	108
DQ582566	94
DQ587269	94
DQ571003	91
DQ572892	84
DQ571858	76
DQ598646	66

DQ597997	54
DQ598650	53
DQ588040	52
DQ571419	50
DQ570698	44
DQ571511	42
DQ571591	41
DQ597975	39
DQ595536	36
DQ596992	32
DQ593356	32
DQ596670	27
DQ596932	25
DQ597397	22
DQ584698	21
DQ570540	19
DQ601526	18
DQ584197	18
DQ592932	14
DQ575884	12
DQ593415	12
DQ593752	12
DQ571589	9
DQ590455	7
DQ594740	6
DQ570992	6
DQ593407	6
DQ595023	6
DQ596587	5
DQ570728	5
DQ593636	5
DQ570812	4
DQ598619	4
DQ596155	4
DQ570999	3
DQ598378	3
DQ600174	3
DQ600515	3
DQ580405	3
DQ575660	3
DQ598130	3

Supplementary Table S3B: Number of raw (not normalized) reads that mapped to each identified piRNA in the library from SKBR3 cells. piRNAs are named in accordance to NCBI accession number.

NCBI Accession	Raw read count
DQ593039	7477
DQ598677	4863
DQ587153	4002
DQ592931	1584
DQ582264	1251
DQ597484	1152
DQ595186	1107
DQ597347	985
DQ593538	968
DQ595807	680
DQ570687	664
DQ597975	590
DQ596993	377
DQ594556	324
DQ569993	318
DQ598104	281
DQ571335	279
DQ590013	277
DQ598675	249
DQ598183	236
DQ571511	209
DQ598263	200
DQ598428	197
DQ596992	167
DQ571500	162
DQ596805	151
DQ596932	147
DQ571858	144
DQ572892	127
DQ598646	120
DQ593767	108
DQ597341	97
DQ597997	95
DQ571419	80
DQ571526	79
DQ570698	68

DQ593574	68
DQ598650	60
DQ592932	57
DQ571003	47
DQ570994	43
DQ587269	41
DQ593744	34
DQ582566	31
DQ593356	30
DQ571550	27
DQ596670	24
DQ598167	22
DQ584698	22
DQ596155	21
DQ597482	19
DQ596587	18
DQ595536	17
DQ575884	14
DQ571524	13
DQ571589	13
DQ571823	13
DQ576872	12
DQ594740	11
DQ592953	10
DQ597945	10
DQ588040	9
DQ570540	8
DQ571591	8
DQ593752	7
DQ570728	7
DQ593407	6
DQ570999	6
DQ575660	6
DQ596538	5
DQ595292	4
DQ570940	4
DQ598651	4
DQ593636	3
DQ571388	3

Supplementary Table S3C: Number of raw (not normalized) reads that mapped to each identified piRNA in the library from ZR-75.1 cells. piRNAs are named in accordance to NCBI accession number.

NCBI Accession	Raw read count
DQ598677	55743
DQ595186	27297
DQ597945	17098
DQ587153	9717
DQ597347	5403
DQ594556	3445
DQ593039	2368
DQ597484	2268
DQ593538	2180
DQ595807	1974
DQ590548	1643
DQ582264	1410
DQ592931	1172
DQ597975	674
DQ569993	645
DQ571526	543
DQ570994	543
DQ596992	518
DQ597341	326
DQ571500	320
DQ571335	302
DQ596993	296
DQ593767	274
DQ598428	255
DQ587269	238
DQ598263	219
DQ593574	214
DQ593744	185
DQ570687	182
DQ571511	164
DQ572892	150
DQ598104	141
DQ593356	128
DQ582566	111
DQ593407	100
DQ598650	93
DQ571419	86

DQ588040	78
DQ598646	76
DQ584197	76
DQ597997	71
DQ571003	65
DQ592932	58
DQ571591	54
DQ597397	44
DQ595536	44
DQ598675	43
DQ584698	43
DQ570698	42
DQ590013	39
DQ595023	39
DQ571858	36
DQ593636	33
DQ596932	31
DQ597403	30
DQ570812	24
DQ596587	24
DQ570540	21
DQ593752	18
DQ597482	18
DQ575884	18
DQ594740	17
DQ571873	15
DQ593415	15
DQ601526	13
DQ571955	11
DQ575746	11
DQ577008	10
DQ570728	10
DQ571589	9
DQ570992	9
DQ598180	8
DQ600174	8
DQ590455	8
DQ597916	8
DQ581533	8
DQ571550	8
DQ576880	7
DQ577356	7

DQ597218	7
DQ596805	6
DQ590835	6
DQ598130	6
DQ585261	6
DQ600515	6
DQ572800	6
DQ594577	5
DQ597608	4
DQ596150	4
DQ570999	4
DQ594453	4
DQ593423	4
DQ598294	4
DQ596516	4
DQ598175	4
DQ571425	4
DQ571388	4
DQ573323	4
DQ571823	4
DQ575660	4
DQ590404	4
DQ590830	3
DQ598991	3
DQ596670	3
DQ575827	3
DQ596756	3
DQ573835	3
DQ578783	3
DQ587912	3
DQ598190	3
DQ582593	3
DQ594335	3
DQ570940	3
DQ585669	3
DQ592811	3
DQ598183	3
DQ596155	3

Supplementary Table S3D: Number of raw (not normalized) reads that mapped to each identified piRNA in the library from MCF10A cells. piRNAs are named in accordance to NCBI accession number.

NCBI Accession	Raw read count
DQ595186	58394
DQ598677	34585
DQ587153	29934
DQ597347	15411
DQ593039	10764
DQ597484	4798
DQ593538	3608
DQ595807	3450
DQ594556	2641
DQ571526	2569
DQ592931	2314
DQ590548	2180
DQ571335	1327
DQ582264	961
DQ571500	895
DQ570687	796
DQ596993	784
DQ569993	760
DQ590013	570
DQ593744	552
DQ593767	543
DQ598650	517
DQ598428	473
DQ595536	454
DQ593356	402
DQ587269	378
DQ593574	352
DQ596992	332
DQ571511	330
DQ597975	295
DQ598675	291
DQ598651	283
DQ598263	260
DQ582566	222
DQ588040	168
DQ598104	164
DQ570698	159
DQ597482	151
DQ571858	149
DQ597341	141

DQ570994	129
DQ572892	125
DQ571419	121
DQ598646	114
DQ596932	98
DQ594740	78
DQ570812	78
DQ592932	77
DQ597997	75
DQ571003	65
DQ593752	65
DQ584197	55
DQ571591	50
DQ571955	49
DQ575884	48
DQ593407	47
DQ584698	41
DQ596587	38
DQ578783	33
DQ570540	33
DQ570940	32
DQ571524	31
DQ570992	30
DQ571589	29
DQ597397	26
DQ575660	26
DQ596805	24
DQ597403	22
DQ570728	21
DQ600515	18
DQ587229	16
DQ572800	16
DQ597218	16
DQ593636	15
DQ596155	15
DQ598180	14
DQ579049	14
DQ594465	14
DQ598183	12
DQ576872	11
DQ576900	11
DQ590404	11
DQ598252	10
DQ598008	10
DQ598167	9

DQ571873	9
DQ577008	9
DQ581533	8
DQ595292	8
DQ601526	8
DQ570956	8
DQ575746	8
DQ570999	7
DQ598378	6
DQ580854	6
DQ571823	6
DQ596603	6
DQ600174	6
DQ573323	6
DQ571388	6
DQ576659	5
DQ600517	5
DQ591143	5
DQ592953	5
DQ594453	5
DQ586832	5
DQ573352	5
DQ577356	5
DQ585569	4
DQ593621	4
DQ576604	4
DQ576925	4
DQ596538	4
DQ598130	4
DQ596327	4
DQ590830	4
DQ571422	4
DQ575064	3
DQ596097	3
DQ596516	3
DQ584569	3
DQ576880	3
DQ571425	3
DQ573293	3
DQ590455	3
DQ578996	3
DQ572216	3
DQ596756	3
DQ579145	3
DQ597887	3

DQ597945	3
DQ596936	3
DQ576766	3
DQ571550	3
DQ596183	3
DQ596670	3
DQ576918	3
DQ575488	3

Supplementary Table S3E: Genomic distribution of the genes encoding piRNAs expressed in breast cancer or mammary epithelial MCF10A cells. Number of copies and nature of the transcription units where piRNAs mapping to unique genomic locations are encoded are indicated.

**Cell line**	**piRNAs identified**	**Multiple copies**			**Single copy**							
		**N.er of piRNAs**	**Read count**	**% of reads**	**N.er of piRNAs**	**Read count**	**% of reads**	**Inter genic**	**Intra genic**	***mRNA***	***snoRNA***	***snoRNA in mRNA***
MCF10A	138	47	21904	11.8%	91	163571	88.2%	25	66	*25*	*3*	*38*
MCF-7	78	17	9554	15.5%	61	52037	84.5%	12	49	*10*	*2*	*37*
SKBR3	75	15	1930	6.3%	60	28642	93.7%	13	47	*11*	*8*	*28*
ZR-75.1	117	39	25418	18.2%	78	114366	81.8%	17	61	*23*	*2*	*36*

Supplementary Table S3F: Genomic distribution of the genes encoding human germline piRNAs from piRNABank. Number of copies and nature of the transcription units where piRNAs mapping to unique genomic locations are encoded are indicated.

**Number of piRNAs in piRNABank**	**Multiple copies**		**Single copy**						
	**N.er of piRNAs**	**% of reads**	**N.er of piRNAs**	**% of reads**	**Intergenic**	**Intragenic**	***mRNA***	***snoRNA***	***snoRNA in mRNA***
23439	4179	17.83%	19260	82.17%	12941	6319	*6203*	*21*	*95*

Supplementary Table S3G: Summary of piRNAs differentially expressed (p-value ≤0.05) in BC *vs* mammary epithelial MCF10A cells. Information relative to number of copies and nature of the transcription units where piRNAs mapping to unique genomic locations are encoded are indicated.

	**MCF-7 *vs* MCF10A**				**ZR-75.1 *vs* MCF10A**				**SKBR3 *vs* MCF10A**						
**NCBI Accession**	**Normalized read count**		**Fold change**	**p-value Fisher's exact test**	**Normalized read count**		**Fold change**	**p-value Fisher's exact test**	**Normalized read count**		**Fold change**	**p-value Fisher's exact test**	***Copy in the genome***	***Genomic loci***	***Name***
	**MCF10A**	**MCF-7**			**MCF10A**	**ZR-75.1**			**MCF10A**	**SKBR3**					
DQ597347	9514	1867	-5.1	0.00E+00	12564	8084	-1.55	1.69E-229	8498	2218	-3.83	0.00E+00	*multiple*	*N/A*	*N/A*
DQ571526	2366	2366	1	1.00E+00	2272	667	-3.41	1.83E-205	1625	124	-13.15	0.00E+00	*multiple*	*N/A*	*N/A*
DQ590548	1867	812	-2.3	1.68E-95	1795	1979	1.1	3.00E-03	1385	3	-554	0.00E+00	*multiple*	*N/A*	*N/A*
DQ571335	1344	448	-3	2.00E-104	1250	423	-2.96	1.24E-95	852	532	-1.6	5.60E-18	*unique*	*intragenic*	*SNORD6*
DQ571500	976	338	-2.89	2.14E-72	770	436	-1.77	3.18E-22	607	282	-2.15	4.47E-28	*unique*	*intragenic*	*SNORD51 in EEF1*
DQ593744	581	273	-2.13	1.92E-26	436	259	-1.69	1.07E-11	394	78	-5.08	6.24E-52	*unique*	*intragenic*	*SNORD58A in RPL17*
DQ598650	500	101	-4.95	1.45E-64	407	129	-3.16	6.37E-35	359	106	-3.4	4.43E-33	*unique*	*intragenic*	*SNORD83B in RPL3*
DQ595536	404	57	-7.08	1.38E-65	355	66	-5.37	5.30E-49	311	34	-9.27	5.77E-57	*multiple*	*N/A*	*N/A*
DQ593356	338	55	-6.17	7.38E-51	320	194	-1.65	2.35E-08	282	64	-4.41	8.01E-34	*multiple*	*N/A*	*N/A*
DQ598651	189	2	-94.25	2.16E-53	212	2	-141.33	8.12E-61	190	10	-19	2.74E-44	*unique*	*intragenic*	*SNORD107*
DQ570812	56	11	-5.09	0.00E+00	61	34	-1.79	6.00E-03	52	3	-20.7	1.64E-13	*multiple*	*N/A*	*N/A*
DQ594740	56	18	-3.15	1.11E-05	61	24	-2.53	5.07E-05	52	22	-2.35	1.00E-03	*unique*	*intergenic*	*N/A*
DQ593752	44	25	-1.77	2.90E-02	52	26	-2.04	3.00E-03	43	17	-2.58	0.00E+00	*unique*	*intragenic*	*SNORA31 in TPT1*
DQ571955	34	2	-16.75	1.94E-08	40	18	-2.22	4.00E-03	32	3	-12.6	6.92E-08	*unique*	*intragenic*	*SRSF1*
DQ578783	22	2	-10.88	3.59E-05	26	4	-7.29	3.39E-05	22	3	-8.7	3.59E-05	*unique*	*intergenic*	*N/A*
DQ570940	20	2	-9.75	0.00E+00	25	4	-7.14	5.94E-05	21	10	-2.1	7.10E-02	*unique*	*intergenic*	*N/A*
DQ587229	10	2	-4.75	3.90E-02	12	2	-8	7.00E-03	10	3	-4	3.90E-02	*multiple*	*N/A*	*N/A*
DQ572800	10	2	-4.75	3.90E-02	12	8	-1.5	3.83E-01	10	3	-4	3.90E-02	*unique*	*intragenic*	*RBM15B*
DQ597218	10	2	-4.75	3.90E-02	12	10	-1.26	6.78E-01	10	3	-4	3.90E-02	*unique*	*intragenic*	*ABCB10*
DQ598263	177	311	1.75	1.32E-09	194	299	1.54	3.11E-06	178	359	2.02	4.08E-15	*unique*	*intragenic*	*PID1*
DQ598104	120	268	2.23	2.86E-14	129	212	1.65	6.03E-06	116	539	4.64	3.05E-66	*multiple*	*N/A*	*N/A*

DQ570994	90	566	6.32	1.03E-85	100	667	6.67	1.22E-103	86	86	1	1.00E+00	*unique*	*intragenic*	*ABCA2*
DQ572892	85	126	1.49	4.00E-03	95	221	2.32	1.40E-12	83	229	2.75	5.57E-17	*unique*	*intragenic*	*SNORD71 in AP1G1*
DQ597997	51	103	2.01	3.35E-05	59	100	1.71	1.00E-03	49	178	3.66	5.61E-19	*multiple*	*N/A*	*N/A*
DQ571003	44	157	3.54	4.22E-16	52	95	1.83	0.00E+00	43	94	2.21	1.12E-05	*multiple*	*N/A*	*N/A*
DQ584698	27	38	1.43	1.36E-01	33	60	1.85	5.00E-03	27	49	1.81	1.17E-02	*unique*	*intragenic*	*CPA6*
DQ571550	2	2	1.33	1.00E+00	2	12	7.67	1.30E-02	2	53	35	8.47E-14	*multiple*	*N/A*	*N/A*
DQ590455	2	20	13	6.59E-05	2	12	7.67	1.30E-02	2	3	1.67	1.00E+00	*multiple*	*N/A*	*N/A*
DQ596670	2	51	34	1.63E-13	2	4	2.33	6.87E-01	2	51	34	1.63E-13	*unique*	*intragenic*	*SNORD89*
DQ597945	2	2233	1488.33	0.00E+00	2	23516	15677.33	0.00E+00	2	21	14.17	3.58E-05	*multiple*	*N/A*	*N/A*

N/A, not applicable.

Supplementary Table S3H: Summary of piRNAs differentially expressed in cancerous *vs* mammary epithelial MCF10A cells. Number of copies and nature of the transcription units where piRNAs mapping to unique genomic locations are encoded are indicated.

**Differential expression Comparison**	**Regulated**	**Multiple**		**Unique**						
		**N.er of piRNAs**	**%**	**N.er of piRNAs**	**%**	**Inter genic**	**Intra genic**	***mRNA***	***snoRNA***	***snoRNA in mRNA***
BC *vs* MCF10A cells	30	13	43.3%	17	56.7%	3	14	*6*	*3*	*5*

Supplementary Table S4A: Genomic distribution of the genes encoding piRNAs expressed in in exponentially growing (G) or growth arrested (A) *wt*, Ct- and Nt- ERβ+ MCF-7 cells. Number of copies and nature of the transcription units where piRNAs mapping to unique genomic locations are encoded are indicated.

**Cell line**	**piRNAs identified**	**Multiple copies**			**Single copy**							
		**N.er of piRNAs**	**Read count**	**% of reads**	**N.er of piRNAs**	**Read count**	**% of reads**	**Inter genic**	**Intra genic**	***mRNA***	***snoRNA***	***snoRNA in mRNA***
MCF-7_G	65	15	3084	15.3%	50	17113	84.7%	9	41	*9*	*2*	*30*
MCF-7_A	65	12	1750	7.9%	53	20476	92.1%	12	41	*8*	*3*	*30*
Ct-ERβ_G	58	12	1875	13.7%	46	11877	86.3%	7	39	*7*	*2*	*30*
Ct-ERβ_A	72	14	2959	14.8%	58	17083	85.2%	12	46	*13*	*2*	*31*
Nt-ERβ_G	71	16	2482	12.2%	55	17875	87.8%	15	40	*10*	*1*	*29*
Nt-ERβ_A	71	13	2472	12.4%	58	17503	87.6%	14	44	*9*	*2*	*33*

Supplementary Table S4B: Number of raw (not normalized) reads that mapped to each identified piRNA in the libraries from exponentially growing (G) *wt* MCF-7 cells, measured in the three technical replicates. piRNAs are named in accordance to NCBI accession number.

**NCBI Accession**	**Raw read count**		
	**MCF-7_G_1**	**MCF-7_G_2**	**MCF-7_G_3**
DQ587153	3599	3838	3926
DQ595186	2594	2799	2898
DQ598677	2197	2161	2270
DQ597484	1183	1158	1234
DQ590013	1099	1167	1236
DQ596805	1082	1095	1060
DQ594556	754	806	808
DQ593039	713	761	816
DQ597945	681	717	753
DQ593538	673	735	794
DQ571526	658	709	745
DQ597347	498	517	541
DQ571524	417	440	469
DQ598675	388	354	388
DQ570687	317	381	360
DQ590548	271	269	296
DQ597482	263	314	312
DQ595807	237	279	264
DQ582264	218	198	237
DQ570994	186	190	213
DQ592931	159	144	167
DQ569993	156	213	236
DQ571335	131	135	152
DQ593767	113	107	134
DQ571500	94	82	93
DQ598263	66	83	70
DQ596993	64	73	78
DQ593744	56	74	80
DQ598104	48	66	76
DQ598428	37	38	45
DQ587269	33	37	27
DQ598646	29	18	16
DQ571003	29	20	27

DQ593574	28	39	37
DQ582566	28	20	27
DQ572892	25	26	27
DQ571858	21	17	34
DQ597997	17	19	21
DQ571419	16	11	23
DQ595536	15	11	10
DQ571591	14	10	14
DQ570698	14	14	11
DQ588040	13	16	20
DQ593356	11	11	13
DQ598650	9	15	23
DQ596932	9	9	8
DQ601526	9	7	5
DQ571511	8	10	16
DQ597397	8	1	1
DQ570540	7	4	4
DQ597975	5	8	11
DQ596992	5	4	6
DQ571589	4	1	1
DQ570728	4	1	1
DQ584197	4	6	4
DQ595023	4	1	1
DQ575884	4	4	1
DQ600174	4	1	1
DQ593752	4	1	4
DQ592932	4	6	1
DQ593407	1	4	1
DQ584698	1	4	1
DQ590455	1	4	1
DQ596670	1	14	4
DQ593636	1	1	6

Supplementary Table S4C: Number of raw (not normalized) reads that mapped to each identified piRNA in the libraries from growth-arrested (A) *wt* MCF-7 cells, measured in the three technical replicates. piRNAs are named in accordance to NCBI accession number.

**NCBI Accession**	**Raw read count**		
	**MCF-7_A_1**	**MCF-7_A_2**	**MCF-7_A_3**
DQ598677	6181	5989	5994
DQ587153	5285	5739	5824
DQ595186	2599	2758	2876
DQ597484	880	922	878
DQ597347	860	852	883
DQ593538	716	771	793
DQ595807	708	688	717
DQ594556	703	752	772
DQ593039	571	516	566
DQ571335	357	367	370
DQ597945	297	299	357
DQ590548	295	288	290
DQ569993	291	324	294
DQ570687	208	221	231
DQ570994	202	202	202
DQ571500	170	160	153
DQ571526	170	166	171
DQ582264	144	145	143
DQ596993	105	92	143
DQ592931	90	96	98
DQ598104	85	79	81
DQ598263	85	96	98
DQ593767	69	50	72
DQ597975	57	56	66
DQ587269	54	47	60
DQ593744	54	44	58
DQ598428	52	55	63
DQ572892	47	61	41
DQ593574	42	54	67
DQ596992	34	50	46
DQ582566	32	45	33
DQ571511	26	34	22
DQ598646	22	24	24
DQ588040	21	19	25

DQ590013	21	28	36
DQ571419	21	28	26
DQ596805	18	23	23
DQ571858	16	19	32
DQ575660	16	12	20
DQ571591	16	13	8
DQ598650	16	30	32
DQ596932	13	4	10
DQ593356	11	11	11
DQ570540	10	5	9
DQ571003	9	5	8
DQ570698	8	8	18
DQ571589	6	1	1
DQ593407	6	1	1
DQ570728	5	8	4
DQ598008	4	1	1
DQ597397	4	4	1
DQ595536	4	10	6
DQ597997	4	1	4
DQ584197	4	10	8
DQ597482	4	5	1
DQ594740	4	5	4
DQ596587	4	5	6
DQ595023	1	10	4
DQ584698	1	4	1
DQ598651	1	1	4
DQ578783	1	4	1
DQ570812	1	4	4
DQ596670	1	1	4
DQ597403	1	1	4
DQ593636	1	1	4

Supplementary Table S4D: Number of raw (not normalized) reads that mapped to each identified piRNA in the libraries from exponentially growing (G) Ct- ERβ+ cells, measured in the three technical replicates. piRNAs are named in accordance to NCBI accession number.

**NCBI Accession**	**Raw read count**		
	**Ct-ERβ_G_1**	**Ct-ERβ_G_2**	**Ct-ERβ_G_3**
DQ598677	2195	2070	2148
DQ587153	3214	3351	3531
DQ595186	2440	2547	2759
DQ597945	870	959	981
DQ597347	524	542	624
DQ597484	657	736	796
DQ593039	313	394	354
DQ593538	387	424	480
DQ595807	348	370	380
DQ594556	657	649	738
DQ571335	103	102	124
DQ582264	99	90	93
DQ569993	139	162	174
DQ571526	106	132	121
DQ598263	22	23	22
DQ590548	175	195	194
DQ570687	144	165	156
DQ571500	78	91	78
DQ592931	99	63	121
DQ597975	6	4	9
DQ570994	127	160	157
DQ593767	42	43	44
DQ596993	54	55	52
DQ590013	10	4	1
DQ596992	5	10	9
DQ598104	27	29	41
DQ582566	13	16	13
DQ587269	41	30	44
DQ593356	11	22	16
DQ593574	27	30	37
DQ598650	25	12	12
DQ598428	43	37	46
DQ572892	15	10	16
DQ598646	8	8	16

DQ571858	1	11	9
DQ593744	26	38	43
DQ571511	13	9	15
DQ588040	5	17	11
DQ571419	5	12	6
DQ595536	12	10	17
DQ584197	5	12	17
DQ597482	1	4	4
DQ571591	7	12	8
DQ571589	6	1	1
DQ571003	4	10	12
DQ570698	11	7	9
DQ575660	1	4	5
DQ571524	4	1	1
DQ593407	4	4	4
DQ596932	1	1	9
DQ570999	1	1	4
DQ597397	5	1	1
DQ593752	4	1	1
DQ592932	1	5	1
DQ596670	1	1	4
DQ596587	1	6	8
DQ598130	1	1	4
DQ596155	1	1	4

Supplementary Table S4E: Number of raw (not normalized) reads that mapped to each identified piRNA in the libraries from exponentially growing (G) Nt- ERβ+ cells, measured in the three technical replicates. piRNAs are named in accordance to NCBI accession number.

**NCBI Accession**	**Raw read count**		
	**Nt-ERβ_G_1**	**Nt-ERβ_G_2**	**Nt-ERβ_G_3**
DQ598677	4224	4165	4384
DQ587153	2348	2519	2671
DQ595186	2526	2660	2844
DQ597484	729	731	799
DQ597347	775	762	788
DQ593538	535	545	580
DQ594556	806	829	843

DQ593039	3910	4003	4349
DQ597945	908	996	997
DQ595807	390	372	404
DQ590548	437	462	475
DQ569993	182	187	236
DQ570687	134	144	131
DQ571335	99	126	122
DQ571526	148	140	162
DQ597975	25	35	35
DQ571500	70	78	84
DQ592931	99	108	114
DQ582264	96	86	84
DQ570994	150	139	178
DQ590013	5	8	4
DQ598104	44	30	38
DQ596992	50	55	63
DQ598428	52	44	35
DQ596993	35	31	37
DQ598263	227	214	180
DQ593574	28	32	33
DQ593767	77	79	75
DQ572892	16	30	14
DQ587269	37	44	44
DQ593744	31	26	28
DQ582566	65	74	57
DQ571858	7	11	16
DQ598650	18	19	25
DQ598646	16	23	14
DQ593356	11	10	9
DQ571419	17	13	12
DQ588040	12	22	26
DQ597997	37	45	37
DQ571511	6	10	10
DQ597397	5	1	1
DQ594740	1	7	4
DQ596932	1	5	7
DQ571003	11	14	14
DQ575660	4	1	6
DQ595536	23	19	22
DQ570698	7	10	12

DQ597403	4	1	1
DQ570728	1	5	1
DQ571591	7	6	1
DQ584197	12	9	11
DQ593407	1	4	7
DQ600515	1	1	4
DQ596587	4	1	1
DQ571589	1	4	6
DQ596538	1	8	1
DQ571823	5	4	1
DQ590404	35	35	47
DQ597341	30	24	30
DQ598167	1	10	7
DQ575884	7	1	9
DQ573352	4	1	8
DQ570940	8	9	12
DQ570540	4	4	9
DQ598175	7	8	5
DQ598252	1	4	1
DQ597482	4	1	4
DQ578783	90	111	100
DQ593752	1	10	4
DQ592932	1	7	1
DQ598675	1	4	1

Supplementary Table S4F: Summary of piRNAs differentially expressed (α <0.05 with Wilcoxon Mann-Whitney test) in exponentially growing (G) *vs* growth arrested (A) *wt* MCF-7 cells, or in ERβ+ *vs* ERβ- MCF-7 cells. Number of copies and nature of the transcription units where piRNAs mapping to unique genomic locations are encoded are indicated.

Pairwise comparisons	piRNAs identified	Multiple copies		Single copy						
		N.er of piRNA	% of reads	N.er of piRNA	% of reads	Intergenic	Intragenic	*mRNA*	*snoRNA*	*snoRNA in mRNA*
G/A *wt* MCF-7 cells	39	8	20.5%	31	79.5%	7	24	*7*	*1*	*16*
ERβ+/ERβ- cells	25	8	32%	17	68%	4	13	*4*	*0*	*9*

Supplementary Table S4G: Summary of piRNAs differentially expressed (α <0.05 with Wilcoxon Mann-Whitney test) in exponentially growing (G) *vs* growth arrested (A) MCF-7 cells. Information relative to number of copies and nature of the transcription units where piRNAs mapping to unique genomic locations are encoded are indicated.

	Normalized read count							Fold change				
	Exponentially growing			Growth arrested								
NCBI Accession	MCF-7_G_1	MCF-7_G_2	MCF-7_G_3	MCF-7_A_1	MCF-7_A_2	MCF-7_A_3	α Wilcoxon Mann-Whitney	MCF-7 G/A	p-value Fisher's exact test	*Copies in the genome*	*Genomic loci*	*Name*
DQ601526	8	6	4	0	0	0	0.0369	6	3.81E-06	unique	intragenic	RBM33
DQ598675	387	337	354	0	0	0	0.0369	359.33	0.00E+00	unique	intragenic	KIF5C
DQ596805	1081	1043	968	15	19	19	0.0463	58.34	0.00E+00	unique	intergenic	N/A
DQ571524	416	419	428	0	0	0	0.0369	421	0.00E+00	multiple	N/A	N/A
DQ571003	28	18	24	7	3	6	0.0495	4.38	1.93E-09	multiple	N/A	N/A
DQ590013	1098	1112	1129	18	23	30	0.0495	47.03	0.00E+00	unique	intragenic	KIAA0825
DQ597482	262	299	284	3	3	0	0.0463	140.83	1.63E-243	multiple	N/A	N/A
DQ597997	16	17	18	3	0	3	0.0463	8.5	3.13E-10	multiple	N/A	N/A
DQ571526	657	675	680	150	143	144	0.0495	4.6	2.36E-245	multiple	N/A	N/A
DQ597945	680	683	687	263	258	302	0.0495	2.49	1.42E-122	multiple	N/A	N/A

DQ592931	158	136	152	79	82	82	0.0463	1.84	5.83E-15	unique	intragenic	RNY4
DQ593767	112	101	122	60	42	60	0.0463	2.07	4.19E-15	unique	intragenic	SNORD58B in RPL17
DQ582264	217	188	216	127	125	120	0.0495	1.67	1.54E-15	unique	intergenic	N/A
DQ597484	1182	1103	1127	782	798	743	0.0495	1.47	1.66E-49	unique	intergenic	N/A
DQ570687	316	362	328	184	191	195	0.0495	1.76	9.41E-29	unique	intragenic	SNORD43 in RPL3
DQ593039	712	725	745	507	446	479	0.0495	1.52	4.69E-37	unique	intergenic	N/A
DQ594556	753	768	737	624	650	654	0.0495	1.17	2.02E-07	unique	intragenic	SNORD15A in RPS3
DQ593356	10	10	11	9	9	8	0.0431	1.19	5.12E-01	multiple	N/A	N/A
DQ595186	2593	2668	2647	2310	2388	2437	0.0495	1.11	1.40E-11	unique	intragenic	SNORD58C in RPL17
DQ570994	185	180	194	179	174	170	0.0495	1.07	2.72E-01	unique	intragenic	ABCA2
DQ593744	55	70	72	47	37	48	0.0495	1.49	3.26E-04	unique	intragenic	SNORD58A in RPL17
DQ593538	672	700	725	636	667	671	0.0495	1.06	4.97E-02	unique	intragenic	SNORD17 in SNX5
DQ587153	3598	3659	3587	4699	4969	4936	0.0495	-1.35	2.78E-157	unique	intragenic	SNORD57 in NOP56
DQ587269	32	34	24	47	40	50	0.0495	-1.52	2.17E-03	unique	intragenic	SNORD20 in NCL
DQ598428	36	35	40	45	47	53	0.0495	-1.31	3.87E-02	unique	intragenic	SNORD1B in SNHG16
DQ571500	93	77	84	150	138	129	0.0495	-1.64	2.93E-10	unique	intragenic	SNORD51 in EEF1B2
DQ597347	497	492	493	764	737	748	0.0495	-1.52	1.83E-37	multiple	N/A	N/A
DQ596993	63	69	70	92	79	120	0.0495	-1.44	6.84E-05	unique	intragenic	SNORD110 in NOP56
DQ572892	24	24	24	41	52	34	0.0369	-1.76	1.16E-04	unique	intragenic	SNORD71 in AP1G1
DQ569993	155	202	215	258	280	248	0.0495	-1.37	5.68E-09	unique	intragenic	SNORD98 in CCAR1
DQ598677	2196	2060	2074	5495	5186	5080	0.0495	-2.49	0.00E+00	unique	intergenic	N/A
DQ571335	130	128	138	317	317	313	0.0463	-2.39	5.86E-53	unique	intragenic	SNORD6
DQ595807	236	265	240	629	595	607	0.0495	-2.47	8.59E-108	unique	intragenic	SNORD1B in SNHG16
DQ571511	7	9	14	22	29	18	0.0495	-2.3	1.10E-04	unique	intragenic	SNORD49A in C17
DQ596992	4	3	5	29	42	38	0.0495	-9.08	9.48E-21	unique	intragenic	EFCAB11
DQ597975	4	7	9	50	48	55	0.0495	-7.65	1.34E-26	unique	intergenic	N/A
DQ575660	0	0	0	13	10	16	0.0369	-13	3.61E-12	unique	intragenic	PLCH1
DQ594740	0	0	0	3	3	3	0.0253	-3	3.91E-03	unique	intergenic	N/A
DQ596587	0	0	0	3	3	4	0.0339	-3.33	1.95E-03	unique	intragenic	SNORA54 in RPS3

N/A, not applicable.

Supplementary Table S4H: Summary of piRNAs differentially expressed (α <0.05 with Wilcoxon Mann-Whitney test) in Ct- and Nt- ERβ+ *vs wt* (ERβ-) MCF-7 cells. Information relative to number of copies and nature of the transcription units where piRNAs mapping to unique genomic locations are encoded are indicated.

	Normalized read count														
	wt MCF-7			Ct-ERβ			Nt-ERβ				Fold change				
NCBI Accession	G_1	G_2	G_3	G_1	G_2	G_3	G_1	G_2	G_3	α Wilcoxon Mann-Whitney	ERβ-/ERβ+	p-value Fisher's exact test	*Copies in the genome*	*Genomic loci*	*Name*
DQ601526	5	4	2	0	0	0	0	0	0	0.006	3.67	5.63E-06	multiple	N/A	N/A
DQ598675	263	229	240	0	0	0	0	2	0	0.011	732	0.00E+00	unique	intragenic	KIF5C
DQ596805	734	708	657	0	0	0	0	0	0	0.006	700	0.00E+00	unique	intergenic	N/A
DQ571524	282	284	290	3	0	0	0	0	0	0.011	570.67	0.00E+00	multiple	N/A	N/A
DQ590013	745	755	766	9	3	0	3	5	2	0.02	206	0.00E+00	unique	intragenic	KIAA0825
DQ597482	178	203	193	0	3	3	2	0	2	0.019	114.8	1.59E-256	multiple	N/A	N/A
DQ571526	446	458	462	105	126	108	98	90	99	0.02	4.36	5.65E-230	unique	intragenic	RBM33
DQ571003	19	12	16	3	9	10	7	8	8	0.02	2.09	5.26E-04	multiple	N/A	N/A
DQ571858	14	10	20	0	10	7	4	6	9	0.028	2.44	7.25E-05	unique	intragenic	SNORD4A in RPL23A
DQ582264	147	128	146	98	85	83	63	55	51	0.02	1.94	8.92E-22	unique	intergenic	N/A
DQ570687	215	246	223	143	157	140	89	93	80	0.02	1.95	4.30E-35	unique	intragenic	SNORD43 in RPL3
DQ593767	76	69	83	41	40	39	51	51	45	0.02	1.71	4.52E-09	unique	Intragenic	SNORD58B in RPL17
DQ597484	802	749	765	656	705	716	486	474	490	0.02	1.31	4.20E-25	unique	intergenic	N/A
DQ593744	37	47	49	25	35	38	20	16	17	0.039	1.76	2.68E-06	unique	intragenic	SNORD58A in RPL17
DQ593538	456	475	492	386	406	432	357	353	356	0.02	1.24	8.20E-11	unique	intragenic	SNORD17 in SNX5
DQ598104	32	42	47	26	27	36	29	19	23	0.039	1.51	7.54E-04	multiple	N/A	N/A
DQ595536	10	6	6	11	9	14	15	12	13	0.038	-1.68	3.03E-02	multiple	N/A	N/A
DQ594556	511	521	501	656	621	664	538	537	517	0.039	-1.15	1.99E-06	unique	intragenic	SNORD15A in RPS3
DQ587269	22	23	16	40	28	39	24	28	26	0.02	-1.52	4.37E-03	unique	Intragenic	SNORD20 in NCL
DQ597347	337	334	335	523	519	561	517	494	483	0.02	-1.54	9.62E-36	multiple	N/A	N/A
DQ598677	1491	1399	1408	2194	1984	1934	2821	2702	2692	0.02	-1.67	1.43E-241	unique	intergenic	N/A

DQ597945	462	464	467	869	919	883	606	646	612	0.02	-1.63	2.53E-64	multiple	N/A	N/A
DQ595807	160	180	163	347	354	341	260	241	248	0.02	-1.78	1.06E-33	unique	intragenic	SNORD1B in SNHG16
DQ584197	2	3	2	4	11	14	7	5	6	0.02	-3.36	1.23E-03	unique	intragenic	SNORD36C in RPL7A
DQ596992	3	2	3	4	9	7	33	35	38	0.02	-7.88	4.37E-14	unique	intragenic	EFCAB11

N/A, not applicable.

Supplementary Table S4I: Summary of piRNAs differentially expressed (α <0.05 with Wilcoxon Mann-Whitney test) in in exponentially growing (G) *vs* growth arrested (A) ERβ+ MCF-7 cells. Information relative to number of copies and nature of the transcription units where piRNAs mapping to unique genomic locations are encoded are indicated.

	**Normalized read count**																	
	**Exponentially growing**						**Growth arrested**											
	**Nt-ERβ**			**Ct-ERβ**			**Nt-ERβ**			**Ct-ERβ**				**Fold change**				
**NCBI Accession**	**G_1**	**G_2**	**G_3**	**G_1**	**G_2**	**G_3**	**A_1**	**A_2**	**A_3**	**A_1**	**A_2**	**A_3**	**α Wilcoxon Mann-Whitney**	**ERβ+ G/A**	**p-value Fisher's exact test**	***Copy in the genome***	***Genomic loci***	***Name***
DQ595536	15	12	13	11	9	14	4	5	9	7	8	8	0.005	1.8	2.68E-03	multiple	N/A	N/A
DQ594556	538	537	517	656	621	664	454	461	474	254	252	252	0.004	1.65	6.30E-79	unique	intragenic	SNORD15A in RPS3
DQ595186	1686	1726	1746	2439	2442	2485	876	929	877	988	1039	1020	0.004	2.19	0.00E+00	unique	intragenic	SNORD58C in RPL17
DQ570994	100	90	109	126	152	141	63	62	70	64	59	73	0.004	1.84	4.34E-23	unique	intragenic	ABCA2
DQ598104	29	19	23	26	27	36	44	38	46	34	34	34	0.016	-1.44	3.80E-04	multiple	N/A	N/A
DQ598677	2821	2702	2692	2194	1984	1934	4467	4291	4205	4310	4031	3975	0.004	-1.76	0.00E+00	unique	intergenic	N/A
DQ597347	517	494	483	523	519	561	753	739	758	697	729	722	0.004	-1.42	9.35E-54	multiple	N/A	N/A
DQ571526	98	90	99	105	126	108	122	129	136	145	159	147	0.006	-1.34	2.48E-08	multiple	N/A	N/A
DQ569993	121	121	144	138	154	156	177	176	208	161	166	140	0.016	-1.23	6.02E-06	unique	intragenic	SNORD98 in CCAR1
DQ598646	10	14	8	7	7	14	16	27	27	18	12	14	0.015	-1.9	3.86E-05	unique	intragenic	SNORD85 in PUM1
DQ593574	18	20	20	26	28	32	36	31	38	24	28	35	0.045	-1.33	8.76E-03	unique	intragenic	SNORD91A in TSR1
DQ582264	63	55	51	98	85	83	74	92	88	166	156	189	0.037	-1.76	6.58E-22	unique	intergenic	N/A
DQ571500	46	50	51	77	86	69	80	81	85	79	95	96	0.025	-1.36	4.25E-06	unique	intragenic	SNORD51 in EEF1B2

DQ571335	65	81	74	102	97	111	136	140	129	180	183	201	0.004	-1.83	1.82E-30	unique	intragenic	SNORD6
DQ572892	10	19	8	14	9	14	31	33	36	21	21	22	0.004	-2.22	3.71E-09	unique	intragenic	SNORD71 in AP1G1
DQ571858	4	6	9	0	10	7	19	10	14	15	7	13	0.016	-2.17	7.47E-05	unique	intragenic	SNORD4A in RPL23A
DQ597975	16	22	21	5	3	7	88	134	104	71	72	78	0.004	-7.39	1.15E-90	unique	intergenic	N/A
DQ590013	3	5	2	9	3	0	45	40	50	46	50	61	0.004	-13.27	9.80E-62	unique	intragenic	KIAA0825
DQ594740	0	4	2	0	0	0	5	5	6	30	44	35	0.003	-20.83	2.40E-30	unique	intergenic	N/A
DQ596805	0	0	0	0	0	0	134	136	144	54	50	62	0.002	-96.67	8.42E-176	unique	intergenic	N/A
DQ593636	0	0	0	0	0	0	0	2	2	2	2	0	0.019	-1.33	3.90E-03	unique	intragenic	SNORD100 in RPS12

N/A, not applicable.

Supplementary Table S4J: Number of raw (not normalized) reads that mapped to each identified piRNA in the libraries from growth-arrested (A) Ct- ERβ+ cells, measured in the three technical replicates. piRNAs are named in accordance to NCBI accession number.

NCBI Accession	Raw read count		
	**Ct-ERβ_A_1**	**Ct-ERβ_A_2**	**Ct-ERβ_A_3**
DQ598677	6390	6173	6285
DQ587153	3467	3772	3880
DQ595186	1465	1591	1614
DQ597945	1235	1331	1350
DQ597347	1034	1117	1142
DQ597484	929	932	979
DQ593039	682	702	717
DQ593538	484	509	499
DQ595807	464	496	510
DQ594556	377	387	399
DQ571335	268	281	319
DQ582264	247	240	300
DQ569993	239	255	223
DQ571526	216	244	234
DQ598263	166	159	192
DQ590548	156	153	188
DQ570687	143	129	129
DQ571500	118	146	153
DQ592931	109	137	133
DQ597975	106	111	124
DQ570994	96	91	116
DQ593767	83	86	75
DQ596805	81	77	99
DQ596993	78	80	112
DQ590013	69	77	98
DQ596992	57	51	49
DQ598104	52	53	54
DQ578783	48	60	70
DQ582566	47	54	56
DQ594740	45	69	56
DQ587269	43	53	41
DQ593356	39	44	47
DQ593574	37	44	56
DQ598650	37	26	40

DQ598428	36	35	43
DQ572892	32	33	36
DQ598646	28	20	23
DQ597997	26	31	41
DQ571858	23	12	21
DQ593744	23	43	32
DQ571511	18	19	21
DQ588040	17	29	23
DQ571419	13	9	20
DQ595536	12	13	13
DQ590404	10	6	5
DQ584197	10	12	12
DQ597482	10	5	11
DQ571591	10	15	13
DQ571589	9	1	4
DQ575884	9	1	4
DQ571003	9	8	7
DQ570698	9	4	14
DQ571873	7	1	1
DQ575660	6	4	7
DQ571823	5	5	1
DQ571524	5	7	1
DQ570540	5	1	1
DQ593407	5	7	6
DQ570728	4	7	7
DQ596932	4	4	6
DQ598294	4	1	1
DQ593636	4	4	1
DQ584698	1	7	8
DQ597397	1	10	8
DQ598651	1	1	6
DQ571388	1	1	4
DQ570812	1	1	5
DQ593752	1	7	4
DQ593415	1	4	1
DQ598675	1	1	8
DQ595179	1	1	4
DQ597403	1	8	1

Supplementary Table S4K: Number of raw (not normalized) reads that mapped to each identified piRNA in the libraries from growth-arrested (A) Nt- ERβ+ cells, measured in the three technical replicates. piRNAs are named in accordance to NCBI accession number.

NCBI Accession	Raw read count		
	Nt-ERβ_A_1	Nt-ERβ_A_2	Nt-ERβ_A_3
DQ598677	6627	6450	6696
DQ587153	3037	3210	3405
DQ595186	1301	1397	1398
DQ597484	1216	1207	1284
DQ597347	1118	1111	1208
DQ593538	812	835	902
DQ594556	675	694	755
DQ593039	638	637	678
DQ597945	591	562	619
DQ595807	513	526	562
DQ590548	409	396	456
DQ569993	264	266	332
DQ570687	210	198	188
DQ571335	203	211	206
DQ596805	200	206	230
DQ571526	182	195	217
DQ597975	131	202	167
DQ571500	119	123	136
DQ592931	112	137	172
DQ582264	111	140	141
DQ570994	95	94	112
DQ590013	68	61	81
DQ598104	67	58	74
DQ596992	62	75	65
DQ598428	62	60	66
DQ596993	57	52	64
DQ598263	56	71	75
DQ593574	55	48	61
DQ593767	54	47	47
DQ572892	47	50	58
DQ587269	43	57	39
DQ593744	40	40	38
DQ582566	33	35	54
DQ571858	29	16	24

DQ598650	29	21	18
DQ598646	25	41	44
DQ593356	17	15	29
DQ571419	15	17	15
DQ588040	14	24	23
DQ597997	12	10	14
DQ571511	12	20	19
DQ597397	9	1	1
DQ594740	9	9	11
DQ596932	8	7	9
DQ571003	8	15	7
DQ575660	7	7	1
DQ595536	7	9	15
DQ570698	7	1	9
DQ593415	7	1	12
DQ597403	6	1	1
DQ595023	5	5	1
DQ570728	5	1	1
DQ571524	5	1	4
DQ571591	5	1	4
DQ596670	5	1	1
DQ584197	4	12	16
DQ593407	4	5	7
DQ600515	4	1	4
DQ570992	4	1	1
DQ596587	4	7	4
DQ584698	1	4	1
DQ571589	1	7	1
DQ596538	1	1	4
DQ575884	1	5	4
DQ601526	1	5	1
DQ600174	1	4	1
DQ570540	1	4	1
DQ578783	1	4	1
DQ593752	1	1	4
DQ593636	1	4	4
DQ596155	1	1	6

Supplementary Table S5A: Normalized piRNA read count in small RNA sequencing libraries from paired normal and tumor breast tissue samples. N.er 40, 45, 53 and 79 corresponds to patients TAX577740, TAX577745, TAX577453, and TAX577579, respectively.

	Normalized read count							
	Normal				Tumour			
**NCBI Accession**	**40N**	**45N**	**53N**	**79N**	**40T**	**45T**	**53T**	**79T**
DQ597347	30579	25755	11098	13936	12957	40164	17737	6661
DQ598677	7304	7148	5620	10268	11649	8685	13873	24226
DQ596670	3796	2782	8143	6838	1353	1307	1097	2596
DQ597484	2566	1566	1633	885	980	1342	3092	1962
DQ598263	2367	441	300	240	788	285	185	1112
DQ598175	1924	3504	1433	1066	2616	851	953	328
DQ571003	1689	323	263	244	240	461	262	834
DQ592931	1685	618	1043	2232	733	264	2641	4130
DQ597975	1329	2839	5731	5530	1593	617	8428	2962
DQ571335	1007	4460	1707	2402	8373	2065	564	399
DQ575660	829	513	400	1069	255	305	460	508
DQ570940	597	185	137	161	487	101	131	840
DQ597403	577	88	21	69	14	59	0	121
DQ593039	523	59	2586	318	878	101	397	2233
DQ598183	475	782	432	1396	271	180	163	136
DQ582264	468	444	237	161	261	635	325	727
DQ598675	342	378	785	255	3478	104	488	362
DQ571419	326	1342	427	565	1601	374	284	334
DQ587153	322	553	632	208	444	598	244	213
DQ571526	321	171	400	114	96	245	144	427
DQ595807	321	1901	4514	3005	1624	1108	2451	2577
DQ570992	291	99	0	49	26	116	14	14
DQ580854	262	4	0	0	12	5	0	24
DQ598190	243	26	100	76	0	59	0	107
DQ598104	232	1397	4045	3822	2695	589	4596	2387
DQ594740	231	70	84	197	189	28	14	119
DQ578783	226	68	0	52	194	44	45	158
DQ571550	187	53	16	25	27	13	0	12
DQ590013	167	37	174	269	242	143	27	545
DQ596992	165	0	0	7	24	5	90	57
DQ569993	142	497	1875	2532	2339	501	1151	1464
DQ597341	139	92	132	67	82	35	45	59
DQ571823	114	14	0	7	43	5	0	85

DQ584698	109	27	63	67	224	13	135	71
DQ597397	102	32	0	13	10	19	0	12
DQ597997	98	55	32	0	77	9	27	32
DQ596309	98	481	406	724	428	30	86	292
DQ598008	95	152	785	403	130	35	163	502
DQ598252	93	59	358	76	6	51	0	16
DQ593538	88	704	353	181	1129	146	230	148
DQ593767	86	148	442	195	134	178	776	142
DQ582566	84	135	1965	291	236	22	72	32
DQ571524	77	0	0	0	0	8	0	14
DQ595023	74	210	47	43	118	105	68	59
DQ573323	73	250	300	231	194	116	14	14
DQ598167	72	4	0	0	20	5	0	6
DQ590404	72	9	0	0	0	5	0	8
DQ571031	52	55	0	16	6	26	0	0
DQ570728	44	323	1449	1479	829	175	673	1010
DQ570968	43	0	0	0	10	0	0	12
DQ593574	42	526	32	155	216	51	108	34
DQ573352	40	0	0	0	0	6	0	6
DQ593636	39	148	100	83	183	67	45	247
DQ598445	38	0	0	0	0	3	0	18
DQ598177	38	0	0	7	0	0	0	0
DQ598646	38	358	590	356	536	89	126	164
DQ596538	36	4	0	36	27	6	0	107
DQ596183	36	0	0	0	0	0	0	0
DQ571591	35	194	184	186	318	53	113	221
DQ592953	35	11	16	22	31	21	0	51
DQ598378	33	180	58	166	464	44	280	107
DQ592932	33	14	42	60	86	14	208	500
DQ571589	32	52	84	7	69	97	14	12
DQ572892	31	267	232	195	371	51	59	117
DQ587229	30	75	121	92	92	17	0	73
DQ576872	30	95	105	76	84	23	176	69
DQ584197	30	80	100	9	12	70	0	34
DQ588040	26	33	0	13	12	36	0	0
DQ593415	24	68	0	7	27	51	0	0
DQ598650	24	86	32	0	61	140	23	0
DQ595186	22	30	300	20	86	129	117	34
DQ597218	20	7	0	0	0	6	14	0
DQ596311	19	109	163	112	59	2	0	18

DQ588594	17	0	0	0	0	2	0	0
DQ570812	16	0	0	7	0	0	0	16
DQ597887	15	0	0	0	0	28	0	30
DQ596603	14	33	32	7	35	46	0	10
DQ576604	14	0	0	0	0	59	14	0
DQ596390	12	50	79	83	29	9	14	30
DQ593752	11	46	163	13	33	70	41	36
DQ593356	11	36	153	54	77	81	149	36
DQ598619	10	4	0	0	0	0	0	0
DQ571858	10	62	16	16	81	31	0	22
DQ594465	9	0	16	0	0	4	0	0
DQ596354	9	138	179	20	53	11	27	0
DQ587514	8	4	274	0	6	21	14	0
DQ571873	8	0	0	0	8	0	0	0
DQ576200	8	17	0	0	0	2	0	0
DQ571511	7	53	26	13	35	28	0	20
DQ598428	7	39	0	9	14	36	14	12
DQ597916	7	0	0	0	0	0	0	0
DQ590835	7	0	0	0	0	0	0	0
DQ571500	6	11	74	7	27	29	14	18
DQ593744	6	52	147	0	31	69	77	12
DQ600515	6	0	0	0	0	3	0	0
DQ597960	5	19	0	20	26	58	27	34
DQ596805	4	0	0	0	6	0	0	63
DQ575884	4	0	0	0	0	8	0	0
DQ571388	4	16	0	0	0	3	0	0
DQ596830	3	0	0	0	0	0	0	0
DQ587269	3	37	53	25	33	22	41	55
DQ581533	3	0	0	0	0	0	0	0
DQ596993	3	26	16	13	45	19	0	24
DQ590830	3	7	16	20	0	9	0	16
DQ570956	3	4	242	7	0	10	27	12
DQ577008	3	0	0	0	0	0	0	0
DQ593407	3	0	0	0	0	9	32	8
DQ575656	3	11	74	20	6	5	0	36
DQ576917	0	0	0	0	8	0	0	0
DQ600514	0	0	16	0	0	0	0	0
DQ573999	0	6	0	0	20	3	32	0
DQ597945	0	0	0	0	0	0	14	0
DQ573287	0	0	0	0	6	0	0	0

DQ588101	0	0	0	7	0	6	0	0
DQ570994	0	0	0	0	0	9	32	26
DQ598651	0	14	0	11	8	6	0	0
DQ596863	0	10	42	0	0	0	0	0
DQ576918	0	0	105	49	65	0	0	233
DQ594556	0	0	0	0	41	5	0	0
DQ573706	0	0	0	0	0	0	63	0
DQ570540	0	9	0	0	0	2	0	0
DQ584649	0	0	0	0	12	0	0	0
DQ590548	0	0	0	0	0	0	0	6
DQ598300	0	0	0	7	0	0	0	0
DQ593939	0	0	0	0	6	0	0	0
DQ570698	0	4	0	0	0	2	0	0
DQ577772	0	145	16	269	407	40	18	0
DQ570687	0	10	0	0	0	10	0	6
DQ588486	0	0	0	0	10	0	0	0
DQ598130	0	0	0	0	0	2	0	0
DQ570999	0	0	0	0	0	2	0	0
DQ578832	0	0	0	0	6	0	0	6
DQ583325	0	0	0	0	20	0	0	6
DQ580405	0	0	0	0	0	2	0	0
DQ582827	0	0	0	9	0	0	0	0
DQ586711	0	0	0	0	0	0	14	0
DQ600994	0	0	0	9	0	0	0	0
DQ579145	0	0	0	0	0	0	14	0
DQ598016	0	7	0	7	22	10	0	12
DQ573293	0	0	0	0	18	0	0	389
DQ596306	0	4	0	0	0	0	0	0
DQ583332	0	0	0	0	12	0	0	6
DQ596587	0	9	0	11	6	0	0	0
DQ583674	0	13	0	0	0	8	0	0
DQ594453	0	0	16	0	0	0	0	0
DQ596155	0	11	16	0	39	3	0	8

Supplementary Table S5B: Summary of piRNAs differentially expressed (α <0.05 with Wilcoxon Mann-Whitney test) in cancer *vs* normal mammary tissues from 4 breast cancer patients (TAX577740, TAX577745, TAX577453 and TAX577579). Information relative to number of copies and nature of the transcription units where piRNAs mapping to unique genomic locations are encoded are indicated.

	**Normalized read count**													
	TAX5777**40**		TAX5777**45**		TAX5774**53**		TAX5775**79**			**Fold change**				
**NCBI Accession**	**N**	**T**	**N**	**T**	**N**	**T**	**N**	**T**	**α Wilcoxon Mann-Whitney**	**T/N**	**p-value Fisher's exact test**	***Copies in the genome***	***Genomic loci***	***Name***
DQ596670	3796	1353	2782	1307	8143	1097	6838	2596	0.021	-3.39	0.00E+00	*unique*	*intragenic: snoRNA*	*SNORD89*
DQ598183	475	271	782	180	432	163	1396	136	0.021	-4.11	0.00E+00	*multiple*	*N/A*	*N/A*
DQ597341	139	82	92	35	132	45	67	59	0.043	-1.95	1.90E-16	*unique*	*intergenic*	*N/A*
DQ598252	93	6	59	51	358	0	76	16	0.021	-8.03	1.58E-100	*unique*	*intergenic*	*N/A*
DQ596311	19	59	109	2	163	0	112	18	0.043	-5.10	2.10E-53	*unique*	*intragenic: snoRNA*	*SNORD113-8*
DQ598677	7304	11649	7148	8685	5620	13873	10268	24226	0.043	1.93	0.00E+00	*unique*	*intergenic*	*N/A*
DQ597960	5	26	19	58	0	27	20	34	0.021	3.30	5.55E-14	*unique*	*intergenic*	*N/A*
DQ570994	0	0	0	9	0	32	0	26	0.047	16.75	6.74E-21	*unique*	*intragenic: mRNA*	*ABCA2*

N/A, not applicable.

Supplementary Table S6A: Identification of the putative targets of eight piRNAs differentially expressed in paired normal and tumor breast tissue samples from 4 breast cancer patients.
Stringent alignment score (≥170) and energy threshold ≤-20.0 kcal/mol were used to identify potential piRNA binding sites by sequence complementarity to all known human RNAs (RefSeq) or to the RNAs detected in breast cancer tissue samples.

	**miRanda (RefSeq)**			**miRanda (expressed in breast ca)**		
**NCBI Accession**	**5'UTR**	**CDS**	**3'UTR**	**5'UTR**	**CDS**	**3'UTR**
DQ596670	42	78	221	3	33	78
DQ598183	35	201	203	9	68	66
DQ597341	95	541	114	30	231	38
DQ598252	11	45	6	3	18	2
DQ596311	92	458	476	14	189	180
DQ598677	60	346	146	9	161	51
DQ597960	24	195	168	3	92	58
DQ570994	41	80	148	11	45	51

Supplementary Table S6B: Putative DQ596670 piRNA target RNAs expressed in breast tumours. piRNAs targeting long non coding RNAs are shown in *italic*.

**Gene symbol**	**RefSeq n.er**	**Expression (normalized read count)**	**Alignment score**	**Energy (kcal/mol)**	**DQ596670 binding in RNA**	**Transcript category**
LOC653786	NR_003676	3	178	-26.84	-	ncRNA (pseudogene)
LOC646471	*NR_024498*	*24*	*173*	*-28.39*	-	*lncRNA*
ACACA	NM_198839	3161	173	-24.26	CDS	mRNA
ACTA2	NM_001613	16442	173	-22.37	3'UTR	mRNA
ADAM2	NM_001464	11	178	-26.13	CDS	mRNA
ADORA3	NM_000677	428	179	-26.43	CDS	mRNA
AMOTL1	NM_130847	797	172	-27.25	3'UTR	mRNA
ARFIP1	NM_014447	1200	172	-21.29	3'UTR	mRNA
ARHGAP9	NM_032496	85	177	-26.32	3'UTR	mRNA
ARL1	NM_001177	2805	183	-22.53	3'UTR	mRNA
ATXN1L	NM_001137675	772	171	-23.15	3'UTR	mRNA
BCAM	NM_001013257	4038	170	-22.81	3'UTR	mRNA
BMP1	NM_006129	1051	174	-26.30	3'UTR	mRNA
BMPER	NM_133468	1	178	-25.48	3'UTR	mRNA
BTBD9	NM_152733	510	177	-23.62	3'UTR	mRNA

C19orf12	NM_031448	377	174	-28.48	3'UTR	mRNA
C4orf33	NM_173487	192	181	-24.72	3'UTR	mRNA
CAND1	NM_018448	4221	171	-20.40	CDS	mRNA
CENPI	NM_006733	94	170	-20.53	CDS	mRNA
CHST8	NM_022467	4	176	-25.63	3'UTR	mRNA
CSNK1G1	NM_022048	546	172	-29.86	3'UTR	mRNA
DCUN1D5	NM_032299	590	171	-21.02	3'UTR	mRNA
DIO2	NM_000793	1018	170	-25.64	3'UTR	mRNA
DLC1	NM_006094	640	172	-24.09	3'UTR	mRNA
DOPEY2	NM_005128	1377	172	-24.74	CDS	mRNA
EIF4E2	NM_001282958	1383	170	-21.99	3'UTR	mRNA
EMD	NM_000117	977	180	-23.15	3'UTR	mRNA
ENOX2	NM_006375	295	172	-25.07	3'UTR	mRNA
EXD3	NM_001286823	92	178	-25.03	3'UTR	mRNA
EXOSC2	NM_014285	595	173	-23.42	3'UTR	mRNA
F2RL1	NM_005242	137	173	-22.86	CDS	mRNA
F5	NM_000130	37	173	-20.94	3'UTR	mRNA
FAM157A	NM_001145248	3	176	-22.60	CDS	mRNA
FAM157B	NM_001145249	2	176	-22.60	CDS	mRNA
FAM192A	NM_024946	1803	172	-20.14	5'UTR	mRNA
FAM19A1	NM_213609	1	170	-21.94	3'UTR	mRNA
FBXL7	NM_001278317	827	177	-28.15	3'UTR	mRNA
GABRE	NM_004961	10	171	-23.13	3'UTR	mRNA
GCH1	NM_000161	357	170	-23.40	3'UTR	mRNA
GPT2	NM_133443	1398	170	-21.28	CDS	mRNA
GRAMD1C	NM_017577	142	171	-20.18	CDS	mRNA
GRIK3	NM_000831	15	171	-28.03	3'UTR	mRNA
HERC3	NM_014606	218	172	-24.60	3'UTR	mRNA
HIF1A	NM_001530	2633	183	-23.08	3'UTR	mRNA
HIGD1A	NM_014056	1200	179	-20.94	3'UTR	mRNA
HSBP1	NM_001537	1946	181	-21.98	3'UTR	mRNA
IFIT2	NM_001547	722	170	-25.20	CDS	mRNA
IL21R	NM_021798	178	173	-24.31	CDS	mRNA
KBTBD11	NM_014867	33	174	-21.04	3'UTR	mRNA
KDM2B	NM_032590	852	173	-24.02	3'UTR	mRNA
LGALS8	NM_006499	1384	174	-29.02	3'UTR	mRNA
MAPKAP1	NM_024117	1665	187	-29.58	3'UTR	mRNA
MIA3	NM_198551	4216	184	-27.49	CDS	mRNA
NEK5	NM_199289	50	171	-23.33	CDS	mRNA
NFIA	NM_005595	3127	172	-23.58	3'UTR	mRNA

NSD1	NM_022455	1322	172	-21.29	3'UTR	mRNA
NUDT16L1	NM_032349	899	177	-25.69	3'UTR	mRNA
OPA1	NM_130834	2057	170	-24.03	5'UTR	mRNA
OXSR1	NM_005109	2977	177	-24.11	3'UTR	mRNA
PAPD5	NM_001040284	446	172	-20.96	3'UTR	mRNA
PCDH19	NM_020766	86	173	-23.34	3'UTR	mRNA
PCDHB9	NM_019119	37	170	-23.41	3'UTR	mRNA
PDHA1	NM_000284	1446	174	-25.10	3'UTR	mRNA
PGAP1	NM_024989	74	174	-26.64	CDS	mRNA
PIK3R3	NM_003629	2246	175	-21.38	3'UTR	mRNA
PLEKHA6	NM_014935	2289	173	-21.56	3'UTR	mRNA
PODXL	NM_005397	2416	170	-22.84	CDS	mRNA
PTBP2	NM_021190	184	172	-22.62	3'UTR	mRNA
PTCHD1	NM_173495	2	177	-21.12	CDS	mRNA
PXN	NM_025157	3046	172	-24.80	3'UTR	mRNA
RALGAPA1	NM_014990	370	171	-20.33	CDS	mRNA
RAPH1	NM_213589	1349	170	-24.60	3'UTR	mRNA
RBPJ	NM_005349	770	173	-21.14	3'UTR	mRNA
RIMS2	NM_014677	3	181	-23.16	3'UTR	mRNA
RNF144A	NM_014746	634	172	-30.05	3'UTR	mRNA
SAMD12	NM_001101676	973	179	-25.34	3'UTR	mRNA
SCAMP1	NM_004866	1426	174	-21.06	CDS	mRNA
SERF2	NM_001018108	8766	172	-22.76	3'UTR	mRNA
SFRP1	NM_003012	126	171	-30.20	3'UTR	mRNA
SH3TC2	NM_024577	21	176	-20.23	3'UTR	mRNA
SLC16A6	NM_004694	30	181	-30.91	CDS	mRNA
SLC19A2	NM_006996	1089	172	-23.16	CDS	mRNA
SLC22A15	NM_018420	58	174	-25.83	CDS	mRNA
SLC35F1	NM_001029858	16	171	-23.08	3'UTR	mRNA
SLC4A7	NM_003615	1619	181	-26.69	CDS	mRNA
SLCO3A1	NM_013272	213	178	-23.93	3'UTR	mRNA
SORBS1	NM_024991	1669	170	-22.76	CDS	mRNA
STRADA	NM_153335	590	172	-23.62	3'UTR	mRNA
SURF6	NM_006753	889	171	-24.63	3'UTR	mRNA
TBC1D15	NM_022771	808	177	-25.98	3'UTR	mRNA
THEMIS	NM_001010923	13	172	-22.81	3'UTR	mRNA
TLN2	NM_015059	868	174	-24.64	CDS	mRNA
TM2D2	NM_031940	1612	178	-21.99	3'UTR	mRNA
TMEM110	NM_198563	280	173	-20.87	3'UTR	mRNA
TMEM154	NM_152680	226	170	-22.72	3'UTR	mRNA

TOMM5	NM_001001790	1483	172	-28.82	3'UTR	mRNA
TRANK1	NM_014831	1217	170	-21.66	3'UTR	mRNA
TTBK2	NM_173500	58	178	-20.99	3'UTR	mRNA
TULP1	NM_003322	1	170	-26.27	CDS	mRNA
UBN1	NM_001079514	2657	175	-26.69	CDS	mRNA
UFM1	NM_016617	1113	172	-22.39	3'UTR	mRNA
VAPA	NM_003574	2656	175	-20.07	3'UTR	mRNA
VCAM1	NM_001078	1852	173	-24.07	3'UTR	mRNA
WEE1	NM_003390	1106	174	-25.16	CDS	mRNA
XRCC5	NM_021141	9600	178	-23.92	3'UTR	mRNA
ZBTB41	NM_194314	1345	172	-22.44	3'UTR	mRNA
ZDHHC21	NM_178566	111	176	-21.75	3'UTR	mRNA
ZEB2	NM_014795	643	179	-22.18	5'UTR	mRNA
ZHX3	NM_015035	538	174	-21.74	3'UTR	mRNA
ZNF140	NM_003440	675	179	-24.31	CDS	mRNA
ZNF273	NM_021148	230	180	-22.54	3'UTR	mRNA
ZNF460	NM_006635	29	170	-26.65	CDS	mRNA
ZNF502	NM_033210	121	170	-22.24	CDS	mRNA
ZNF527	NM_032453	82	170	-24.72	3'UTR	mRNA
ZNF763	NM_001012753	31	170	-21.19	CDS	mRNA
ZNF814	NM_001144989	203	172	-22.33	3'UTR	mRNA

Supplementary Table S6C: Putative DQ598183 piRNA target RNAs expressed in breast tumours. piRNAs targeting long non coding RNAs are shown in *italic*.

**Gene symbol**	**RefSeq n.er**	**Expression (normalized read count**	**Alignment score**	**Energy (kcal/mol)**	**DQ598183 binding in RNA**	**Transcript category**
GBAP1	NR_002188	1872	172	-31.55	-	ncRNA (pseudogene)
LOC646214	NR_027053	206	174	-26.87	-	ncRNA (pseudogene)
LOC100129550	*NR_024618*	*194*	*187*	*-30.54*	-	*lncRNA*
TSIX	*NR_003255*	*284*	*177*	*-30.05*	-	*lncRNA*
AASDH	NM_181806	347	171	-37.21	CDS	mRNA
ABL2	NM_005158	1446	171	-27.42	3'UTR	mRNA
ALDOC	NM_005165	77	171	-26.54	CDS	mRNA

AMHR2	NM_020547	2	170	-30.42	CDS	mRNA
AP3S2	NM_005829	746	172	-30.77	3'UTR	mRNA
ARCN1	NM_001655	3992	179	-23.49	3'UTR	mRNA
ARHGAP1	NM_004308	4399	172	-35.31	3'UTR	mRNA
ARHGAP20	NM_020809	49	170	-28.48	3'UTR	mRNA
ARID2	NM_152641	923	177	-25.63	3'UTR	mRNA
ATG4D	NM_032885	208	173	-31.79	CDS	mRNA
ATP1A4	NM_144699	19	170	-26.83	CDS	mRNA
AXIN2	NM_004655	184	171	-28.13	CDS	mRNA
BBS9	NM_014451	181	177	-30.36	CDS	mRNA
BSN	NM_003458	5	175	-22.95	CDS	mRNA
BTG2	NM_006763	6493	171	-33.84	3'UTR	mRNA
C1GALT1	NM_020156	149	170	-23.79	3'UTR	mRNA
CC2D1B	NM_032449	1149	175	-26.38	3'UTR	mRNA
CCL15	NM_032965	2	171	-28.62	5'UTR	mRNA
CCL18	NM_002988	202	171	-32.04	CDS	mRNA
CDC23	NM_004661	560	173	-28.93	3'UTR	mRNA
CHRDL1	NM_145234	29	174	-21.96	CDS	mRNA
CLEC2D	NM_013269	349	177	-28.28	3'UTR	mRNA
CNKSR2	NM_014927	1	172	-29.62	CDS	mRNA
CNOT4	NM_001190850	442	170	-33.37	CDS	mRNA
CPS1	NM_001875	67	172	-29.30	CDS	mRNA
CR1	NM_000651	13	172	-26.65	CDS	mRNA
CREBBP	NM_004380	3194	174	-30.10	CDS	mRNA
CRKL	NM_005207	2619	179	-27.49	3'UTR	mRNA
CXorf36	NM_176819	379	178	-41.35	3'UTR	mRNA
DARS2	NM_018122	2089	171	-26.49	CDS	mRNA
DAXX	NM_001350	1609	171	-30.38	CDS	mRNA
DCDC1	NM_181807	11	173	-24.80	CDS	mRNA
DDC	NM_001242890	233	177	-39.30	3'UTR	mRNA
DNAH10	NM_207437	5	172	-35.83	CDS	mRNA
DNAJB5	NM_012266	170	178	-37.10	3'UTR	mRNA
DTX3L	NM_138287	2514	181	-24.89	3'UTR	mRNA
DUSP8	NM_004420	119	170	-30.92	3'UTR	mRNA
EDA2R	NM_021783	45	173	-24.98	3'UTR	mRNA
EML4	NM_019063	2007	173	-31.33	3'UTR	mRNA
EMR2	NM_013447	75	174	-31.07	3'UTR	mRNA
FAM115C	NM_173678	225	170	-31.99	3'UTR	mRNA
FBXO44	NM_001014765	185	170	-38.75	5'UTR	mRNA
FKTN	NM_006731	641	170	-32.64	3'UTR	mRNA

FLNA	NM_001456	16130	176	-33.31	CDS	mRNA
FMN2	NM_020066	8	172	-34.97	CDS	mRNA
FOXRED2	NM_024955	1378	170	-28.35	3'UTR	mRNA
FRAS1	NM_025074	60	172	-24.49	3'UTR	mRNA
GALNT6	NM_007210	5873	170	-33.15	CDS	mRNA
GBA	NM_000157	6219	172	-31.55	CDS	mRNA
GGCX	NM_000821	1034	170	-23.93	3'UTR	mRNA
GLCCI1	NM_138426	169	173	-29.24	5'UTR	mRNA
GLS	NM_014905	737	172	-27.34	3'UTR	mRNA
H3F3B	NM_005324	11219	171	-25.74	3'UTR	mRNA
HDLBP	NM_005336	19002	173	-30.16	CDS	mRNA
HECTD3	NM_024602	965	170	-32.15	CDS	mRNA
HOMER1	NM_004272	145	171	-28.23	3'UTR	mRNA
IL22RA1	NM_021258	7	179	-39.02	CDS	mRNA
INTS8	NM_017864	1461	170	-30.18	5'UTR	mRNA
ISG20L2	NM_030980	1855	171	-25.53	3'UTR	mRNA
ITSN1	NM_003024	1117	171	-31.71	CDS	mRNA
KCNMA1	NM_001271520	797	171	-29.07	3'UTR	mRNA
KLRD1	NM_007334	39	172	-25.41	3'UTR	mRNA
KMO	NM_003679	887	178	-33.13	CDS	mRNA
LPPR4	NM_014839	35	176	-32.13	CDS	mRNA
LRRC3B	NM_052953	2	172	-30.71	3'UTR	mRNA
MAPK7	NM_002749	521	175	-40.33	CDS	mRNA
MAPKAPK3	NM_004635	452	171	-33.29	CDS	mRNA
MAPT	NM_005910	183	182	-30.39	3'UTR	mRNA
MED13	NM_005121	1654	170	-24.10	CDS	mRNA
METTL1	NM_023033	774	170	-33.78	CDS	mRNA
MFN1	NM_033540	901	172	-26.87	3'UTR	mRNA
MKL1	NM_020831	1101	170	-34.52	3'UTR	mRNA
MPI	NM_002435	662	172	-29.98	CDS	mRNA
MPI	NM_002435	662	172	-27.63	3'UTR	mRNA
MRAS	NM_012219	507	170	-29.50	3'UTR	mRNA
MRPS21	NM_018997	2251	178	-33.72	3'UTR	mRNA
MYBPC1	NM_002465	52	179	-29.54	CDS	mRNA
MYOCD	NM_153604	2	184	-40.00	CDS	mRNA
NCOA6	NM_014071	1963	171	-34.38	CDS	mRNA
NDFIP2	NM_019080	592	170	-32.46	5'UTR	mRNA
NID1	NM_002508	4112	171	-23.62	CDS	mRNA
NKD1	NM_033119	57	174	-34.89	3'UTR	mRNA
NOS3	NM_000603	180	170	-32.66	CDS	mRNA

OTUB2	NM_023112	199	171	-26.86	3'UTR	mRNA
PAK2	NM_002577	5212	174	-26.87	3'UTR	mRNA
PDIA6	NM_001282707	8810	170	-30.48	5'UTR	mRNA
PGR	NM_000926	54	172	-30.76	3'UTR	mRNA
PHKA1	NM_002637	534	177	-31.28	CDS	mRNA
PLK1	NM_005030	1123	178	-36.88	CDS	mRNA
PPAT	NM_002703	595	173	-31.12	CDS	mRNA
PRH2	NM_005042	1	172	-24.88	3'UTR	mRNA
PRKDC	NM_006904	9539	181	-41.98	CDS	mRNA
PSEN1	NM_000021	2269	172	-22.66	CDS	mRNA
PSMD1	NM_002807	3467	177	-30.80	CDS	mRNA
RAE1	NM_003610	875	170	-27.21	CDS	mRNA
RASSF8	NM_001164748	255	173	-29.50	3'UTR	mRNA
RAVER1	NM_133452	1470	177	-34.05	3'UTR	mRNA
REV3L	NM_002912	276	170	-31.98	CDS	mRNA
RNF111	NM_017610	521	176	-32.58	3'UTR	mRNA
RNPS1	NM_001286627	4599	177	-31.31	CDS	mRNA
RNPS1	NM_080594	4599	177	-31.31	3'UTR	mRNA
RRP12	NM_015179	1581	170	-31.06	CDS	mRNA
SCNN1B	NM_000336	1	177	-28.02	CDS	mRNA
SEC22A	NM_012430	393	170	-28.75	3'UTR	mRNA
SEL1L3	NM_015187	283	170	-29.36	CDS	mRNA
SHISA6	NM_207386	3	172	-28.43	3'UTR	mRNA
SIM2	NM_005069	26	174	-31.01	3'UTR	mRNA
SLC22A1	NM_153187	2	171	-45.37	CDS	mRNA
SLC27A1	NM_198580	557	173	-33.72	3'UTR	mRNA
SLC44A1	NM_080546	3988	171	-28.00	3'UTR	mRNA
SLC6A1	NM_003042	42	179	-29.81	CDS	mRNA
SLC6A8	NM_005629	1570	185	-34.96	3'UTR	mRNA
SLC9A2	NM_003048	121	175	-28.08	3'UTR	mRNA
SNX21	NM_033421	317	171	-30.92	3'UTR	mRNA
SORCS1	NM_052918	55	176	-29.52	3'UTR	mRNA
SPAG9	NM_001251971	1637	171	-28.00	5'UTR	mRNA
STAG2	NM_006603	2160	172	-27.51	CDS	mRNA
SYT11	NM_152280	389	170	-27.56	3'UTR	mRNA
TBL1XR1	NM_024665	3681	175	-34.49	5'UTR	mRNA
TENC1	NM_198316	636	172	-32.56	CDS	mRNA
TEX15	NM_031271	4	171	-24.77	CDS	mRNA
TFDP2	NM_006286	611	171	-32.03	CDS	mRNA
TNRC6A	NM_014494	1313	171	-23.71	CDS	mRNA

TRMT6	NM_015939	569	176	-29.84	CDS	mRNA
TTC21B	NM_024753	336	170	-29.99	3'UTR	mRNA
UBA52	NM_003333	8493	187	-50.67	3'UTR	mRNA
USH1G	NM_173477	7	171	-29.53	3'UTR	mRNA
VCAM1	NM_001078	1852	172	-32.02	CDS	mRNA
VPS13C	NM_017684	501	177	-24.82	CDS	mRNA
VWA5A	NM_014622	416	175	-29.14	CDS	mRNA
WDFY1	NM_020830	1348	171	-28.72	3'UTR	mRNA
WHSC1L1	NM_023034	1102	172	-31.32	CDS	mRNA
ZBED4	NM_014838	1382	171	-23.25	3'UTR	mRNA
ZC3H12A	NM_025079	166	180	-30.17	CDS	mRNA
ZMIZ1	NM_020338	3369	182	-33.86	5'UTR	mRNA
ZNF132	NM_003433	32	173	-26.88	CDS	mRNA
ZNF134	NM_003435	265	175	-29.86	3'UTR	mRNA
ZNF337	NM_015655	315	171	-27.89	CDS	mRNA
ZNF416	NM_017879	104	170	-26.90	CDS	mRNA
ZNF605	NM_183238	793	171	-22.42	3'UTR	mRNA
ZNF618	NM_133374	781	172	-31.18	3'UTR	mRNA
ZNF862	NM_001099220	194	174	-29.87	CDS	mRNA
ZYG11B	NM_024646	1493	174	-24.11	3'UTR	mRNA

Supplementary Table S6D: Putative DQ597341 piRNA target RNAs expressed in breast tumours. piRNAs targeting long non coding RNAs are shown in *italic*.

**Gene symbol**	**RefSeq n.er**	**Expression (normalized read count)**	**Alignment score**	**Energy (kcal/mol)**	**DQ597341 binding in RNA**	**Transcript category**
MST1P2	NR_027504	12	176	-40.47	-	ncRNA (pseudogene)
NAPSB	NR_002798	79	185	-49.98	-	ncRNA (pseudogene)
C1orf213	*NR_033690*	*31*	*183*	*-44.95*	-	*lncRNA*
LOC388692	*NR_027002*	*452*	*174*	*-36.82*	-	*lncRNA*
ABCA7	NM_019112	170	172	-40.03	CDS	mRNA
ABCB9	NM_019624	572	170	-33.38	CDS	mRNA
ABCC4	NM_005845	118	177	-40.38	CDS	mRNA
ABCC8	NM_000352	107	171	-32.00	CDS	mRNA
ADAM15	NM_003815	8249	174	-40.45	CDS	mRNA
ADAMTS1	NM_006988	914	173	-41.67	CDS	mRNA
ADAMTS7	NM_014272	368	170	-42.72	CDS	mRNA

AGAP2	NM_001122772	127	175	-35.54	CDS	mRNA
AK2	NM_001625	1938	171	-36.49	CDS	mRNA
ALKBH2	NM_001001655	386	172	-37.11	CDS	mRNA
ALPK3	NM_020778	144	174	-48.94	CDS	mRNA
ANKRD23	NM_144994	56	171	-32.18	CDS	mRNA
ANKRD39	NM_016466	181	184	-44.19	CDS	mRNA
ANKS4B	NM_145865	2	172	-37.41	CDS	mRNA
ANXA4	NM_001153	2198	177	-47.79	CDS	mRNA
ANXA6	NM_001155	3681	176	-38.09	CDS	mRNA
AP2B1	NM_001282	18854	178	-40.57	CDS	mRNA
APEH	NM_001640	1525	183	-43.77	CDS	mRNA
APLP2	NM_001642	18907	173	-41.31	CDS	mRNA
ARF1	NM_001024227	21030	172	-34.65	5'UTR	mRNA
ARHGEF11	NM_014784	1489	171	-41.50	CDS	mRNA
ARHGEF5	NM_005435	439	351	-73.53	CDS	mRNA
ARID1B	NM_017519	855	174	-40.98	CDS	mRNA
ASAP3	NM_017707	550	173	-41.70	CDS	mRNA
ASTN1	NM_004319	8	170	-40.22	CDS	mRNA
AUTS2	NM_015570	586	176	-43.06	CDS	mRNA
BAZ1B	NM_032408	2378	175	-32.82	CDS	mRNA
BAZ2A	NM_013449	2287	181	-36.39	CDS	mRNA
BBS1	NM_024649	535	349	-70.21	CDS	mRNA
BIRC5	NM_001168	418	200	-48.77	3'UTR	mRNA
BMP7	NM_001719	30	175	-38.68	CDS	mRNA
C15orf62	NM_001130448	20	173	-33.81	CDS	mRNA
C17orf107	NM_001145536	83	174	-40.27	CDS	mRNA
C1S	NM_001734	8100	174	-38.28	CDS	mRNA
C2CD4B	NM_001007595	169	172	-38.75	CDS	mRNA
CACNA1B	NM_000718	1	176	-37.13	CDS	mRNA
CARD10	NM_014550	272	171	-37.74	CDS	mRNA
CARD14	NM_024110	507	171	-33.87	CDS	mRNA
CARKD	NM_018210	848	170	-44.84	5'UTR	mRNA
CARKD	NM_001242882	848	170	-44.84	CDS	mRNA
CASKIN1	NM_020764	3	176	-39.52	CDS	mRNA
CATSPER1	NM_053054	2	351	-84.86	CDS	mRNA
CATSPER1	NM_053054	2	177	-46.95	3'UTR	mRNA
CC2D1A	NM_017721	812	172	-43.01	CDS	mRNA
CCDC22	NM_014008	489	172	-35.27	CDS	mRNA
CCDC78	NM_001031737	107	172	-37.22	CDS	mRNA
CDH23	NM_022124	23	176	-35.08	CDS	mRNA

CDH24	NM_022478	220	182	-46.58	CDS	mRNA
CDH3	NM_001793	1142	171	-38.83	5'UTR	mRNA
CDH4	NM_001794	2	190	-44.91	CDS	mRNA
CDKN2B	NM_004936	726	180	-37.95	5'UTR	mRNA
CHRM1	NM_000738	4	171	-36.58	3'UTR	mRNA
CHST2	NM_004267	89	175	-37.22	5'UTR	mRNA
CHTF18	NM_022092	417	170	-35.03	CDS	mRNA
CHTF8	NM_001040146	1663	178	-47.82	3'UTR	mRNA
CLEC16A	NM_015226	832	173	-33.08	CDS	mRNA
COG4	NM_015386	868	176	-37.28	3'UTR	mRNA
COL18A1	NM_030582	4383	174	-40.00	CDS	mRNA
COL5A2	NM_000393	19588	170	-32.66	CDS	mRNA
COL7A1	NM_000094	396	175	-33.44	CDS	mRNA
CORO1C	NM_014325	3585	172	-32.51	CDS	mRNA
CSMD1	NM_033225	120	176	-36.31	CDS	mRNA
CTNND2	NM_001332	8	172	-37.27	CDS	mRNA
CYB561D1	NM_182580	70	170	-35.45	3'UTR	mRNA
DEF8	NM_207514	302	176	-46.99	3'UTR	mRNA
DES	NM_001927	6	178	-41.55	CDS	mRNA
DGKI	NM_004717	12	183	-45.72	CDS	mRNA
DGKQ	NM_001347	291	181	-46.36	CDS	mRNA
DHCR7	NM_001360	2917	175	-31.18	5'UTR	mRNA
DHDDS	NM_024887	463	170	-35.71	CDS	mRNA
DHODH	NM_001361	104	172	-38.71	CDS	mRNA
DHRS12	NM_001270424	176	175	-36.25	CDS	mRNA
DHRS12	NM_024705	176	171	-39.26	3'UTR	mRNA
DHRS2	NM_005794	89	170	-34.66	CDS	mRNA
DHX58	NM_024119	431	171	-39.49	CDS	mRNA
DNAH3	NM_017539	19	176	-36.44	CDS	mRNA
DNAJC17	NM_018163	272	178	-34.61	CDS	mRNA
DNAJC7	NM_003315	1270	185	-41.08	5'UTR	mRNA
DNTTIP1	NM_052951	479	177	-42.90	CDS	mRNA
DPYSL2	NM_001386	1259	173	-31.35	CDS	mRNA
DPYSL4	NM_006426	40	179	-30.87	CDS	mRNA
DPYSL5	NM_020134	1	172	-42.46	CDS	mRNA
DRG2	NM_001388	1452	189	-45.05	CDS	mRNA
DSCAML1	NM_020693	2	171	-39.83	CDS	mRNA
ECT2L	NM_001077706	6	172	-32.20	CDS	mRNA
EDIL3	NM_005711	498	171	-45.59	5'UTR	mRNA
EFNB2	NM_004093	982	171	-38.30	3'UTR	mRNA

EIF1B	NM_005875	425	175	-37.72	5'UTR	mRNA
EIF2B2	NM_014239	527	179	-31.01	CDS	mRNA
ELF3	NM_004433	4323	171	-39.18	CDS	mRNA
ENY2	NM_020189	923	173	-49.93	5'UTR	mRNA
ERMP1	NM_024896	1945	178	-45.13	3'UTR	mRNA
EZR	NM_003379	4060	174	-35.16	CDS	mRNA
FAF1	NM_007051	512	178	-34.16	5'UTR	mRNA
FAM129C	NM_173544	1	173	-42.69	CDS	mRNA
FAM13B	NM_016603	356	176	-41.14	5'UTR	mRNA
FAM178B	NM_001122646	351	179	-35.53	CDS	mRNA
FAM65C	NM_001290268	20	171	-36.79	5'UTR	mRNA
FAM83A	NM_032899	957	176	-34.82	3'UTR	mRNA
FBXW9	NM_032301	329	186	-40.75	CDS	mRNA
FCHO1	NM_015122	17	172	-43.08	CDS	mRNA
FEN1	NM_004111	854	177	-33.96	CDS	mRNA
FES	NM_002005	123	182	-40.63	CDS	mRNA
FOSL1	NM_005438	18	180	-44.73	CDS	mRNA
FURIN	NM_002569	2933	172	-37.49	CDS	mRNA
G6PD	NM_000402	1949	177	-36.64	CDS	mRNA
GAB3	NM_080612	50	180	-36.16	CDS	mRNA
GFRA3	NM_001496	6	173	-42.96	3'UTR	mRNA
GLE1	NM_001499	1003	177	-42.52	CDS	mRNA
GMIP	NM_016573	589	179	-42.51	CDS	mRNA
GNAO1	NM_138736	10	174	-34.27	3'UTR	mRNA
GNAZ	NM_002073	97	178	-39.27	CDS	mRNA
GOPC	NM_020399	864	177	-35.22	CDS	mRNA
GPR108	NM_001080452	901	178	-35.16	CDS	mRNA
GPR162	NM_019858	12	170	-32.80	CDS	mRNA
GPR89A	NM_001097613	1732	175	-37.17	CDS	mRNA
GPR89B	NM_016334	22	175	-37.17	CDS	mRNA
GPR89C	NM_001097616	85	175	-37.17	CDS	mRNA
GRIA4	NM_000829	12	175	-36.46	5'UTR	mRNA
GRINA	NM_000837	7013	170	-35.23	CDS	mRNA
GRN	NM_002087	7914	173	-37.23	CDS	mRNA
GTPBP3	NM_133644	255	179	-37.23	3'UTR	mRNA
GUCY2D	NM_000180	2	182	-42.74	CDS	mRNA
HCN4	NM_005477	1	171	-40.72	CDS	mRNA
HEPH	NM_138737	923	342	-80.28	CDS	mRNA
HIC2	NM_015094	105	175	-46.92	3'UTR	mRNA
HRH3	NM_007232	2	192	-40.53	CDS	mRNA

IL16	NM_172217	307	170	-39.19	CDS	mRNA
IL7	NM_000880	15	172	-39.09	5'UTR	mRNA
INSR	NM_000208	2400	178	-36.55	CDS	mRNA
IRAK2	NM_001570	25	170	-36.18	CDS	mRNA
ITGA3	NM_005501	1150	170	-32.47	CDS	mRNA
ITGA3	NM_002204	1150	170	-32.47	3'UTR	mRNA
ITGB2	NM_000211	1310	171	-38.85	CDS	mRNA
ITPK1	NM_014216	1786	173	-42.39	CDS	mRNA
IWS1	NM_017969	1811	170	-29.49	5'UTR	mRNA
KANK3	NM_198471	48	173	-41.10	CDS	mRNA
KCNJ5	NM_000890	74	177	-41.02	CDS	mRNA
KHSRP	NM_003685	2540	172	-36.28	CDS	mRNA
KIAA1109	NM_015312	1023	171	-36.25	CDS	mRNA
KIAA1244	NM_020340	1848	171	-41.34	CDS	mRNA
KIAA1614	NM_020950	25	170	-38.41	CDS	mRNA
KIAA1683	NM_001145304	10	174	-35.26	CDS	mRNA
KIAA1683	NM_025249	10	174	-35.26	CDS	mRNA
KIF19	NM_153209	3	171	-33.50	CDS	mRNA
KIF21B	NM_017596	165	174	-38.69	CDS	mRNA
KIF7	NM_198525	108	172	-34.19	CDS	mRNA
KLHL2	NM_007246	574	172	-38.15	CDS	mRNA
KRT18	NM_199187	9021	348	-69.79	CDS	mRNA
LDB3	NM_007078	14	170	-37.16	CDS	mRNA
LEF1	NM_016269	293	171	-28.70	5'UTR	mRNA
LEPREL1	NM_018192	57	170	-34.37	3'UTR	mRNA
LGI2	NM_018176	66	188	-33.62	CDS	mRNA
LIMK2	NM_001031801	1735	171	-38.66	CDS	mRNA
LMNB2	NM_032737	1572	171	-41.68	CDS	mRNA
LMOD1	NM_012134	895	176	-35.17	CDS	mRNA
LONP1	NM_004793	1948	179	-32.79	CDS	mRNA
LRP1	NM_002332	9336	170	-37.12	CDS	mRNA
LRRC32	NM_005512	749	173	-29.57	3'UTR	mRNA
LSS	NM_002340	546	170	-37.05	CDS	mRNA
MACF1	NM_012090	6051	173	-38.66	CDS	mRNA
MAGEC1	NM_005462	2	177	-44.68	CDS	mRNA
MAP3K11	NM_002419	1208	178	-46.46	3'UTR	mRNA
MBTD1	NM_017643	553	172	-39.11	5'UTR	mRNA
MCC	NM_002387	646	173	-33.52	CDS	mRNA
MED23	NM_004830	854	177	-37.66	5'UTR	mRNA
MLF2	NM_005439	4755	174	-38.17	CDS	mRNA

MMRN2	NM_024756	630	171	-34.11	CDS	mRNA
MN1	NM_002430	239	176	-45.82	CDS	mRNA
MRPL2	NM_015950	385	173	-37.94	CDS	mRNA
MSRA	NM_012331	62	171	-35.71	CDS	mRNA
MST1	NM_020998	11	172	-37.83	CDS	mRNA
MYH10	NM_005964	1800	171	-33.95	CDS	mRNA
MYOF	NM_013451	2684	171	-30.88	CDS	mRNA
NAGPA	NM_016256	255	170	-41.09	3'UTR	mRNA
NAP1L4	NM_005969	2493	177	-35.31	CDS	mRNA
NAPSA	NM_004851	2	189	-49.46	CDS	mRNA
NHLRC4	NM_176677	738	172	-36.59	5'UTR	mRNA
NHSL1	NM_020464	546	171	-32.15	CDS	mRNA
NKX2-3	NM_145285	5	174	-43.74	CDS	mRNA
NLGN3	NM_001166660	173	173	-36.37	CDS	mRNA
NOTCH2	NM_024408	8797	177	-32.43	3'UTR	mRNA
NPAS1	NM_002517	6	170	-37.47	CDS	mRNA
NPR2	NM_003995	178	170	-39.08	CDS	mRNA
NR4A2	NM_006186	811	177	-35.21	CDS	mRNA
NRBP2	NM_178564	208	170	-42.85	CDS	mRNA
NRG3	NM_001010848	3	176	-42.21	CDS	mRNA
NRXN2	NM_015080	47	178	-42.24	CDS	mRNA
NRXN3	NM_001105250	27	173	-35.00	CDS	mRNA
NUP210	NM_024923	1003	181	-37.26	CDS	mRNA
OLFML2B	NM_015441	1297	182	-43.44	CDS	mRNA
OR51E1	NM_152430	47	170	-27.83	CDS	mRNA
OXSM	NM_017897	630	172	-38.14	5'UTR	mRNA
P2RY2	NM_002564	136	170	-37.93	3'UTR	mRNA
PAK7	NM_020341	1	181	-37.68	CDS	mRNA
PARP9	NM_031458	3840	177	-38.29	CDS	mRNA
PCDH19	NM_020766	86	173	-37.44	CDS	mRNA
PCNXL2	NM_014801	111	175	-36.55	CDS	mRNA
PDE4C	NM_001098819	4	174	-30.61	3'UTR	mRNA
PDE4D	NM_006203	113	170	-39.72	CDS	mRNA
PDE4DIP	NM_014644	941	172	-40.86	3'UTR	mRNA
PDLIM7	NM_203352	1105	179	-39.84	CDS	mRNA
PDPK1	NM_002613	1151	170	-38.75	3'UTR	mRNA
PFDN1	NM_002622	875	170	-41.68	CDS	mRNA
PGAM5	NM_138575	912	179	-37.05	3'UTR	mRNA
PGC	NM_001166424	59	172	-34.83	CDS	mRNA
PHF20	NM_016436	1619	176	-34.96	CDS	mRNA

PINK1	NM_032409	875	173	-34.69	CDS	mRNA
PITPNM2	NM_020845	144	170	-30.40	CDS	mRNA
PLA2G4C	NM_003706	52	171	-35.16	CDS	mRNA
PLCD1	NM_006225	610	349	-73.52	CDS	mRNA
PLEKHA5	NM_001256470	435	175	-35.37	CDS	mRNA
PLXNA1	NM_032242	1379	171	-39.13	3'UTR	mRNA
PNPLA6	NM_006702	845	182	-37.19	CDS	mRNA
PPM1H	NM_020700	1011	179	-37.78	3'UTR	mRNA
PPP1R3B	NM_024607	2106	179	-37.51	CDS	mRNA
PPP4R1	NM_005134	1430	182	-37.90	CDS	mRNA
PSAT1	NM_058179	431	170	-36.42	CDS	mRNA
PSMC4	NM_153001	1617	170	-33.59	CDS	mRNA
PTCHD2	NM_020780	6	175	-37.05	CDS	mRNA
PTPRB	NM_002837	541	170	-35.60	CDS	mRNA
RAB20	NM_017817	539	180	-35.39	5'UTR	mRNA
RAB34	NM_031934	438	170	-44.95	5'UTR	mRNA
RCAN3	NM_001251979	105	177	-34.00	5'UTR	mRNA
RCOR1	NM_015156	1153	173	-32.07	3'UTR	mRNA
REM2	NM_173527	4	171	-40.70	CDS	mRNA
RNF20	NM_019592	1741	170	-32.99	CDS	mRNA
RNF214	NM_207343	218	176	-41.76	CDS	mRNA
RNF31	NM_017999	567	189	-40.88	CDS	mRNA
RPS29	NM_001032	6592	178	-35.62	3'UTR	mRNA
RTL1	NM_001134888	21	170	-38.31	CDS	mRNA
RTN1	NM_021136	59	176	-38.52	CDS	mRNA
RYR3	NM_001036	14	178	-42.53	CDS	mRNA
SDC3	NM_014654	2085	170	-35.15	CDS	mRNA
SDK2	NM_001144952	72	171	-41.72	3'UTR	mRNA
SDPR	NM_004657	85	178	-40.96	CDS	mRNA
SEC14L5	NM_014692	2	180	-39.37	3'UTR	mRNA
SEC24D	NM_014822	2512	189	-41.86	CDS	mRNA
SELP	NM_003005	3	177	-36.84	CDS	mRNA
SEP1	NM_052838	53	171	-34.77	CDS	mRNA
SERINC2	NM_018565	2031	179	-46.07	5'UTR	mRNA
SGSM1	NM_001098497	23	171	-37.22	CDS	mRNA
SHANK1	NM_016148	4	173	-37.22	CDS	mRNA
SIT1	NM_014450	30	170	-39.05	CDS	mRNA
SLC12A9	NM_020246	633	170	-37.03	CDS	mRNA
SLC23A1	NM_005847	18	172	-40.11	CDS	mRNA
SLC25A39	NM_016016	2572	173	-39.22	CDS	mRNA

SLC4A8	NM_001039960	406	171	-34.72	3'UTR	mRNA
SLC9A3	NM_004174	1	174	-32.37	CDS	mRNA
SLFN14	NM_001129820	1	178	-32.02	CDS	mRNA
SMTN	NM_006932	703	172	-33.28	CDS	mRNA
SPEF1	NM_015417	65	178	-42.14	3'UTR	mRNA
SPRY2	NM_005842	96	172	-40.27	CDS	mRNA
SRPX2	NM_014467	561	178	-44.91	CDS	mRNA
ST5	NM_005418	1357	171	-39.31	CDS	mRNA
STK10	NM_005990	921	171	-35.36	3'UTR	mRNA
SURF1	NM_003172	855	175	-44.13	CDS	mRNA
SYNPO	NM_001109974	1293	172	-36.20	3'UTR	mRNA
TBC1D12	NM_015188	241	171	-33.06	CDS	mRNA
TFEB	NM_007162	176	170	-40.00	CDS	mRNA
TINAGL1	NM_022164	192	170	-38.76	CDS	mRNA
TLX1	NM_005521	4	175	-40.41	CDS	mRNA
TMEM104	NM_017728	500	177	-37.04	CDS	mRNA
TMEM130	NM_152913	139	174	-42.00	CDS	mRNA
TMEM44	NM_138399	152	175	-42.64	CDS	mRNA
TMEM50B	NM_006134	692	190	-35.08	CDS	mRNA
TNF	NM_000594	6	173	-28.55	CDS	mRNA
TNFRSF21	NM_014452	789	177	-33.67	CDS	mRNA
TNK2	NM_005781	1823	179	-34.69	CDS	mRNA
TRAM1L1	NM_152402	98	172	-36.30	5'UTR	mRNA
TRH	NM_007117	1	174	-37.35	CDS	mRNA
TRIM29	NM_012101	274	173	-37.29	CDS	mRNA
TRIM3	NM_001248006	454	172	-34.90	5'UTR	mRNA
TRIM39	NM_021253	338	175	-39.54	CDS	mRNA
TRIM45	NM_025188	118	171	-32.76	5'UTR	mRNA
TRO	NM_016157	88	172	-42.13	CDS	mRNA
TROVE2	NM_001173524	1421	173	-40.21	5'UTR	mRNA
TRPT1	NM_031472	297	172	-42.29	CDS	mRNA
TRRAP	NM_003496	2268	171	-38.27	CDS	mRNA
TSPYL2	NM_022117	343	170	-34.64	CDS	mRNA
TTBK1	NM_032538	65	170	-39.93	5'UTR	mRNA
TYK2	NM_003331	1012	190	-39.61	CDS	mRNA
UBQLN4	NM_020131	3763	170	-36.27	3'UTR	mRNA
UBXN10	NM_152376	207	179	-36.86	CDS	mRNA
UNC13B	NM_006377	683	178	-34.49	CDS	mRNA
USP18	NM_017414	712	170	-34.50	CDS	mRNA
VCL	NM_003373	4249	170	-38.34	CDS	mRNA

VSIG10L	NM_001163922	46	188	-42.91	CDS	mRNA
VWA2	NM_001272046	221	172	-39.14	CDS	mRNA
WARS2	NM_015836	686	175	-39.43	CDS	mRNA
WDR81	NM_152348	657	172	-49.95	CDS	mRNA
XIRP1	NM_194293	54	192	-43.83	CDS	mRNA
ZBTB22	NM_005453	409	173	-39.92	CDS	mRNA
ZBTB7C	NM_001039360	242	191	-50.58	CDS	mRNA
ZC3H7B	NM_017590	1926	174	-37.33	CDS	mRNA
ZKSCAN1	NM_003439	964	170	-34.27	3'UTR	mRNA
ZNF541	NM_001277075	18	170	-33.29	CDS	mRNA
ZNF592	NM_014630	627	181	-42.82	CDS	mRNA
ZNF750	NM_024702	66	172	-35.83	CDS	mRNA

Supplementary Table S6E: Putative DQ598252 piRNA target RNAs expressed in breast tumours.

Gene symbol	RefSeq n.er	Expression (normalized read count)	Alignment score	Energy (kcal/mol)	DQ598252 binding in RNA	Transcript category
ARF3	NM_001659	7125	174	-26.14	CDS	mRNA
BDH2	NM_020139	386	170	-34.55	3'UTR	mRNA
DNAH14	NM_001373	172	174	-24.77	CDS	mRNA
DOT1L	NM_032482	527	173	-32.12	CDS	mRNA
ESPNL	NM_194312	1	172	-39.39	CDS	mRNA
HECW1	NM_015052	15	181	-33.26	CDS	mRNA
ICAM5	NM_003259	3	170	-33.54	CDS	mRNA
IGSF10	NM_178822	59	173	-31.48	CDS	mRNA
IL34	NM_152456	87	171	-33.60	5'UTR	mRNA
MAST1	NM_014975	3	171	-28.58	CDS	mRNA
MTOR	NM_004958	1223	170	-25.73	CDS	mRNA
NDUFV3	NM_021075	747	174	-29.06	CDS	mRNA
NID2	NM_007361	1526	174	-28.95	CDS	mRNA
PLXNA1	NM_032242	1379	175	-36.31	CDS	mRNA
QARS	NM_005051	2817	176	-31.41	CDS	mRNA
RAB7L1	NM_003929	1703	176	-31.40	CDS	mRNA
SORBS1	NM_006434	1669	174	-35.13	CDS	mRNA
SORT1	NM_002959	2108	171	-36.03	CDS	mRNA
TMEM229B	NM_182526	180	178	-30.28	3'UTR	mRNA
TTC19	NM_017775	673	173	-30.98	5'UTR	mRNA

TTC37	NM_014639	1044	171	-28.83	CDS	mRNA
U2AF1	NM_001025204	1279	175	-37.18	5'UTR	mRNA
ZNF609	NM_015042	1759	171	-34.68	CDS	mRNA

Supplementary Table S6F: Putative DQ596311 piRNA target RNAs expressed in breast tumours. piRNAs targeting long non coding RNAs are shown in *italic*.

**Gene symbol**	**RefSeq n.er**	**Expression (normalized read count)**	**Alignment score**	**Energy (kcal/mol)**	**DQ596311 binding in RNA**	**Transcript category**
CCDC144B	NR_036647	43	170	-25.49	-	ncRNA (pseudogene)
HSP90AB4P	NR_073415	3	184	-27.27	-	ncRNA (pseudogene)
LOC154761	NR_015421	44	175	-25.81	-	ncRNA (pseudogene)
LOC493754	NR_002933	274	182	-29.12	-	ncRNA (pseudogene)
CASC2	*NR_026939*	*33*	*175*	*-23.13*	-	*lncRNA*
FLJ41941	*NR_024417*	*2*	*175*	*-20.81*	-	*lncRNA*
ABCB11	NM_003742	1	174	-25.70	CDS	mRNA
ABCB6	NM_005689	1135	173	-39.79	CDS	mRNA
ABCC1	NM_004996	2573	172	-28.57	CDS	mRNA
ABCD2	NM_005164	4	174	-32.16	CDS	mRNA
ABI1	NM_005470	1190	182	-26.14	3'UTR	mRNA
ACN9	NM_020186	187	172	-27.76	3'UTR	mRNA
ACO2	NM_001098	3053	173	-24.02	CDS	mRNA
ACTC1	NM_005159	4	186	-35.94	CDS	mRNA
ADAM10	NM_001110	1757	175	-27.56	3'UTR	mRNA
ADAMTS14	NM_080722	96	175	-36.06	CDS	mRNA
ADCY10	NM_018417	10	179	-26.99	CDS	mRNA
ADRA1A	NM_033303	3	172	-30.26	3'UTR	mRNA
AFF1	NM_005935	1702	172	-26.41	3'UTR	mRNA
AGL	NM_000644	7645	171	-28.07	5'UTR	mRNA
AGPS	NM_003659	554	176	-27.96	3'UTR	mRNA
AKNA	NM_030767	500	170	-27.08	3'UTR	mRNA
AKR1C1	NM_001353	12	173	-27.85	5'UTR	mRNA

AKR1C2	NM_001354	11	173	-27.85	5'UTR	mRNA
AKT3	NM_005465	491	172	-28.18	3'UTR	mRNA
ALDH6A1	NM_001278594	730	352	-53.67	5'UTR	mRNA
ALDH6A1	NM_005589	730	352	-53.67	CDS	mRNA
ALPK3	NM_020778	144	174	-26.98	3'UTR	mRNA
AMZ1	NM_133463	19	174	-24.94	3'UTR	mRNA
ANAPC16	NM_173473	1568	188	-28.51	3'UTR	mRNA
ANGPT2	NM_001147	666	173	-25.50	3'UTR	mRNA
APLNR	NM_005161	590	183	-29.73	3'UTR	mRNA
ARF4	NM_001660	5185	178	-26.84	CDS	mRNA
ARHGAP11A	NM_199357	284	180	-26.87	CDS	mRNA
ARHGAP29	NM_004815	692	170	-26.91	CDS	mRNA
ARHGEF12	NM_015313	3635	174	-26.05	3'UTR	mRNA
ARHGEF38	NM_001242729	51	178	-28.79	CDS	mRNA
ARMC8	NM_015396	570	170	-24.13	3'UTR	mRNA
ARMCX5	NM_022838	374	170	-25.45	CDS	mRNA
ASAP2	NM_003887	839	176	-32.64	3'UTR	mRNA
ATP10D	NM_020453	255	173	-23.02	CDS	mRNA
ATP12A	NM_001676	1	179	-33.31	CDS	mRNA
ATP5G3	NM_001689	2761	179	-28.94	CDS	mRNA
ATP9A	NM_006045	1853	177	-22.59	3'UTR	mRNA
ATXN7L3B	NM_001136262	4150	170	-22.58	3'UTR	mRNA
B3GNTL1	NM_001009905	94	184	-37.94	CDS	mRNA
BCKDK	NM_005881	2679	170	-29.82	CDS	mRNA
BCL11B	NM_022898	59	173	-30.57	3'UTR	mRNA
BCL2L11	NM_207002	1016	173	-20.32	3'UTR	mRNA
BTN3A2	NM_007047	1415	174	-25.31	3'UTR	mRNA
BTN3A3	NM_006994	612	173	-26.69	3'UTR	mRNA
C14orf28	NM_001017923	61	187	-33.77	CDS	mRNA
C17orf80	NM_017941	533	172	-24.33	3'UTR	mRNA
C1GALT1C1	NM_152692	675	170	-25.28	CDS	mRNA
C1orf210	NM_182517	120	174	-36.51	3'UTR	mRNA
C1S	NM_201442	8100	171	-22.84	CDS	mRNA
C9orf114	NM_016390	383	177	-29.71	3'UTR	mRNA
CACNA1E	NM_000721	2	172	-23.74	CDS	mRNA
CALCOCO1	NM_020898	936	183	-32.44	3'UTR	mRNA
CAMK2A	NM_171825	6	174	-25.29	3'UTR	mRNA
CBWD5	NM_001286835	2	170	-20.72	5'UTR	mRNA
CBX5	NM_012117	3027	174	-22.98	3'UTR	mRNA
CCNL1	NM_020307	703	178	-32.73	CDS	mRNA

CCNL2	NM_030937	759	177	-31.37	CDS	mRNA
CD302	NM_014880	452	188	-27.44	CDS	mRNA
CD96	NM_005816	115	176	-25.20	3'UTR	mRNA
CELSR3	NM_001407	425	172	-42.71	CDS	mRNA
CEP350	NM_014810	1802	175	-27.01	CDS	mRNA
CHURC1	NM_145165	1130	179	-30.41	3'UTR	mRNA
CLDN10	NM_182848	8	179	-28.18	5'UTR	mRNA
CLDN14	NM_144492	15	172	-26.06	CDS	mRNA
CLDN19	NM_148960	7	173	-31.64	3'UTR	mRNA
CLMN	NM_024734	1020	170	-28.25	3'UTR	mRNA
CLN5	NM_006493	632	172	-32.24	3'UTR	mRNA
CNOT7	NM_013354	1988	170	-24.44	3'UTR	mRNA
CNP	NM_033133	2231	170	-28.24	3'UTR	mRNA
CNTNAP3	NM_033655	5	173	-27.70	CDS	mRNA
COCH	NM_004086	10	172	-26.00	CDS	mRNA
COL13A1	NM_080800	65	177	-31.48	CDS	mRNA
COL9A3	NM_001853	54	173	-34.17	CDS	mRNA
CPA4	NM_016352	140	179	-21.39	CDS	mRNA
CPD	NM_001304	17929	173	-26.75	3'UTR	mRNA
CPEB1	NM_030594	4	170	-21.88	CDS	mRNA
CRY2	NM_021117	506	189	-33.05	CDS	mRNA
CSGALNACT2	NM_018590	802	173	-24.24	CDS	mRNA
CSPG4	NM_001897	1005	172	-26.53	CDS	mRNA
CYTH4	NM_013385	287	173	-30.48	3'UTR	mRNA
DAAM1	NM_014992	519	175	-24.41	3'UTR	mRNA
DBH	NM_000787	1	178	-28.78	CDS	mRNA
DDI2	NM_032341	109	170	-28.37	3'UTR	mRNA
DDX10	NM_004398	776	187	-34.96	CDS	mRNA
DDX23	NM_004818	3837	170	-28.05	CDS	mRNA
DFFB	NM_001282669	79	172	-23.40	CDS	mRNA
DHTKD1	NM_018706	2650	175	-20.79	CDS	mRNA
DHX33	NM_020162	382	170	-26.30	3'UTR	mRNA
DNAJC14	NM_032364	1489	171	-26.59	CDS	mRNA
DNAJC18	NM_152686	80	192	-33.77	3'UTR	mRNA
DOC2A	NM_003586	7	172	-24.47	CDS	mRNA
DR1	NM_001938	821	171	-21.55	3'UTR	mRNA
DTNA	NM_032975	22	171	-29.12	3'UTR	mRNA
EFR3A	NM_015137	6167	173	-24.70	3'UTR	mRNA
EGFL6	NM_015507	181	171	-23.03	CDS	mRNA
EIF1AX	NM_001412	2080	173	-21.78	3'UTR	mRNA

EIF2B2	NM_014239	527	181	-30.77	CDS	mRNA
ELK4	NM_001973	231	170	-23.84	CDS	mRNA
ELTD1	NM_022159	563	181	-28.91	CDS	mRNA
ERAP1	NM_016442	1153	176	-24.77	CDS	mRNA
ESR1	NM_000125	257	170	-30.83	3'UTR	mRNA
ESYT1	NM_015292	2687	172	-33.52	CDS	mRNA
FAM102A	NM_203305	1802	174	-22.44	5'UTR	mRNA
FAM102A	NM_001035254	1802	174	-23.58	CDS	mRNA
FAM115C	NM_001130026	225	178	-24.97	3'UTR	mRNA
FAM126A	NM_032581	226	174	-31.33	3'UTR	mRNA
FAM135A	NM_020819	76	170	-25.42	CDS	mRNA
FAM160A1	NM_001109977	108	182	-27.97	CDS	mRNA
FAM160B1	NM_020940	633	177	-27.57	3'UTR	mRNA
FAM76B	NM_144664	240	177	-26.71	3'UTR	mRNA
FAM98B	NM_173611	291	175	-24.55	3'UTR	mRNA
FAT3	NM_001008781	38	170	-28.39	CDS	mRNA
FBXO9	NM_012347	526	183	-30.46	3'UTR	mRNA
FBXW2	NM_012164	2135	171	-26.51	3'UTR	mRNA
FERMT1	NM_017671	10	182	-33.85	3'UTR	mRNA
FGD4	NM_139241	124	177	-27.08	3'UTR	mRNA
FGF12	NM_004113	14	175	-25.93	3'UTR	mRNA
FHDC1	NM_033393	275	171	-36.92	CDS	mRNA
FKBP11	NM_001143782	484	173	-24.43	3'UTR	mRNA
FLJ44635	NM_207422	19	171	-22.81	CDS	mRNA
FLRT3	NM_198391	349	172	-23.94	CDS	mRNA
FOXD2	NM_004474	45	171	-20.15	3'UTR	mRNA
FPR1	NM_002029	119	177	-33.99	CDS	mRNA
FREM1	NM_144966	20	173	-24.83	CDS	mRNA
FSTL1	NM_007085	9905	180	-25.75	3'UTR	mRNA
FTO	NM_001080432	1107	177	-25.40	3'UTR	mRNA
G3BP1	NM_005754	2638	170	-29.38	CDS	mRNA
GAPVD1	NM_001282679	1052	177	-30.71	CDS	mRNA
GDA	NM_004293	1	170	-30.61	3'UTR	mRNA
GNL1	NM_005275	918	170	-25.71	3'UTR	mRNA
GPR126	NM_020455	325	183	-23.77	CDS	mRNA
GPR173	NM_018969	9	178	-30.44	3'UTR	mRNA
GPR97	NM_170776	5	171	-25.93	CDS	mRNA
GPR98	NM_032119	62	174	-39.14	CDS	mRNA
GPRIN3	NM_198281	79	176	-28.33	3'UTR	mRNA
GPX7	NM_015696	248	175	-29.25	3'UTR	mRNA

GRHL1	NM_198182	1345	173	-21.13	3'UTR	mRNA
GRIA1	NM_000827	4	175	-32.57	CDS	mRNA
GRIK3	NM_000831	15	179	-26.73	CDS	mRNA
GRSF1	NM_002092	2947	190	-29.19	3'UTR	mRNA
GUCY2D	NM_000180	2	196	-30.65	CDS	mRNA
HARS2	NM_012208	617	170	-29.73	CDS	mRNA
HDAC10	NM_032019	370	174	-24.50	CDS	mRNA
HEXB	NM_000521	2140	170	-22.83	CDS	mRNA
HGF	NM_000601	50	181	-24.54	CDS	mRNA
HIP1	NM_005338	2169	171	-31.05	3'UTR	mRNA
HK2	NM_000189	5144	180	-27.93	5'UTR	mRNA
HMCN1	NM_031935	1352	172	-33.33	CDS	mRNA
HS2ST1	NM_012262	1307	172	-21.61	3'UTR	mRNA
HSDL2	NM_032303	1093	178	-27.75	3'UTR	mRNA
IKZF2	NM_016260	57	171	-26.58	3'UTR	mRNA
IL13RA1	NM_001560	8494	174	-21.13	3'UTR	mRNA
IL17REL	NM_001001694	4	170	-27.79	3'UTR	mRNA
INADL	NM_176877	2283	170	-22.55	3'UTR	mRNA
INPP5B	NM_005540	330	171	-21.65	3'UTR	mRNA
INPP5F	NM_014937	549	179	-30.41	CDS	mRNA
INTS3	NM_023015	3484	179	-36.62	CDS	mRNA
INTS8	NM_017864	1461	170	-26.05	CDS	mRNA
IRAK1	NM_001569	2281	172	-22.09	3'UTR	mRNA
ITGAV	NM_002210	4875	171	-28.76	CDS	mRNA
ITGB5	NM_002213	11279	175	-33.35	CDS	mRNA
ITPRIPL1	NM_001008949	11	188	-33.29	CDS	mRNA
JARID2	NM_004973	1028	174	-29.48	5'UTR	mRNA
JKAMP	NM_016475	769	177	-27.05	CDS	mRNA
KCTD3	NM_016121	3494	173	-31.33	3'UTR	mRNA
KIAA0196	NM_014846	1437	175	-29.39	CDS	mRNA
KIAA0556	NM_015202	1123	170	-26.73	CDS	mRNA
KIF24	NM_194313	266	170	-21.62	3'UTR	mRNA
KLF13	NM_015995	642	170	-30.58	3'UTR	mRNA
KLHL12	NM_021633	1587	171	-34.71	CDS	mRNA
KLHL23	NM_144711	84	176	-24.40	CDS	mRNA
KLK2	NM_005551	3	177	-22.48	3'UTR	mRNA
KRAS	NM_004985	1062	175	-26.62	3'UTR	mRNA
KRT80	NM_182507	137	172	-25.47	3'UTR	mRNA
LAG3	NM_002286	77	170	-36.19	CDS	mRNA
LDLRAD3	NM_174902	465	180	-31.70	CDS	mRNA

LIPG	NM_006033	65	171	-26.96	CDS	mRNA
LPGAT1	NM_014873	4515	172	-30.03	3'UTR	mRNA
LPIN2	NM_014646	1574	170	-25.41	3'UTR	mRNA
LRP2	NM_004525	444	342	-52.99	CDS	mRNA
LRRC4	NM_022143	27	172	-30.94	CDS	mRNA
LRRC59	NM_018509	5725	180	-30.28	3'UTR	mRNA
LSM2	NM_021177	830	170	-26.93	3'UTR	mRNA
MACF1	NM_012090	6051	175	-32.40	CDS	mRNA
MAGED1	NM_006986	6905	185	-38.98	CDS	mRNA
MAGI1	NM_004742	273	176	-28.29	CDS	mRNA
MAN1A1	NM_005907	1475	179	-22.79	3'UTR	mRNA
MAST3	NM_015016	269	171	-30.76	CDS	mRNA
MATN2	NM_002380	88	362	-63.42	CDS	mRNA
MCM4	NM_005914	4190	173	-26.66	3'UTR	mRNA
MERTK	NM_006343	133	176	-23.48	CDS	mRNA
MGAT4A	NM_012214	3732	177	-30.61	3'UTR	mRNA
MGRN1	NM_015246	2091	172	-30.25	3'UTR	mRNA
MORN4	NM_178832	118	171	-29.63	3'UTR	mRNA
MPZ	NM_000530	8	174	-23.87	3'UTR	mRNA
MR1	NM_001531	349	170	-21.31	3'UTR	mRNA
MTA3	NM_020744	1918	180	-32.64	CDS	mRNA
MTFR1	NM_014637	1710	171	-25.57	3'UTR	mRNA
MTX3	NM_001167741	346	171	-23.53	3'UTR	mRNA
MUC12	NM_001164462	1	182	-31.37	CDS	mRNA
MUC16	NM_024690	13	347	-55.17	CDS	mRNA
MYB	NM_005375	1847	172	-24.53	CDS	mRNA
MYBPC1	NM_002465	52	174	-29.90	CDS	mRNA
MYO1D	NM_015194	2754	172	-27.61	CDS	mRNA
NAA25	NM_024953	908	171	-28.03	3'UTR	mRNA
NAV3	NM_014903	88	345	-47.87	CDS	mRNA
NCDN	NM_014284	924	170	-23.14	3'UTR	mRNA
NCKAP1	NM_205842	3531	172	-25.62	3'UTR	mRNA
NDUFA4	NM_002489	2807	172	-22.44	3'UTR	mRNA
NDUFB5	NM_002492	1451	170	-22.55	CDS	mRNA
NOTCH2	NM_024408	8797	175	-21.62	CDS	mRNA
NR1D2	NM_005126	613	182	-29.34	3'UTR	mRNA
NT5C2	NM_012229	1043	186	-30.90	3'UTR	mRNA
NUCKS1	NM_022731	13941	172	-25.29	3'UTR	mRNA
NUDT10	NM_153183	53	172	-21.73	3'UTR	mRNA
NUP210	NM_024923	1003	176	-28.62	CDS	mRNA

NUP62	NM_012346	1790	175	-38.44	CDS	mRNA
OPA3	NM_001017989	475	175	-27.80	3'UTR	mRNA
OR52E6	NM_001005167	1	181	-26.75	CDS	mRNA
OSBPL11	NM_022776	553	173	-24.72	CDS	mRNA
PARVA	NM_018222	2215	170	-26.17	3'UTR	mRNA
PAWR	NM_002583	282	171	-25.23	3'UTR	mRNA
PCSK6	NM_138325	695	180	-28.60	CDS	mRNA
PDCD11	NM_014976	1889	182	-28.57	CDS	mRNA
PDCD2	NM_002598	289	173	-25.24	3'UTR	mRNA
PDCD6IP	NM_001256192	4154	174	-27.43	3'UTR	mRNA
PDPR	NM_017990	487	171	-24.26	3'UTR	mRNA
PDZD2	NM_178140	885	174	-34.52	CDS	mRNA
PELP1	NM_001278241	1242	176	-30.10	5'UTR	mRNA
PELP1	NM_014389	1242	176	-30.10	CDS	mRNA
PER3	NM_016831	152	172	-30.06	CDS	mRNA
PGLYRP4	NM_020393	5	178	-28.69	CDS	mRNA
PHC3	NM_024947	1050	347	-49.00	3'UTR	mRNA
PHF1	NM_002636	494	176	-30.60	CDS	mRNA
PHKG2	NM_000294	778	188	-31.16	3'UTR	mRNA
PID1	NM_017933	59	172	-28.81	3'UTR	mRNA
PIK3C2G	NM_004570	72	184	-28.80	CDS	mRNA
PIK3R4	NM_014602	1200	179	-31.84	CDS	mRNA
PIWIL4	NM_152431	9	172	-34.58	5'UTR	mRNA
PKHD1L1	NM_177531	17	180	-31.83	CDS	mRNA
PLA1A	NM_001206961	62	192	-33.70	5'UTR	mRNA
PLA1A	NM_015900	62	192	-33.70	CDS	mRNA
PLD2	NM_002663	664	176	-31.24	CDS	mRNA
PLEKHG5	NM_001042665	137	185	-27.25	5'UTR	mRNA
PLEKHM3	NM_001080475	199	177	-32.33	3'UTR	mRNA
PMEPA1	NM_199171	2672	170	-28.96	3'UTR	mRNA
POLA1	NM_016937	577	172	-23.85	CDS	mRNA
POM121	NM_172020	1374	175	-25.58	CDS	mRNA
POM121C	NM_001099415	648	173	-28.28	CDS	mRNA
POMT2	NM_013382	442	184	-33.79	CDS	mRNA
PPIL2	NM_014337	1618	170	-22.07	3'UTR	mRNA
PPIP5K2	NM_015216	731	171	-24.97	3'UTR	mRNA
PPP2R1A	NM_014225	5912	171	-30.61	3'UTR	mRNA
PPRC1	NM_015062	1349	187	-34.97	CDS	mRNA
PTCHD2	NM_020780	6	170	-24.74	CDS	mRNA
PTGS1	NM_000962	330	172	-24.93	3'UTR	mRNA

PTH2R	NM_005048	22	178	-27.40	CDS	mRNA
PTPN13	NM_006264	885	174	-24.44	CDS	mRNA
PTPN14	NM_005401	128	175	-27.57	3'UTR	mRNA
PTPN4	NM_002830	234	172	-24.33	3'UTR	mRNA
PTPRJ	NM_002843	833	179	-26.89	CDS	mRNA
RAB3GAP1	NM_012233	1919	185	-30.36	CDS	mRNA
RAB7L1	NM_003929	1703	174	-21.71	3'UTR	mRNA
RALBP1	NM_006788	2378	170	-23.13	3'UTR	mRNA
RAP1GAP2	NM_015085	209	170	-24.61	3'UTR	mRNA
RBM15B	NM_013286	2602	177	-28.89	CDS	mRNA
RBMX	NM_002139	3277	178	-30.68	CDS	mRNA
RBMXL1	NM_019610	418	178	-30.68	CDS	mRNA
RCOR3	NM_001136225	965	174	-23.49	3'UTR	mRNA
RELN	NM_005045	1	171	-22.87	CDS	mRNA
RGL1	NM_015149	473	170	-28.36	3'UTR	mRNA
RGS1	NM_002922	2990	170	-22.51	3'UTR	mRNA
RNF121	NM_018320	378	172	-25.71	CDS	mRNA
RNF123	NM_022064	453	172	-20.96	3'UTR	mRNA
RNF14	NM_004290	768	173	-21.87	3'UTR	mRNA
RNF169	NM_001098638	429	196	-31.54	CDS	mRNA
RNF38	NM_022781	756	172	-30.20	3'UTR	mRNA
RRM2B	NM_015713	921	187	-23.15	3'UTR	mRNA
RSC1A1	NM_006511	218	170	-28.37	CDS	mRNA
RUNX3	NM_004350	153	174	-26.12	3'UTR	mRNA
RYR2	NM_001035	6	170	-24.18	CDS	mRNA
SBF1	NM_002972	529	172	-34.83	CDS	mRNA
SCN2A	NM_021007	10	181	-26.53	CDS	mRNA
SCN3A	NM_006922	1	179	-26.66	CDS	mRNA
SCN4A	NM_000334	19	170	-29.95	CDS	mRNA
SCN4B	NM_174934	147	171	-33.13	3'UTR	mRNA
SCN7A	NM_002976	12	170	-26.50	CDS	mRNA
SCN8A	NM_014191	68	174	-23.71	CDS	mRNA
SCN9A	NM_002977	9	171	-21.05	CDS	mRNA
SCYL3	NM_020423	666	174	-21.27	CDS	mRNA
SDCBP	NM_005625	2773	171	-30.96	CDS	mRNA
SDHC	NM_003001	3362	174	-33.35	3'UTR	mRNA
SEC24C	NM_004922	2470	182	-32.14	3'UTR	mRNA
SERAC1	NM_032861	92	173	-28.92	3'UTR	mRNA
SGMS2	NM_152621	534	349	-54.84	3'UTR	mRNA
SGPL1	NM_003901	2376	176	-28.99	CDS	mRNA

SH3TC2	NM_024577	21	172	-27.85	3'UTR	mRNA
SHROOM4	NM_020717	252	173	-23.26	CDS	mRNA
SLAMF1	NM_003037	33	172	-34.11	5'UTR	mRNA
SLC12A9	NM_020246	633	172	-27.65	CDS	mRNA
SLC16A10	NM_018593	17	200	-42.23	CDS	mRNA
SLC23A2	NM_005116	541	184	-34.20	3'UTR	mRNA
SLC24A3	NM_020689	157	170	-29.36	CDS	mRNA
SLC25A13	NM_014251	600	174	-28.93	3'UTR	mRNA
SLC25A17	NM_006358	1272	171	-25.77	CDS	mRNA
SLC25A23	NM_024103	1187	171	-25.33	3'UTR	mRNA
SLC26A2	NM_000112	484	175	-25.29	CDS	mRNA
SLC26A7	NM_052832	27	171	-28.78	3'UTR	mRNA
SLC28A3	NM_022127	253	170	-24.21	CDS	mRNA
SLC2A13	NM_052885	701	170	-24.62	3'UTR	mRNA
SLC2A4	NM_001042	20	171	-28.86	CDS	mRNA
SLC37A4	NM_001467	674	172	-31.79	CDS	mRNA
SLC39A11	NM_139177	1991	171	-30.18	3'UTR	mRNA
SLC7A1	NM_003045	804	173	-33.82	CDS	mRNA
SLCO2A1	NM_005630	241	186	-29.73	CDS	mRNA
SMNDC1	NM_005871	760	173	-28.46	3'UTR	mRNA
SMOC1	NM_022137	20	172	-24.79	3'UTR	mRNA
SPDYE3	NM_001004351	45	184	-28.66	CDS	mRNA
SPTBN1	NM_003128	3711	172	-23.74	3'UTR	mRNA
SRP54	NM_003136	1296	171	-20.48	CDS	mRNA
SUFU	NM_016169	635	172	-24.75	3'UTR	mRNA
SYN2	NM_003178	2	181	-29.57	CDS	mRNA
SYNGAP1	NM_006772	187	172	-30.22	CDS	mRNA
SYNGR2	NM_004710	6233	173	-28.03	3'UTR	mRNA
SYNRG	NM_007247	681	170	-26.78	CDS	mRNA
SYTL5	NM_138780	264	171	-22.38	3'UTR	mRNA
TAF11	NM_005643	827	175	-25.58	3'UTR	mRNA
TAS2R31	NM_176885	1	170	-20.35	CDS	mRNA
TAS2R46	NM_176887	1	170	-20.35	CDS	mRNA
TEP1	NM_007110	565	175	-28.95	CDS	mRNA
TGFBI	NM_000358	3471	176	-25.55	CDS	mRNA
THAP6	NM_144721	168	173	-21.21	3'UTR	mRNA
TJP1	NM_003257	1790	175	-31.04	CDS	mRNA
TM6SF1	NM_023003	100	172	-25.99	CDS	mRNA
TMEM165	NM_018475	2099	170	-25.00	3'UTR	mRNA
TMEM170B	NM_001100829	204	171	-23.80	3'UTR	mRNA

TMEM182	NM_144632	120	173	-28.46	CDS	mRNA
TMEM194A	NM_015257	657	172	-30.72	3'UTR	mRNA
TMEM2	NM_013390	1811	171	-31.49	3'UTR	mRNA
TMEM218	NM_001258247	268	173	-23.49	3'UTR	mRNA
TMTC2	NM_152588	259	170	-23.66	CDS	mRNA
TOR1AIP2	NM_022347	590	173	-24.95	3'UTR	mRNA
TRAK2	NM_015049	1486	176	-23.24	CDS	mRNA
TRAM1L1	NM_152402	98	176	-31.70	CDS	mRNA
TRAM2	NM_012288	1567	171	-32.85	3'UTR	mRNA
TRDMT1	NM_004412	50	174	-31.45	3'UTR	mRNA
TRDN	NM_001251987	4	171	-23.59	3'UTR	mRNA
TRIM2	NM_015271	151	179	-27.61	3'UTR	mRNA
TRIM36	NM_018700	257	174	-25.30	3'UTR	mRNA
TRRAP	NM_003496	2268	175	-24.94	CDS	mRNA
TSKU	NM_015516	1000	180	-34.36	CDS	mRNA
TSTD2	NM_139246	448	179	-21.99	3'UTR	mRNA
TTF1	NM_007344	408	175	-25.30	CDS	mRNA
TUBA3C	NM_006001	1	173	-21.12	CDS	mRNA
UBASH3B	NM_032873	87	183	-30.94	3'UTR	mRNA
UBL4A	NM_014235	1134	179	-25.47	3'UTR	mRNA
UBN2	NM_173569	599	174	-27.44	3'UTR	mRNA
UGT1A10	NM_019075	6	174	-24.83	CDS	mRNA
UGT1A7	NM_019077	1	170	-22.91	CDS	mRNA
UNC5C	NM_003728	61	170	-26.50	3'UTR	mRNA
USH2A	NM_206933	6	173	-22.89	CDS	mRNA
USP24	NM_015306	2152	173	-25.74	CDS	mRNA
USP31	NM_020718	401	351	-46.71	3'UTR	mRNA
VANGL2	NM_020335	1155	178	-30.27	3'UTR	mRNA
VN1R5	NM_173858	1	171	-21.29	CDS	mRNA
VPS13A	NM_001018038	440	175	-27.23	3'UTR	mRNA
VPS13B	NM_152564	1026	172	-24.84	CDS	mRNA
WHSC1	NM_007331	1469	178	-23.22	3'UTR	mRNA
WWP1	NM_007013	1923	176	-33.81	CDS	mRNA
XAF1	NM_017523	1143	171	-25.07	3'UTR	mRNA
XPNPEP1	NM_020383	934	174	-22.71	CDS	mRNA
XPO7	NM_015024	2560	177	-25.71	CDS	mRNA
YTHDF3	NM_152758	2502	175	-20.26	CDS	mRNA
ZNF280B	NM_080764	6	181	-27.73	3'UTR	mRNA
ZNF451	NM_001257273	522	175	-24.20	3'UTR	mRNA
ZNF484	NM_031486	73	174	-28.63	CDS	mRNA

ZNF586	NM_017652	102	182	-30.87	3'UTR	mRNA
ZNF704	NM_001033723	305	173	-31.59	3'UTR	mRNA
ZNF75A	NM_153028	680	174	-22.74	3'UTR	mRNA
ZNF813	NM_001004301	168	170	-32.71	3'UTR	mRNA
ZW10	NM_004724	493	177	-25.12	3'UTR	mRNA
ZYG11B	NM_024646	1493	173	-22.57	3'UTR	mRNA

Supplementary Table S6G: Putative DQ598677 piRNA target RNAs expressed in breast tumours. piRNAs targeting long non coding RNAs are shown in *italic*.

**Gene symbol**	**RefSeq n.er**	**Expression (normalized read count)**	**Alignment score**	**Energy (kcal/mol)**	**DQ598677 binding in RNA**	**Transcript category**
GTF2IP1	NR_002206	5050	173	-29.59	-	ncRNA (pseudogene)
LOC653653	NR_027408	57	180	-29.15	-	ncRNA (pseudogene)
SDHAP2	NR_003265	204	177	-31.35	-	ncRNA (pseudogene)
KCNQ1OT1	*NR_002728*	*79*	*175*	*-28.94*	-	*lncRNA*
LOC388692	*NR_027002*	*452*	*177*	*-31.27*	-	*lncRNA*
ABCB10	NM_012089	647	172	-28.32	CDS	mRNA
ABCC2	NM_000392	322	170	-25.23	CDS	mRNA
ABCC5	NM_005688	1645	173	-23.89	CDS	mRNA
ACE	NM_000789	724	175	-25.40	CDS	mRNA
ACTR2	NM_005722	10022	171	-23.67	CDS	mRNA
ACVR2B	NM_001106	421	178	-21.59	3'UTR	mRNA
ADAM2	NM_001464	11	170	-32.90	CDS	mRNA
AIMP1	NM_004757	1073	170	-25.80	5'UTR	mRNA
ALDH3A2	NM_000382	2015	172	-23.32	CDS	mRNA
AMOTL1	NM_130847	797	170	-21.90	3'UTR	mRNA
ANK2	NM_001148	149	172	-21.85	CDS	mRNA
AP1S2	NM_003916	263	180	-29.15	CDS	mRNA
APC	NM_000038	685	171	-21.30	CDS	mRNA
APOL1	NM_003661	3585	175	-28.61	CDS	mRNA
ARAP1	NM_015242	1398	184	-40.02	CDS	mRNA
ARV1	NM_022786	508	177	-21.59	CDS	mRNA
ASH2L	NM_004674	1009	172	-26.74	CDS	mRNA
ATP13A5	NM_198505	182	170	-25.67	CDS	mRNA

ATP9A	NM_006045	1853	172	-29.16	CDS	mRNA
ATRIP	NM_130384	233	171	-34.65	CDS	mRNA
B3GNT4	NM_030765	7	174	-30.59	3'UTR	mRNA
BCL2L15	NM_001010922	13	170	-34.58	CDS	mRNA
BDP1	NM_018429	555	175	-22.31	CDS	mRNA
BMS1	NM_014753	1396	174	-24.37	CDS	mRNA
BRD4	NM_014299	1788	180	-35.86	CDS	mRNA
BTBD2	NM_017797	1772	170	-34.70	CDS	mRNA
C5	NM_001735	38	170	-32.27	CDS	mRNA
C6orf132	NM_001164446	187	171	-23.68	3'UTR	mRNA
CAMK1G	NM_020439	7	171	-27.04	CDS	mRNA
CAPN6	NM_014289	6	172	-23.33	3'UTR	mRNA
CCDC132	NM_017667	476	171	-26.62	CDS	mRNA
CCDC54	NM_032600	1	173	-21.17	CDS	mRNA
CCDC68	NM_025214	2	175	-20.25	CDS	mRNA
CD163	NM_004244	798	181	-27.89	CDS	mRNA
CD34	NM_001025109	1580	170	-32.52	CDS	mRNA
CD34	NM_001773	1580	175	-29.23	3'UTR	mRNA
CDH18	NM_004934	1	173	-37.38	CDS	mRNA
CDHR1	NM_033100	15	170	-27.09	CDS	mRNA
CDK5RAP2	NM_018249	1649	170	-23.99	CDS	mRNA
CDON	NM_016952	133	170	-22.74	CDS	mRNA
CEBPZ	NM_005760	1404	173	-37.79	CDS	mRNA
CEL	NM_001807	3	187	-34.85	CDS	mRNA
CENPE	NM_001813	442	186	-27.20	CDS	mRNA
CNTLN	NM_017738	87	171	-24.55	CDS	mRNA
CTNND2	NM_001332	8	174	-51.68	CDS	mRNA
CUX1	NM_181552	961	174	-24.00	3'UTR	mRNA
CWC22	NM_020943	663	176	-26.61	CDS	mRNA
DBH	NM_000787	1	170	-39.59	CDS	mRNA
DDX55	NM_020936	534	191	-34.40	CDS	mRNA
DEF6	NM_022047	248	176	-28.89	CDS	mRNA
DHRS9	NM_199204	7	171	-24.87	5'UTR	mRNA
DICER1	NM_030621	1001	173	-30.49	3'UTR	mRNA
DLEC1	NM_007337	11	170	-27.44	CDS	mRNA
DLL1	NM_005618	88	179	-32.31	CDS	mRNA
DMP1	NM_004407	2	182	-37.95	CDS	mRNA
DNAH3	NM_017539	19	178	-25.46	CDS	mRNA
DSCR3	NM_006052	555	173	-30.52	CDS	mRNA
EEF1G	NM_001404	18050	172	-20.37	CDS	mRNA

EFCAB5	NM_198529	4	170	-31.09	CDS	mRNA
ENPP2	NM_006209	786	174	-33.69	CDS	mRNA
EPB41L1	NM_177996	1615	170	-36.65	3'UTR	mRNA
EPS8L1	NM_017729	357	171	-32.41	3'UTR	mRNA
ERN1	NM_001433	122	177	-40.28	CDS	mRNA
EVC	NM_153717	680	174	-27.40	CDS	mRNA
F8	NM_000132	172	170	-26.46	CDS	mRNA
FAF2	NM_014613	1660	177	-26.68	3'UTR	mRNA
FAM105A	NM_019018	204	174	-32.57	CDS	mRNA
FAM115A	NM_014719	389	170	-20.28	3'UTR	mRNA
FAM136A	NM_032822	1332	170	-36.90	3'UTR	mRNA
FAM69A	NM_001006605	295	350	-61.42	CDS	mRNA
FER	NM_005246	25	170	-25.38	CDS	mRNA
FILIP1	NM_015687	63	170	-24.19	CDS	mRNA
FOXK2	NM_004514	1661	182	-24.18	3'UTR	mRNA
FRAS1	NM_001166133	60	170	-29.94	3'UTR	mRNA
FRK	NM_002031	156	171	-30.45	3'UTR	mRNA
GAB2	NM_012296	277	176	-29.67	3'UTR	mRNA
GABRD	NM_000815	103	179	-26.85	3'UTR	mRNA
GARS	NM_002047	2466	174	-31.77	CDS	mRNA
GATAD2B	NM_020699	1167	182	-27.09	3'UTR	mRNA
GCSH	NM_004483	1	180	-23.05	3'UTR	mRNA
GPAM	NM_020918	407	170	-26.00	CDS	mRNA
GPM6A	NM_201592	2	172	-24.60	3'UTR	mRNA
GPR155	NM_152529	248	171	-38.87	CDS	mRNA
GRAMD4	NM_015124	1268	170	-32.71	CDS	mRNA
GRID1	NM_017551	42	172	-30.16	CDS	mRNA
GTF2I	NM_001518	3012	173	-29.59	CDS	mRNA
GTF2IRD2	NM_173537	118	353	-61.49	CDS	mRNA
GTF2IRD2B	NM_001003795	42	353	-61.49	CDS	mRNA
GUCY1A3	NM_000856	688	174	-30.20	3'UTR	mRNA
GXYLT2	NM_001080393	162	173	-34.65	CDS	mRNA
HADHB	NM_000183	2375	170	-24.73	CDS	mRNA
HECW1	NM_015052	15	170	-31.76	CDS	mRNA
HELB	NM_033647	50	171	-28.64	CDS	mRNA
HNRNPUL1	NM_144732	5797	171	-37.64	CDS	mRNA
IFI30	NM_006332	6459	178	-28.06	CDS	mRNA
IKZF3	NM_012481	172	178	-30.19	3'UTR	mRNA
IL15	NM_000585	42	177	-25.95	CDS	mRNA
ITM2C	NM_001287241	731	171	-31.07	5'UTR	mRNA

JAK1	NM_002227	7209	171	-27.10	CDS	mRNA
JPH1	NM_020647	3	180	-32.70	CDS	mRNA
KCNAB1	NM_003471	20	174	-30.86	3'UTR	mRNA
KCNC4	NM_004978	98	170	-32.86	CDS	mRNA
KCNMB2	NM_005832	1	171	-23.03	CDS	mRNA
KIF14	NM_014875	244	182	-32.22	3'UTR	mRNA
KLK10	NM_002776	7	175	-43.12	CDS	mRNA
LAP3	NM_015907	2749	180	-25.46	CDS	mRNA
LONP2	NM_031490	4545	180	-33.54	CDS	mRNA
LRRC42	NM_052940	1101	175	-26.76	CDS	mRNA
LRRN3	NM_018334	33	177	-40.64	5'UTR	mRNA
MACF1	NM_012090	6051	175	-27.87	CDS	mRNA
MAP3K5	NM_005923	539	174	-26.88	CDS	mRNA
MAP3K7	NM_003188	690	184	-28.29	3'UTR	mRNA
MBP	NM_002385	207	172	-27.11	3'UTR	mRNA
MCM4	NM_182746	4190	174	-27.22	3'UTR	mRNA
MDN1	NM_014611	1128	189	-30.97	CDS	mRNA
MKI67	NM_002417	2612	170	-31.43	CDS	mRNA
MRPS11	NM_176805	510	174	-40.61	5'UTR	mRNA
MSTN	NM_005259	1	180	-30.60	CDS	mRNA
MUC5B	NM_002458	20414	173	-31.46	CDS	mRNA
MUC6	NM_005961	10	175	-33.97	CDS	mRNA
MYH10	NM_005964	1800	173	-22.44	3'UTR	mRNA
MYH7B	NM_020884	13	180	-35.08	CDS	mRNA
MYO1D	NM_015194	2754	171	-31.49	CDS	mRNA
NCAPH2	NM_152299	689	170	-25.45	3'UTR	mRNA
NEB	NM_004543	14	170	-22.11	CDS	mRNA
NEFL	NM_006158	3	174	-34.15	3'UTR	mRNA
NEU1	NM_000434	1898	173	-32.19	CDS	mRNA
NOL8	NM_001256394	801	185	-35.83	5'UTR	mRNA
NOL8	NM_017948	801	185	-35.83	CDS	mRNA
NUP188	NM_015354	1342	181	-32.88	CDS	mRNA
NUP214	NM_005085	1669	170	-30.87	CDS	mRNA
OSBPL2	NM_001278649	1204	176	-29.28	5'UTR	mRNA
PCDH7	NM_032457	680	175	-27.49	3'UTR	mRNA
PCDHA4	NM_018907	138	178	-25.91	CDS	mRNA
PCDHA7	NM_018910	84	174	-24.36	CDS	mRNA
PCDHA9	NM_014005	1	174	-24.36	CDS	mRNA
PCNXL2	NM_014801	111	176	-28.81	CDS	mRNA
PDE1A	NM_005019	52	172	-27.76	CDS	mRNA

PDHB	NM_000925	1074	172	-22.90	3'UTR	mRNA
PDPN	NM_006474	767	173	-32.15	CDS	mRNA
PDSS1	NM_014317	139	177	-30.45	CDS	mRNA
PFKL	NM_002626	3655	172	-40.27	CDS	mRNA
PHF14	NM_014660	1192	179	-26.29	CDS	mRNA
PHOSPHO2	NM_001199288	50	182	-27.74	CDS	mRNA
PIAS4	NM_015897	362	176	-23.86	CDS	mRNA
PKHD1	NM_138694	6	184	-28.81	CDS	mRNA
PLCG2	NM_002661	186	171	-30.98	CDS	mRNA
PLCXD1	NM_018390	350	171	-34.17	CDS	mRNA
PM20D2	NM_001010853	89	172	-30.75	CDS	mRNA
POLH	NM_006502	520	175	-26.07	3'UTR	mRNA
POLQ	NM_199420	243	170	-20.64	3'UTR	mRNA
POLR2B	NM_000938	2410	176	-33.55	CDS	mRNA
POLR3B	NM_018082	598	173	-24.54	3'UTR	mRNA
POMC	NM_000939	2	173	-26.68	CDS	mRNA
PPEF1	NM_006240	88	170	-23.86	CDS	mRNA
PRUNE2	NM_015225	142	184	-36.13	CDS	mRNA
PTPN2	NM_002828	694	174	-33.09	CDS	mRNA
PTPN3	NM_002829	505	187	-31.71	CDS	mRNA
RABGAP1	NM_012197	1698	171	-42.71	CDS	mRNA
RAD54L	NM_003579	135	174	-24.42	CDS	mRNA
RBM27	NM_018989	782	174	-25.51	CDS	mRNA
RECK	NM_021111	425	173	-22.66	CDS	mRNA
RFX6	NM_173560	4	170	-25.65	3'UTR	mRNA
RIMS2	NM_014677	3	181	-33.18	CDS	mRNA
RNF213	NM_020954	4979	185	-33.59	CDS	mRNA
ROCK2	NM_004850	830	172	-23.90	CDS	mRNA
RTKN2	NM_145307	167	174	-28.68	CDS	mRNA
RYR1	NM_000540	33	170	-23.22	CDS	mRNA
SACS	NM_014363	536	170	-23.37	3'UTR	mRNA
SALL2	NM_005407	278	171	-32.82	CDS	mRNA
SCN2B	NM_004588	7	174	-30.22	3'UTR	mRNA
SCN9A	NM_002977	9	175	-25.87	CDS	mRNA
SFRP2	NM_003013	12452	183	-30.94	CDS	mRNA
SGSM2	NM_014853	404	172	-34.10	CDS	mRNA
SHC4	NM_203349	25	170	-30.42	CDS	mRNA
SIK1	NM_173354	1037	175	-37.29	3'UTR	mRNA
SLC23A2	NM_203327	541	199	-36.12	CDS	mRNA
SLC35E2	NM_182838	904	176	-35.74	CDS	mRNA

SLC39A8	NM_022154	283	170	-29.22	CDS	mRNA
SLC9A4	NM_001011552	2	174	-26.80	CDS	mRNA
SNX1	NM_148955	1628	183	-33.39	CDS	mRNA
SORBS2	NM_003603	223	170	-25.98	CDS	mRNA
SPATA5	NM_145207	188	176	-24.44	CDS	mRNA
STAB2	NM_017564	2	177	-37.04	CDS	mRNA
STARD13	NM_001243476	296	174	-31.15	5'UTR	mRNA
STEAP4	NM_024636	138	173	-27.95	3'UTR	mRNA
STXBP1	NM_003165	164	173	-29.35	CDS	mRNA
SYNE1	NM_033071	828	170	-20.13	CDS	mRNA
TAF1	NM_004606	1133	175	-27.25	3'UTR	mRNA
TAX1BP1	NM_001206901	3182	173	-29.26	5'UTR	mRNA
TECPR2	NM_014844	350	175	-32.83	3'UTR	mRNA
THUMPD1	NM_017736	2850	182	-26.58	3'UTR	mRNA
TLE2	NM_003260	254	171	-30.24	CDS	mRNA
TMEM150A	NM_001031738	532	171	-30.58	3'UTR	mRNA
TNFRSF10B	NM_147187	661	190	-34.82	3'UTR	mRNA
TNPO3	NM_012470	1047	177	-23.57	CDS	mRNA
TPP2	NM_003291	886	170	-30.30	CDS	mRNA
UBN1	NM_001288656	2657	173	-31.55	CDS	mRNA
USP10	NM_005153	1677	179	-28.72	CDS	mRNA
USP12	NM_182488	181	178	-21.57	CDS	mRNA
USP48	NM_032236	676	176	-20.87	CDS	mRNA
WIZ	NM_021241	1849	175	-21.93	3'UTR	mRNA
WLS	NM_024911	2117	171	-30.28	CDS	mRNA
WNT5B	NM_030775	105	170	-29.37	3'UTR	mRNA
WSB1	NM_134265	1007	171	-25.48	CDS	mRNA
ZC3HAV1	NM_020119	1419	170	-20.15	CDS	mRNA
ZDHHC14	NM_024630	45	184	-26.56	CDS	mRNA
ZKSCAN1	NM_003439	964	172	-26.79	CDS	mRNA
ZNF107	NM_016220	335	171	-23.07	CDS	mRNA
ZNF202	NM_003455	235	172	-22.24	3'UTR	mRNA
ZNF34	NM_030580	57	171	-37.80	CDS	mRNA
ZNF396	NM_145756	29	183	-25.96	3'UTR	mRNA
ZNF425	NM_001001661	19	177	-24.42	CDS	mRNA
ZNF511	NM_145806	407	175	-32.18	CDS	mRNA
ZNF521	NM_015461	317	172	-40.73	CDS	mRNA
ZNF544	NM_014480	663	175	-30.81	CDS	mRNA
ZNF555	NM_152791	55	188	-26.86	CDS	mRNA
ZNF626	NM_001076675	316	174	-20.51	CDS	mRNA

ZNF648	NM_001009992	19	186	-30.80	3'UTR	mRNA
ZNF649	NM_023074	182	171	-23.52	CDS	mRNA
ZNF720	NM_001130913	471	187	-37.64	CDS	mRNA

Supplementary Table S6H: Putative DQ597960 piRNA target RNAs expressed in breast tumours. piRNAs targeting long non coding RNAs are shown in *italic*.

**Gene symbol**	**RefSeq n.er**	**Expression (normalized read count)**	**Alignment score**	**Energy (kcal/mol)**	**DQ597960 binding in RNA**	**Transcript category**
CLK2P	NR_002711	20	176	-38.35	-	ncRNA (pseudogene)
EMR4P	NR_024075	1	171	-41.73	-	ncRNA (pseudogene)
LOC729603	NR_003288	9	171	-23.37	-	ncRNA (pseudogene)
FAM182B	*NR_026714*	*6*	*171*	*-35.48*	-	*lncRNA*
KIAA0125	*NR_026800*	*1*	*171*	*-36.61*	-	*lncRNA*
ABCA7	NM_019112	170	170	-33.44	CDS	mRNA
ABCA9	NM_080283	23	170	-25.04	CDS	mRNA
ABCB1	NM_000927	38	175	-33.09	CDS	mRNA
ADAMTS10	NM_030957	151	171	-33.76	CDS	mRNA
AHNAK	NM_001620	21685	172	-34.68	CDS	mRNA
ARHGAP1	NM_004308	4399	176	-28.88	CDS	mRNA
B4GALNT1	NM_001478	60	170	-28.62	3'UTR	mRNA
BICD2	NM_001003800	687	171	-37.07	3'UTR	mRNA
BMF	NM_033503	325	171	-28.72	3'UTR	mRNA
BNC2	NM_017637	315	180	-31.68	CDS	mRNA
C12orf76	NM_207435	124	170	-30.17	CDS	mRNA
C17orf51	NM_001113434	92	178	-28.40	3'UTR	mRNA
C19orf44	NM_032207	143	172	-36.96	CDS	mRNA
C19orf48	NM_032712	1239	172	-36.24	3'UTR	mRNA
C1QB	NM_000491	3122	172	-26.00	CDS	mRNA
C21orf59	NM_021254	746	172	-30.15	CDS	mRNA
CACHD1	NM_020925	134	182	-30.52	3'UTR	mRNA
CARM1	NM_199141	1391	173	-38.58	3'UTR	mRNA

CCBE1	NM_133459	6	170	-33.32	3'UTR	mRNA
CCBL1	NM_004059	57	171	-32.57	CDS	mRNA
CCM2	NM_031443	741	174	-29.90	CDS	mRNA
CD93	NM_012072	2317	180	-36.15	CDS	mRNA
CDC20	NM_001255	552	174	-39.13	CDS	mRNA
CEBPG	NM_001806	848	172	-27.58	3'UTR	mRNA
CGNL1	NM_032866	374	175	-30.94	3'UTR	mRNA
CHIT1	NM_003465	31	177	-30.90	CDS	mRNA
COPS3	NM_003653	714	174	-24.65	CDS	mRNA
CRB2	NM_173689	2	180	-37.63	CDS	mRNA
CYP20A1	NM_177538	440	182	-36.00	3'UTR	mRNA
CYP46A1	NM_006668	6	171	-24.47	CDS	mRNA
DEDD2	NM_133328	470	173	-32.65	3'UTR	mRNA
DENND1C	NM_024898	143	172	-37.16	CDS	mRNA
DNAH3	NM_017539	19	187	-39.40	CDS	mRNA
DUSP13	NM_001007271	3	172	-34.23	3'UTR	mRNA
EMP2	NM_001424	6842	170	-28.38	3'UTR	mRNA
EMR2	NM_013447	75	183	-35.87	CDS	mRNA
EPS15L1	NM_001258376	748	170	-25.96	3'UTR	mRNA
ERBB4	NM_005235	1013	171	-29.35	3'UTR	mRNA
ESCO1	NM_052911	472	170	-22.42	CDS	mRNA
FAM83H	NM_198488	2163	175	-35.11	3'UTR	mRNA
FBLN2	NM_001165035	2311	170	-25.85	5'UTR	mRNA
FDPS	NM_002004	4204	172	-32.85	CDS	mRNA
FGFR4	NM_002011	890	188	-40.89	3'UTR	mRNA
FOXK2	NM_004514	1661	170	-30.36	3'UTR	mRNA
GABRA5	NM_000810	2	171	-30.31	CDS	mRNA
GAS7	NM_201433	899	175	-41.25	3'UTR	mRNA
GBP2	NM_004120	2832	171	-32.96	3'UTR	mRNA
GMPR2	NM_016576	971	170	-30.08	3'UTR	mRNA
GNE	NM_005476	417	188	-32.88	3'UTR	mRNA
GON4L	NM_032292	1627	178	-39.37	CDS	mRNA
GPR17	NM_005291	3	183	-39.29	CDS	mRNA
GPR179	NM_001004334	7	172	-29.60	CDS	mRNA
GSDMD	NM_024736	994	182	-37.72	3'UTR	mRNA
GXYLT1	NM_173601	610	173	-31.98	3'UTR	mRNA
HERC2	NM_004667	688	340	-55.65	CDS	mRNA
HLA-DPB1	NM_002121	5032	184	-31.04	3'UTR	mRNA
HMCN1	NM_031935	1352	182	-36.56	CDS	mRNA
HMHA1	NM_001282334	582	170	-34.54	5'UTR	mRNA

HRNR	NM_001009931	6	342	-61.78	CDS	mRNA
HSPD1	NM_002156	16965	176	-35.88	CDS	mRNA
IER2	NM_004907	1217	181	-34.68	3'UTR	mRNA
IFI44L	NM_006820	823	173	-29.80	CDS	mRNA
INTS3	NM_023015	3484	174	-32.26	3'UTR	mRNA
INTS5	NM_030628	682	182	-37.14	CDS	mRNA
ITIH1	NM_002215	1	177	-30.69	CDS	mRNA
ITPR1	NM_002222	900	173	-31.04	CDS	mRNA
KIF13A	NM_022113	1168	182	-38.69	CDS	mRNA
KLF2	NM_016270	631	170	-33.95	CDS	mRNA
KLK2	NM_005551	3	172	-34.80	CDS	mRNA
KLK2	NM_001002231	3	172	-34.80	3'UTR	mRNA
LAD1	NM_005558	7409	174	-36.26	3'UTR	mRNA
LAMB1	NM_002291	4289	174	-31.18	CDS	mRNA
LGALS3BP	NM_005567	12161	173	-38.57	CDS	mRNA
LIMD2	NM_030576	216	172	-35.48	3'UTR	mRNA
LIPH	NM_139248	90	174	-30.43	3'UTR	mRNA
LIX1L	NM_153713	378	178	-30.43	CDS	mRNA
LRAT	NM_004744	2	170	-29.90	CDS	mRNA
LRGUK	NM_144648	18	182	-32.84	CDS	mRNA
LRRC41	NM_006369	1551	176	-30.71	CDS	mRNA
MAP2	NM_031847	62	170	-32.29	CDS	mRNA
MAST2	NM_015112	1059	173	-27.94	CDS	mRNA
MBNL1	NM_021038	2208	179	-34.85	3'UTR	mRNA
MBP	NM_001025100	207	170	-24.29	3'UTR	mRNA
MECP2	NM_004992	818	177	-33.21	3'UTR	mRNA
MEX3C	NM_016626	888	177	-35.76	5'UTR	mRNA
MGAM	NM_004668	3	345	-63.02	CDS	mRNA
MLLT1	NM_005934	2014	170	-31.08	3'UTR	mRNA
MRC2	NM_006039	4265	176	-39.15	CDS	mRNA
MTMR4	NM_004687	2040	173	-27.71	CDS	mRNA
MUC16	NM_024690	13	171	-29.62	CDS	mRNA
MYEOV	NM_138768	3	170	-28.49	3'UTR	mRNA
MYO1E	NM_004998	1238	174	-30.57	3'UTR	mRNA
NCAM1	NM_001076682	44	183	-43.93	3'UTR	mRNA
NCOR2	NM_006312	2499	175	-33.58	CDS	mRNA
NPR1	NM_000906	92	174	-28.60	CDS	mRNA
NT5DC3	NM_001031701	264	172	-27.15	3'UTR	mRNA
ODF3	NM_053280	2	173	-31.40	CDS	mRNA
OSGIN1	NM_182981	62	176	-40.08	CDS	mRNA

PAPL	NM_001004318	2	172	-30.22	CDS	mRNA
PATZ1	NM_014323	2226	179	-42.53	CDS	mRNA
PATZ1	NM_032052	2226	179	-42.53	3'UTR	mRNA
PGLYRP2	NM_052890	16	176	-34.48	CDS	mRNA
PIGV	NM_017837	319	173	-28.80	CDS	mRNA
PLEKHG4B	NM_052909	119	171	-28.44	3'UTR	mRNA
POFUT2	NM_015227	446	177	-36.49	CDS	mRNA
POFUT2	NM_015227	446	173	-35.77	3'UTR	mRNA
POLR3E	NM_018119	1386	170	-33.14	3'UTR	mRNA
POM121	NM_172020	1374	170	-28.46	CDS	mRNA
POM121C	NM_001099415	648	170	-28.46	CDS	mRNA
PPP1R12B	NM_002481	2411	170	-29.21	3'UTR	mRNA
PPP1R9B	NM_032595	2773	171	-35.87	3'UTR	mRNA
PRCP	NM_005040	3205	175	-28.08	3'UTR	mRNA
PRKRIP1	NM_024653	482	180	-34.73	CDS	mRNA
PTP4A3	NM_007079	427	180	-33.72	CDS	mRNA
PYGO1	NM_015617	9	170	-27.62	CDS	mRNA
QARS	NM_005051	2817	181	-29.84	CDS	mRNA
RASEF	NM_152573	194	176	-32.66	CDS	mRNA
RASSF10	NM_001080521	11	180	-34.93	CDS	mRNA
RHBDL3	NM_138328	44	171	-33.66	3'UTR	mRNA
RHOD	NM_014578	314	174	-35.55	CDS	mRNA
RNF32	NM_030936	28	170	-30.83	CDS	mRNA
RSBN1L	NM_198467	339	175	-29.83	CDS	mRNA
SCAP	NM_012235	1070	172	-40.29	CDS	mRNA
SEMA4B	NM_020210	2070	172	-32.68	CDS	mRNA
SERPINA1	NM_000295	950	174	-31.22	3'UTR	mRNA
SERPINE2	NM_006216	725	172	-31.57	CDS	mRNA
SLC10A6	NM_197965	9	172	-28.70	CDS	mRNA
SLC2A10	NM_030777	1595	172	-31.79	CDS	mRNA
SLC35B2	NM_178148	2190	171	-37.77	CDS	mRNA
SLC6A7	NM_014228	3	178	-32.52	CDS	mRNA
SLIT1	NM_003061	3	172	-31.87	3'UTR	mRNA
STK11IP	NM_052902	406	170	-36.83	CDS	mRNA
TAPBP	NM_172209	5593	171	-32.93	CDS	mRNA
TBX15	NM_152380	150	170	-32.61	CDS	mRNA
TDRD9	NM_153046	272	171	-33.97	CDS	mRNA
TMCO6	NM_018502	84	176	-32.11	CDS	mRNA
TMEM201	NM_001010866	310	171	-35.66	CDS	mRNA
TNFRSF8	NM_001243	17	188	-40.36	CDS	mRNA

TRAF3	NM_003300	172	170	-37.44	3'UTR	mRNA
TRAM2	NM_012288	1567	170	-27.85	3'UTR	mRNA
TRIB3	NM_021158	1348	173	-32.13	3'UTR	mRNA
TRIOBP	NM_007032	1212	185	-34.26	3'UTR	mRNA
TTC28	NM_001145418	328	171	-35.17	CDS	mRNA
TTC36	NM_001080441	80	177	-36.30	CDS	mRNA
UBE3C	NM_014671	2310	172	-39.81	3'UTR	mRNA
UST	NM_005715	159	172	-31.41	CDS	mRNA
VWDE	NM_001135924	9	171	-31.12	CDS	mRNA
ZEB2	NM_014795	643	184	-37.31	CDS	mRNA
ZMYM6	NM_007167	244	180	-35.86	CDS	mRNA
ZNF341	NM_032819	186	176	-27.35	3'UTR	mRNA
ZNF35	NM_003420	108	173	-32.34	CDS	mRNA
ZNF502	NM_033210	121	170	-35.70	3'UTR	mRNA
ZNF7	NM_001282796	386	171	-28.47	3'UTR	mRNA

Supplementary Table S6I: Putative DQ570994 piRNA target RNAs expressed in breast tumours. piRNAs targeting long non coding RNAs are shown in *italic*.

**Gene symbol**	**RefSeq n.er**	**Expression (normalized read count)**	**Alignment score**	**Energy (kcal/mol)**	**DQ570994 binding in RNA**	**Transcript category**
GBAP1	NR_002188	1872	177	-28.73	-	ncRNA (pseudogene)
LOC730668	NR_027240	2	170	-30.40	-	ncRNA (pseudogene)
FLJ12825	*NR_026655*	*10*	*177*	*-31.93*	-	*lncRNA*
KCNQ1OT1	*NR_002728*	*79*	*176*	*-27.27*	-	*lncRNA*
LOC91450	*NR_026998*	*1*	*173*	*-35.59*	-	*lncRNA*
ACACA	NM_198839	3161	172	-30.95	3'UTR	mRNA
ATP5A1	NM_004046	8499	173	-32.26	CDS	mRNA
ATP6V1C1	NM_001695	1877	175	-33.42	3'UTR	mRNA
B4GALNT2	NM_153446	2	171	-35.95	CDS	mRNA
BEND7	NM_152751	51	172	-26.84	CDS	mRNA
C1orf21	NM_030806	2185	180	-29.82	3'UTR	mRNA
C20orf194	NM_001009984	245	171	-31.57	CDS	mRNA
CARHSP1	NM_001278264	2006	181	-36.54	5'UTR	mRNA
CARS2	NM_024537	599	173	-28.43	CDS	mRNA
CBLB	NM_170662	610	181	-35.91	CDS	mRNA
CCDC114	NM_144577	7	179	-35.39	3'UTR	mRNA

CCR10	NM_016602	6	171	-32.33	CDS	mRNA
CHST1	NM_003654	1605	175	-34.57	3'UTR	mRNA
COL17A1	NM_000494	429	175	-31.15	CDS	mRNA
CUBN	NM_001081	30	177	-32.76	CDS	mRNA
DCLRE1C	NM_022487	263	175	-29.26	CDS	mRNA
DDX31	NM_022779	469	174	-35.49	CDS	mRNA
DNAJB2	NM_006736	1543	173	-35.54	3'UTR	mRNA
DOC2A	NM_003586	7	171	-25.86	CDS	mRNA
DUSP15	NM_080611	77	173	-34.08	5'UTR	mRNA
EFR3B	NM_014971	41	190	-39.41	3'UTR	mRNA
EHMT2	NM_025256	1547	172	-30.75	3'UTR	mRNA
ENDOD1	NM_015036	730	170	-37.74	CDS	mRNA
FAM120A	NM_001286723	5286	172	-38.08	3'UTR	mRNA
FAM172A	NM_032042	359	172	-29.78	CDS	mRNA
FAM178A	NM_001243770	674	170	-37.34	3'UTR	mRNA
FBXL12	NM_017703	289	173	-30.18	CDS	mRNA
FGD3	NM_001083536	117	172	-28.55	5'UTR	mRNA
FLCN	NM_144997	283	170	-31.30	3'UTR	mRNA
FOXD2	NM_004474	45	171	-27.42	5'UTR	mRNA
GBA	NM_000157	6219	177	-28.73	3'UTR	mRNA
GOLPH3L	NM_018178	1803	172	-26.72	3'UTR	mRNA
GPC1	NM_002081	1064	171	-31.05	3'UTR	mRNA
GPR114	NM_153837	9	171	-38.50	3'UTR	mRNA
GRHL2	NM_024915	1356	171	-30.25	3'UTR	mRNA
HPN	NM_182983	866	185	-35.59	CDS	mRNA
HTT	NM_002111	1429	182	-37.78	3'UTR	mRNA
IGF1	NM_000618	247	171	-28.37	3'UTR	mRNA
IL27	NM_145659	2	170	-30.42	3'UTR	mRNA
INSRR	NM_014215	4	173	-37.70	CDS	mRNA
IQGAP2	NM_006633	655	170	-22.67	CDS	mRNA
ITIH1	NM_001166434	1	170	-34.31	5'UTR	mRNA
ITIH1	NM_002215	1	170	-34.31	CDS	mRNA
ITSN2	NM_147152	738	174	-34.67	3'UTR	mRNA
KIAA1841	NM_001129993	159	170	-30.77	5'UTR	mRNA
LARP1	NM_015315	3980	170	-33.89	3'UTR	mRNA
LGR6	NM_021636	12	172	-33.49	3'UTR	mRNA
LIMK1	NM_002314	1174	177	-35.14	3'UTR	mRNA
LPIN1	NM_145693	661	172	-26.56	CDS	mRNA
LPP	NM_001167672	389	170	-30.21	CDS	mRNA
LRIG1	NM_015541	2412	170	-27.65	CDS	mRNA

LRPAP1	NM_002337	1994	174	-39.37	3'UTR	mRNA
LRRC8A	NM_019594	1795	172	-31.37	3'UTR	mRNA
MAP3K2	NM_006609	704	183	-35.49	3'UTR	mRNA
MIOS	NM_019005	427	173	-29.99	CDS	mRNA
MKNK1	NM_003684	406	174	-39.12	CDS	mRNA
MRFAP1L1	NM_203462	1590	174	-26.32	3'UTR	mRNA
MRPL40	NM_003776	758	180	-34.02	CDS	mRNA
MYO19	NM_025109	1644	176	-26.93	3'UTR	mRNA
NFKBIE	NM_004556	358	172	-34.34	3'UTR	mRNA
NUPL2	NM_007342	211	186	-33.59	CDS	mRNA
NXPH3	NM_007225	163	175	-34.42	3'UTR	mRNA
OBSCN	NM_001271223	39	171	-34.90	CDS	mRNA
OXCT2	NM_022120	13	171	-33.78	3'UTR	mRNA
PARVG	NM_022141	162	181	-38.64	3'UTR	mRNA
PDE1B	NM_000924	136	170	-28.62	3'UTR	mRNA
PDZD2	NM_178140	885	172	-36.61	CDS	mRNA
PKD1	NM_000296	901	171	-31.12	CDS	mRNA
PRKAG3	NM_017431	7	171	-30.78	3'UTR	mRNA
PRSS22	NM_022119	64	171	-37.51	CDS	mRNA
PSMD9	NM_002813	1082	172	-34.25	3'UTR	mRNA
PUS1	NM_025215	910	173	-33.77	5'UTR	mRNA
RANBP3	NM_003624	1018	176	-37.67	5'UTR	mRNA
RASSF1	NM_170714	351	176	-33.18	3'UTR	mRNA
RHBDL2	NM_017821	188	170	-26.86	5'UTR	mRNA
RTEL1	NM_016434	208	170	-34.39	5'UTR	mRNA
SCAF1	NM_021228	1244	173	-31.38	CDS	mRNA
SCARF2	NM_182895	360	171	-37.16	3'UTR	mRNA
SCNN1A	NM_001159576	1972	171	-31.83	CDS	mRNA
SHROOM4	NM_020717	252	172	-30.94	CDS	mRNA
SLC6A6	NM_003043	2544	174	-38.59	3'UTR	mRNA
SNAI3	NM_178310	12	171	-37.55	3'UTR	mRNA
SOCS4	NM_080867	497	178	-29.10	CDS	mRNA
SRGAP1	NM_020762	337	171	-27.58	CDS	mRNA
SSPO	NM_198455	10	178	-31.88	CDS	mRNA
STAR	NM_000349	4	179	-33.33	CDS	mRNA
STX16	NM_003763	1149	175	-34.85	5'UTR	mRNA
TARS2	NM_025150	1125	171	-29.97	CDS	mRNA
TBC1D20	NM_144628	1266	177	-33.60	3'UTR	mRNA
TCP10L	NM_144659	4	174	-29.39	3'UTR	mRNA
TLR9	NM_017442	8	173	-31.00	CDS	mRNA

TMED1	NM_006858	522	176	-33.83	3'UTR	mRNA
TMEM184B	NM_012264	2108	170	-32.10	3'UTR	mRNA
TMEM44	NM_138399	152	172	-31.19	CDS	mRNA
TMEM63A	NM_014698	1219	178	-37.15	3'UTR	mRNA
TNNI1	NM_003281	74	188	-36.57	3'UTR	mRNA
TTC39A	NM_001080494	891	171	-33.99	CDS	mRNA
TWF2	NM_007284	947	175	-32.96	3'UTR	mRNA
ULK2	NM_014683	681	183	-34.76	CDS	mRNA
UMODL1	NM_173568	76	171	-26.71	CDS	mRNA
VNN2	NM_078488	54	171	-29.36	3'UTR	mRNA
VSIG10	NM_019086	606	174	-31.48	CDS	mRNA
XRCC3	NM_005432	205	172	-28.45	3'UTR	mRNA
YKT6	NM_006555	2363	171	-37.45	3'UTR	mRNA
YPEL4	NM_145008	12	183	-47.88	3'UTR	mRNA
ZNF233	NM_181756	14	173	-31.89	CDS	mRNA
ZNF707	NM_173831	201	179	-32.49	3'UTR	mRNA

Supplementary Table S7: Functional annotation analysis of biological processes involving the mRNAs targeted by 8 piRNAs differentially expressed in paired normal and tumor breast tissue samples identified in this study. For each biological process, all mRNAs involved are shown.

**Biological process**	**Transcripts**	**n. of transcripts**
Cancer	ABCB1, ABCC5, ADAM10, AHNAK, ALDOC, ARHGAP11A, ARHGAP20, ARHGEF11, ARMC8, ARMCX5, ASH2L, ATP10D, ATP5A1, BBS9, BICD2, BSN, BTN3A3, C1GALT1, C1S, CARS, CBX5, CC2D1B, CCM2, CEP350, CHTF18, CHTF8, CSGALNACT2, CUX1, DAAM1, DCLRE1C, DGKI, DHRS12, DNAH3, DOT1L, DR1, DSCR3, EHMT2, EPB41L1, ESR1, F5, FAM102A, FEN1, FOXK2, FRK, FURIN, G3BP1, G6PD, GLS, GPAM, GPC1, GPT2, GUCY1A3, HDLBP, HIC2, HIF1A, HIP1, HK2, HNRNPUL1, HS2ST1, HTT, IGF1, KCNAB1, KCTD3, KIAA0196, KRAS, LAG3, LAP3, LONP1, LPIN2, LPP, LRIG1, LRP2, LRRN3, MAST3, MBNL1, MECP2, MED23, MIA3, MKI67, MLLT1, MYO1D, NCAPH2, NCDN, NCOA6, NEFL, NOS3, NOTCH2, NPAS1, NRXN3, NSD1, NUCKS1, NUP188, OSGIN1, PAK7, PELP1, PHF20, PID1, PLA1A, POLA1, POLH, POLR2B, POMC, POMT2, PRKDC, PTPN4, PTPRJ, RABGAP1, RASSF1, RBMX, RBPJ, RCOR1, RCOR3, RECK, RELN, RNF123, RNF14, ROCK2, RYR3, SDC3, SFRP1, SFRP2, SLC2A4, SLC39A8, SLCO3A1, SNX1, SPAG9, TFDP2, TMEM2, TMTC2, TPP2, TRANK1, TRIM2, UBA52, UBN1, UMODL1, USP10, USP24, VPS13B, VPS13C, WEE1, WIZ, XRCC5, ZDHHC21	143
Cell-To-Cell Signaling and Interaction	ABCB6, ABCC1, ABI1, ACTR2, ADAMTS7, ADORA3, AGAP2, AIMP1, ALDH3A2, ARF4, ARHGAP1, ARHGEF5, ASAP2, ATP9A, C12orf76, C5, CALCOCO1, CCDC144B, CCL18, CCR10, CD163, CD34, CDH3, CDH4, CDK5RAP2, CHRM1, CHST2, COL13A1, COL7A1, CPEB1, CR1, CTNND2, CYP46A1, DBH, DDC, DHCR7, DHRS2, DICER1, DLC1, DNTTIP1, EIF1AX, ELF3, ENDOD1, ENPP2, EPS8L1, EZR, FGF12, FGFR4, FPR1, GBP2, GCH1, GPR126, GRN, HEXB, IKZF3, IL21R, IL34, IRAK1, IRAK2, ITGAV, ITGB2, ITGB5, ITPR1, ITSN1, JARID2, KLF13, KLK2, KRT18, KRT80, LEF1, LGALS8, LIPG, LRIG1, LRRC32, LSS, MAN1A1, MGRN1, MKL1, MLF2, MN1, MRAS, MSTN, MUC6, MYB, NCKAP1, NDFIP2, NEU1, NFKBIE, NID1, NKD1, P2RY2, PARVA, PARVG, PCDH19, PCDH7, PCSK6, PDE4D, PDPN, PFKL, PGC, PGR, PKD1, PLXNA1, PTGS1, PTP4A3, PTPRE, PXN, RAPH1, RASSF1, RTN1, RYR1, RYR2, SDCBP, SELP, SERF2, SERPINA1, SIT1, SLC26A2, SLC7A1, STX16, SYNPO, TENC1, TLR9, TNF, TNFRSF8, TRAF3, TRRAP, TSPYL2, VANGL2, VCL, WNT5B, ZEB2	132

Cell Death and Survival	ABCC4, ADAM15, AKR1C1/AKR1C2, AKT3, ALDOC, ANGPT2, APLNR, APOL1, ASH2L, AUTS2, BCL2L11, BICD2, BIRC5, BMF, C5, CARS, CCL18, CD163, CHST2, CHTF18, CHTF8, CR1, CRKL, DAXX, DCLRE1C, DHX58, ELF3, ELK4, ERBB4, ERN1, FEN1, FOSL1, FOXK2, FURIN, GARS, GCH1, GLE1, GNE, GPR126, HEXB, HIF1A, HIP1, HK2, HTT, IFIT2, IL15, IL21R, IL7, IRAK1, IRAK2, ITPR1, JAK1, JARID2, KLHL2, KRAS, LAD1, LIPG, LRIG1, LSS, MAP3K11, MAP3K5, MRAS, NCOA6, NFKBIE, NID1, NOTCH2, NUCKS1, NUPL2, OSGIN1, PDPK1, PDPN, PELP1, PLCG2, POLH, POLR2B, POMC, PPP2R1A, PRKDC, PSAT1, PTGS1, PTPN13, QARS, RAPH1, RASSF1, RBPJ, RUNX3, SCN3A, SLC16A6, SLC4A7, SLC7A1, STX16, SYNPO, SYT11, TAF1, TAF11, TFDP2, TINAGL1, TLR9, TNF, TRAF3, TRIB3, TRIOBP, TSLP, TTC28, TYK2, USP12, WEE1, XRCC5, ZC3H12A, ZC3HAV1	110
Cell Cycle	AMOTL1, ARHGAP20, ARMC8, ARMCX5, ATP10D, ATRIP, BBS9, BCL11B, C12orf76, C1GALT1, CALCOCO1, CARD10, CC2D1A, CC2D1B, CCM2, CDK5RAP2, CPEB1, DAAM1, DES, EHMT2, ENDOD1, ENPP2, EPB41L1, ERAP1, FAF1, FRK, GPC1, GUCY1A3, HERC2, HRH3, HSPD1, IFI30, IKZF3, JKAMP, KLF2, KRT80, MIA3, MLF2, MTA3, MYO1D, MYO1E, NCOR2, NEFL, NPAS1, NRXN3, NUP62, PDCD11, PFKL, PGC, PHF20, PIAS4, PLXNA1, PPP4R1, PTPN4, PTPRJ, RASSF1, RBMX, RCOR1, RCOR3, RECK, RNF31, ROCK2, RYR3, SERF2, SFRP2, SLAMF1, SPAG9, STAR, TAX1BP1, UBN1, VANGL2, VCAM1	72
Cellular Assembly and Organization	ABCB11, ABCC8, ACO2, ANK2, APC, APLP2, ARF1, ARFIP1, CEL, COL18A1, DIO2, DTNA, F2RL1, FES, FLNA, GBA, HIGD1A, IL27, INSR, LIMK1, LIMK2, MAP3K7, MAPKAP1, MAPKAPK3, MAPT, MKNK1, MTOR, NPR1, NT5C2, PAWR, PDCD6IP, PDHB, PDZD2, PLD2, PODXL, PSEN1, PSMC4, PSMD1, PTPN2, SCAMP1, SCNN1A, SCNN1B, SPRY2, SPTBN1, TJP1, TNRC6A, TRO, TTBK1, USP18, XAF1, XRCC3	51
Cell Morphology	ACTA2, AKR1C1/AKR1C2, AKT3, ANGPT2, ANXA6, APLNR, ARHGEF12, AXIN2, BCL2L11, BIRC5, BMF, BMP7, BMPER, CEBPZ, CSPG4, DAXX, DMP1, DPYSL2, EFNB2, ELK4, ERBB4, FAF2, FBLN2, FOSL1, GAB2, GARS, GLE1, GNE, HGF, IGF1, ITGA3, JAK1, KCNMA1, MAP3K11, MAP3K5, MUC5B, NUPL2, PAK2, PDPK1, PSAT1, PTPN13, QARS, RUNX2, RUNX3, SACS, SIM2, SLC4A7, TRDN, TRIB3, USP12	50
Cellular Movement	ACTA2, ADAMTS7, ADORA3, AGAP2, ASAP2, BMP1, CENPE, CHRM1, CLEC2D, CYP46A1, DLC1, FGFR4, FPR1, GOPC, GRN, IL34, ITGA3, ITGAV, ITGB2, ITGB5, ITSN1, LGALS8, MFN1, MGRN1, NEU1, P2RY2, PARVA, PARVG, PINK1, PMEPA1, PTP4A3, PTPRE, PXN, RHOD, SDCBP, SLC9A2, SLC9A3, SMTN, STRADA, TENC1, TGFBI, TSPYL2, VCL	43

Tissue Morphology	ACTA2, AKNA, ALDH6A1, ANXA6, ARHGEF12, AXIN2, BMP7, BMPER, CEBPG, CEBPZ, CSPG4, DMP1, DPYSL2, EFNB2, EMP2, FAF2, FBLN2, GAB2, GABRE, GPR98, GRHL2, HGF, IGF1, ITGA3, KCNMA1, MED13, MUC5B, NDUFV3, NUP210, PAK2, PCDHA9, PTBP2, RUNX2, SACS, SIM2, SLC37A4, SORCS1, TRDN, TRIB3, VAPA, YKT6	41
Gene Expression	ABL2, ARMCX5, ATP10D, BBS9, CCM2, CDC23, EHMT2, EPB41L1, FRK, GGCX, GPC1, GRIA4, HOMER1, IKZF2, MEX3C, MIA3, MYH10, NEFL, NRXN3, PHF20, PSMD9, PTPRJ, RAB34, RCOR1, RCOR3, RGS1, SCN2A, SFRP2, SLC35B2, SLC6A6, SPAG9, TEP1	32
DNA Replication, Recombination and Repair	ALDOC, ASH2L, BICD2, CARS, CHTF18, CHTF8, DCLRE1C, FEN1, FOXK2, FURIN, HIF1A, HIP1, HK2, HTT, KRAS, NCOA6, NOTCH2, NUCKS1, OSGIN1, PELP1, POLH, POLR2B, POMC, PRKDC, RBPJ, TFDP2, WEE1, XRCC5	28
Cell Signaling	ARHGEF11, ATP5A1, BSN, DNAH3, G3BP1, HDLBP, KCNAB1, KCTD3, KIAA0196, NCDN, PAK7, PLA1A, SDC3, SLCO3A1, SNX1, UBA52, USP10, USP24	18
Organ Morphology	AKNA, ALDH6A1, CEBPG, EMP2, GABRE, GPR98, GRHL2, MED13, NDUFV3, NUP210, PCDHA9, PTBP2, SLC37A4, SORCS1, TRIB3, VAPA, YKT6	17
Tumor Morphology	ARHGAP20, ARMC8, C1GALT1, CC2D1B, DAAM1, GUCY1A3, MYO1D, NPAS1, PTPN4, PTPRJ, RASSF1, RBMX, RECK, ROCK2, RYR3, UBN1	16
